# Supplementary material for: Bayesian data driven modelling of kinetochore dynamics: Space-time organisation of the human metaphase plate
Source: PLoS Comput Biol. 2026 Jan 22;22(1):e1013884. doi: 10.1371/journal.pcbi.1013884 (PMC12935310; doi:10.1371/journal.pcbi.1013884)
Supplement: S1 Appendix — (PDF) [file pcbi.1013884.s001.pdf]

S1 Appendix for: "Bayesian data driven modelling of kinetochore dynamics: space-time organisation of the human metaphase plate"

Constandina Koki[1], Alessio V. Inchingolo[2], Abdullahi Daniyan[1], Enyu Li[1], Andrew D. McAinsh[2*], Nigel J. Burroughs[1**]

1 Zeeman Institute (SBIDER), Mathematics Institute, University of Warwick, Coventry, United Kingdom
2 Centre for Mechanochemical Cell Biology, Warwick Biomedical Sciences, Warwick Medical School, University of Warwick, Coventry, United Kingdom

A Additional tables

Models	v_-, v_+, τ	v_-, v_+	v_-	v_+	**Totals**
**Strength of Asymmetry**	**DMSO**				
Substantial	1	24	36	13	74
Strong	1	27	32	8	68
Decisive	0	37	15	1	53
**Substantial+**	2 (1.03%)	88 (45.13%)	83 (42.56%)	22 (11.28%)	195
	**Nocodazole washout**				
Substantial	0	23	35	29	87
Strong	0	39	33	15	87
Decisive	2	56	47	7	112
**Substantial+**	2 (0.7%)	118 (41.46%)	115 (40.07%)	51 (17.77%)	286

Table A. Support for asymmetry models in DMSO and nocodazole washout treatment. Number of KT pairs that prefer each asymmetric model divided into strength-of-evidence categories, based on Bayes Factors [42]. Substantial+ combines all categories with at least substantial evidence (substantial, strong, or decisive). Asymmetric sisters are most often asymmetric on v_-.

**Parameter**	**Variance**				
	Total	Between-cell	Between-location	Between cell/location	sd/mean
α	4.0×10^{-5}	$2.3 \times 10^{-6*}$	4.6×10^{-8}	50.256	0.508
κ	2.1×10^{-4}	$3.6 \times 10^{-5*}$	$2.9 \times 10^{-5*}$	1.256	0.578
L	9.0×10^{-4}	$1.1 \times 10^{-4*}$	$9.9 \times 10^{-6*}$	11.416	0.037
τ	3.2×10^{4}	$1.9 \times 10^{4*}$	$3.1 \times 10^{3*}$	5.987	0.260
v_-	1.8×10^{-4}	$2.3 \times 10^{-5*}$	$4.1 \times 10^{-5*}$	0.569	0.303
v_+	3.4×10^{-5}	$9.7 \times 10^{-6*}$	$9.0 \times 10^{-7*}$	10.723	0.562
p_{coh}	2.3×10^{-4}	$3.5 \times 10^{-5*}$	$2.4 \times 10^{-5*}$	1.451	0.279
p_{incoh}	1.2×10^{-2}	$1.7 \times 10^{-3*}$	$3.0 \times 10^{-3*}$	0.582	0.149

Table B. K-fiber force variation within cells is larger than between cells. Variance contributions of the biophysical parameters, ($n = 635$ KT pairs from $N = 24$ cells, allowing at least 3 sister pairs in each group and removing outliers), grouping by cell and location (3 radius group classes) within the metaphase plate. The second column denotes the within-cell variance (i.e., residuals), while the third and fourth columns report the within-cell and location variance, respectively. Parameter estimations use the model with asymmetry on v_- and v_+; the posterior median v_- and v_+ values are averaged over the two sisters. Stars denote statistically significant difference ($\alpha < 0.01$) between groups of this factor. The ratio of the between-cell to the between-location variance is shown in the fifth column and the size of effect (ratio of population parameters' standard deviation to the population parameters' absolute mean) in the last column. Stars denote statistically different ratios at $\alpha < 0.01$. Caution: Due to low coverage of KT pairs per cell within each radius group, these results should be interpreted cautiously as some ANOVA assumptions are violated.

Parameter	Variance				
	Total	Between-cell	Between-location	Between cell/location	sd/mean
α	3.6×10^{-5}	$3.3 \times 10^{-6*}$	5.0×10^{-7}	6.57	0.393
κ	1.8×10^{-4}	$4.4 \times 10^{-5*}$	$4.2 \times 10^{-5*}$	1.045	0.660
L	8.9×10^{-4}	8.9×10^{-5}	$2.1 \times 10^{-5*}$	4.233	0.037
τ	2.0×10^{4}	$7.2 \times 10^{3*}$	$2.6 \times 10^{2*}$	2.748	0.171
v_-	1.9×10^{-4}	$4.9 \times 10^{-5*}$	$5.4 \times 10^{-5*}$	0.897	0.321
v_+	2.8×10^{-5}	$8.8 \times 10^{-6*}$	$1.7 \times 10^{-6*}$	5.124	0.589
p_{coh}	1.7×10^{-4}	$3.7 \times 10^{-5*}$	$1.8 \times 10^{-5*}$	2.108	0.014
p_{incoh}	1.3×10^{-2}	$1.7 \times 10^{-3*}$	$4.4 \times 10^{-3*}$	0.379	0.149

Table C. Variance contributions of the biophysical parameters, ($n = 290$ KT pairs from $N = 11$ cells to allow for at least 3 sister pairs in each group), grouping by cell and location (3 radius group classes) within the metaphase plate in nocodazole washout treated cells. The second column denotes the total variance (*i.e.,* , residuals), while the third and fourth columns report the within cell and location variance respectively. Parameter estimations use the model with asymmetry on v_- and v_+; the posterior median v_- and v_+ values are averaged over the two sisters. Stars denote that there is statistically significant difference ($\alpha < 0.01$) between groups of this factor. The ratio of the between cell to the between location variance is shown in fifth column and the size of effect (ratio of population parameters' standard deviation to the population parameters' (abs) mean) in last column. Stars denote the statistically different ratio at $\alpha < 0.01$. Caution: Due to low coverage of KT pairs per cell within each radius group, these results should be taken with caution as some of the ANOVA assumptions are violated.

	Symmetric		Asymmetric	
Parameter	Median	PIR	Median	PIR
α	0.012 [0.009]	0.005	0.016 [0.009]	0.005
κ	0.017 [0.017]	0.013	0.019 [0.024]	0.013
L	0.813 [0.042]	0.176	0.840 [0.077]	0.156
τ	830.6 [265.34]	86.42	824.1 [229.1]	83.77
v_-^1	-0.039 [0.020]	0.006	-0.053 [0.018]	0.009
v_-^2	-0.039 [0.020]	0.005	-0.030[0.021]	0.009
v_+^1	0.006 [0.007]	0.005	0.002 [0.002]	0.007
v_+^2	0.006 [0.07]	0.005	0.016 [0.009]	0.007
p_{icoh}	0.777 [0.220]	0.120	0.803 [0.226]	0.133
p_{coh}	0.947 [0.0208]	0.021	0.952 [0.019]	0.020

Table D. Summary statistics of symmetric and asymmetric sisters of nocodazole washout treated cells. We label sister 1 as the sister with the greater pulling force ,*i.e.,* $|v_-^1| \geq |v_-^2|$. The second and fourth columns reports the medians of posterior medians for symmetric and asymmetric sisters respectively, while brackets report the population interquartile range. The third and fifth columns report the median of Posterior Interquartile Ranges (PIR) for symmetric and asymmetric sisters, respectively. Based on Mann-Whitney and Kolmogorov-Smirnov tests, the all asymmetric posterior distributions but τ are significantly different that the symmetric posterior distributions, with size of effect (*i.e.,* , percentage difference of the medians) being 15.7%, 26.4% and 9.5%, 20.6%, 2.6% for $\kappa, \alpha, v_-, v_+, L$ respectively . Note that asymmetric parameter values on v_- and v_+ are averaged over the two sisters.

Preferred Model	Pearson	Kendall	Spearman
α	4.8×10^{-1}	1.2×10^{-1}	1.2×10^{-1}
κ	2.2×10^{-16}	2.2×10^{-16}	2.2×10^{-16}
L	9.8×10^{-1}	1.2×10^{-3}	1.2×10^{-3}
τ	1.9×10^{-15}	6.5×10^{-15}	9.4×10^{-15}
v_-	2.2×10^{-16}	2.2×10^{-16}	2.2×10^{-16}
v_+	7.8×10^{-7}	2.7×10^{-7}	2.5×10^{-7}

Table E. Spatial biophysical parameter trends across the metaphase plate: p-values of Pearson's, Kendall's and Spearman's tests for the presence of a trend, in 798 kinetochore pairs over 26 DMSO treated cells.

Preferred Model	Population	Cell
α	4.8×10^{-1}	6.5×10^{-1}
κ	2.2×10^{-16}	4.5×10^{-5}
L	9.8×10^{-1}	1.9×10^{-1}
τ	1.9×10^{-5}	2.3×10^{-15}
v_-	2.2×10^{-16}	2.2×10^{-5}
v_+	7.8×10^{-7}	7.5×10^{-1}

Table F. Spatial biophysical parameter trends across the metaphase plate for a single cell: p-values of Pearson's test for the presence of a trend, in 798 kinetochore pairs over 26 DMSO treated cells (column 2, as Table A5), and for 36 KT pairs in one DMSO treated cell (column 3).

Parameter	DMSO	Nocodazole washout	Relative Change
α	0.0131	0.0148	12.9%
κ	0.0277	0.0226	−18.4%
L	0.820	0.841	2.56%
τ	703	844	20.0%
v_-	-0.0446	-0.0420	−5.85%
v_+	0.0100	0.00625	−37.5%
p_{coh}	0.937	0.944	0.75%
p_{incoh}	0.741	0.766	3.37%

Table G. Comparison of (posterior) medians of $M^a_\pm$ model parameters between DMSO and nocodazole washout treated cells. The relative change (column 4) quantifies the percentage difference from DMSO to nocodazole washout treated cells; a negative relative change indicates a reduction under nocodazole washout treatment.

	**DMSO**		
**Cluster**	$r < 3.3\ \mu m$, $n = 418$	$r \geq 3.3\ \mu m$, $n = 418$	**Total** ($n = 836$)
strong/short	40 (9.6%)	30 (7.2%)	70 (8.4%)
strong/long	125 (29.9%)	64 (15.3%)	189 (22.6%)
weak/long	52 (12.4%)	36 (8.6%)	88 (10.5%)
weak/noisy	78 (18.7%)	112 (26.8%)	190 (22.7%)
poor	123 (29.4%)	176 (42.1%)	299 (35.8%)
	**Nocodazole washout**		
**Cluster**	$r < 3.3\ \mu m$, $n = 485$	$r \geq 3.3\ \mu m$, $n = 486$	**Total** ($n = 971$)
strong/short	69 (14.2%)	45 (9.3%)	114 (11.7%)
strong/long	147 (30.3%)	109 (22.5%)	256 (26.4%)
weak/long	46 (9.5%)	52 (10.7%)	98 (10.1%)
weak/noisy	96 (19.8%)	142 (29.3%)	238 (24.5%)
poor	128 (26.3%)	137 (28.2%)	265 (27.3%)

Table H. Oscillations deteriorate towards the MPP periphery. Percentages indicate the proportion of each oscillator type in the far ($r \geq 3.3 \mu m$) versus near ($r < 3.3 \mu m$) distance from the metaphase plate origin. There are significant differences in both DMSO treated cells ($p_{\text{Pearson}} = 5.48 \times 10^{-8}$) and nocodazole washout treated cells ($p_{\text{Pearson}} = 0.00044$).

**Variable (medians)**	***p*-value**	**BH-adjusted *p***
**DMSO**		
α	5.75×10^{-6}	$1.15 \times 10^{-5*}$
κ	7.70×10^{-5}	$1.23 \times 10^{-4*}$
L	1.79×10^{-2}	$2.05 \times 10^{-2*}$
τ	9.29×10^{-1}	9.29×10^{-1}
v_-	7.20×10^{-22}	$5.76 \times 10^{-21*}$
v_+	4.07×10^{-3}	$5.43 \times 10^{-3*}$
p_{coh}	1.80×10^{-6}	$4.80 \times 10^{-6*}$
p_{incoh}	1.25×10^{-13}	$5.00 \times 10^{-13*}$
**Nocodazole washout**		
α	6.62×10^{-11}	$2.65 \times 10^{-10*}$
κ	2.57×10^{-1}	2.94×10^{-1}
L	8.11×10^{-1}	8.11×10^{-1}
τ	6.70×10^{-2}	1.07×10^{-1}
v_-	1.22×10^{-15}	$9.76 \times 10^{-15^*}$
v_+	7.34×10^{-2}	9.79×10^{-2}
p_{coh}	3.94×10^{-5}	$7.88 \times 10^{-5*}$
p_{incoh}	9.39×10^{-9}	$2.51 \times 10^{-8*}$

Table I. Strong and poor oscillators have distinctly different biophysical parameters. Hypothesis tests for $M^a_{v\pm}$ model parameters comparing strong and poor/not oscillators using the Mann-Whitney test. We used $n = 257$ (strong oscillators) and $n = 359$ (poor/not oscillators) KT-pair estimates for DMSO-treated cells, and $n = 294$ (strong oscillators) and $n = 244$ (poor/not oscillators) for nocodazole washout treated cells. P-values are adjusted using Benjamini-Hochberg to control for 16 multiple comparisons (8 variables $\times$ 2 conditions). Statistically significant tests ($p < 0.05$) are marked with $*$.

Radius Interval (μm)	n_{strong}	n_{poor}	p-value	p-value (adj.)
**DMSO**				
$[0.40, 1.99]$	65	46	0.02	0.04^*
$[2.02, 2.86]$	63	47	0.13	0.13
$[2.90, 3.58]$	53	57	1.56×10^{-4}	$7.81 \times 10^{-4*}$
$[3.58, 4.00]$	41	69	3.00×10^{-3}	$7.51 \times 10^{-3*}$
$[4.00, 4.99]$	35	75	0.04	0.05^*
**Nocodazole washout**				
$[0.32, 2.06]$	77	44	0.09	0.11
$[2.06, 2.87]$	76	45	0.18	0.18
$[2.87, 3.50]$	79	42	3.20×10^{-3}	$7.99 \times 10^{-3*}$
$[3.51, 4.01]$	67	53	0.01	0.02^*
$[4.02, 4.92]$	60	60	2.48×10^{-3}	$7.99 \times 10^{-3*}$

Table J. Strong oscillators are more prevalent towards the centre of the metaphase plate, and have a significantly longer directional switching time. Comparison of p_{incoh} distributions between strong and poor oscillators across different radial positions from the metaphase plate intervals in DMSO and Nocodazole conditions. The radius intervals represent quintiles of average radius; n_{strong} and n_{poor} indicate the number of observations in each group. p-values are from Wilcoxon tests comparing distributions of p_{incoh} between strong and poor oscillators within each radius group, with adjusted p-values controlling for multiple testing.

Oscillators	$n_{\mathbf{total}}$	$n_{\mathbf{reversals}}$	Proportion of Reversals
Strong/Short	164	70	0.427
Strong/Long	399	155	0.388
Weak/Long	167	50	0.299
Weak/Noisy	371	122	0.329
Poor	496	135	0.272

Table K. Trajectories with reversal events are more frequent in strong oscillators. This table summarises the number of pairs in each oscillator type cluster (in the pulled dataset), the number of trajectories with reversal events per oscillator type and the relevant proportion. One-tailed two-proportion z-tests for reversal proportions showed that the reversal proportions are higher in strong oscillators. Note that this is obvious when comparing (a) Strong (Short + Long) vs Weak (Noisy + Long): $p_{Ztest} = 0.0029$ and (b) Strong (Short + Long) vs Weak + Poor: $p_{Ztest} = 1.6 \times 10^{-5}$ oscillators. Both tests show significantly higher proportions of reversals in Strong oscillators.

Parameter	Median (No reversals)	Median (Reversals)	p-value
	**DMSO**		
α	0.0109	0.0109	0.8274
κ	0.0121	0.0138	0.1356
L	0.8027	0.7953	0.0147*
τ	567.29	558.55	0.2693
v_-	0.0372	0.0421	0.0011*
v_+	0.0089	0.0101	0.0045*
p_{coh}	0.9565	0.9569	0.2887
p_{incoh}	0.7414	0.7677	0.2710
	**Nocodazole**		
α	0.0131	0.0134	0.2939
κ	0.0102	0.0160	$5.1 \times 10^{-6*}$
L	0.8081	0.8146	0.0037*
τ	707.93	663.50	$6.0 \times 10^{-4*}$
v_-	0.0357	0.0420	$1.7 \times 10^{-9*}$
v_+	0.0057	0.0056	0.5134
p_{coh}	0.9564	0.9519	0.0052*
p_{incoh}	0.7561	0.8311	$8.3 \times 10^{-10*}$

Table L. Summary of results comparing parameter distributions between of the trajectories without reversal events in the anaphase and with reversal events in DMSO and nocodazole washout treated cells. Medians are reported for each group, and p-values (Mann-Whitney tests) less than 0.05 are marked with *.

B Additional plots

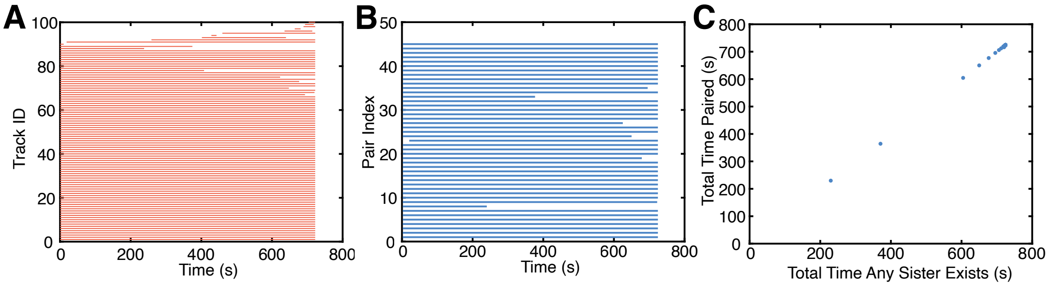

Fig A. Near-complete tracking of kinetochores through metaphase and anaphase in a human RPE1 cell (tracks are shown in Fig 1 of the main text). A Tracklet plot showing detected KT tracks, tracing the track through time; 82 tracks run through the entire movie, (3 missing time points are allowed, not necessarily consecutive). Because of track breakage, an individual kinetochore could be tracked more than once, resulting in more than a total of 92 tracks. **B** Pairlet plot showing the times in a movie that a sister pair (by pair index) exists (and is paired). **C** Total time a sister pair is paired against total time either sister exists.

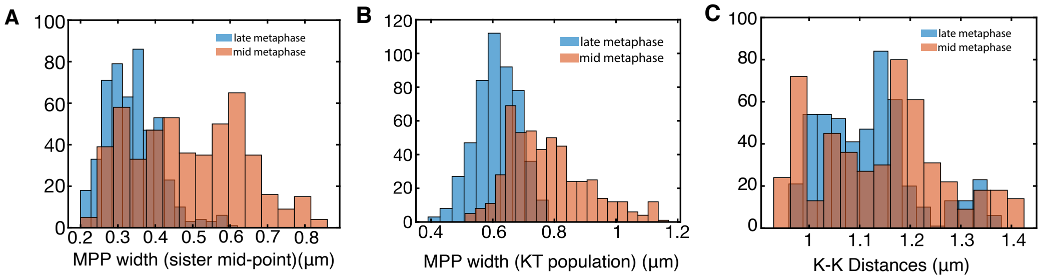

Fig B. Quantification of intrametaphase maturation. Comparison of mid- (orange) and late-metaphase (blue) measured at 330-230s, respectively 130-30s, before anaphase onset. **A** MPP width as measured by paired sister mid-point width (smallest eigenvalue of the covariance matrix of kinetochore mid-points). **B** Metaphase plate (MPP) width as measured by the covariance matrix of the KT population (smallest eigenvalue). **C** Kinetochore-Kinetochore (KK) distance. Data include only cells with at least 30 sisters both tracked for 75% of movie. Mid and late metaphase had significantly different MPP width (both measures, **A**, **B**), and KK distance ($p_{MW} < 10^{-3}$).

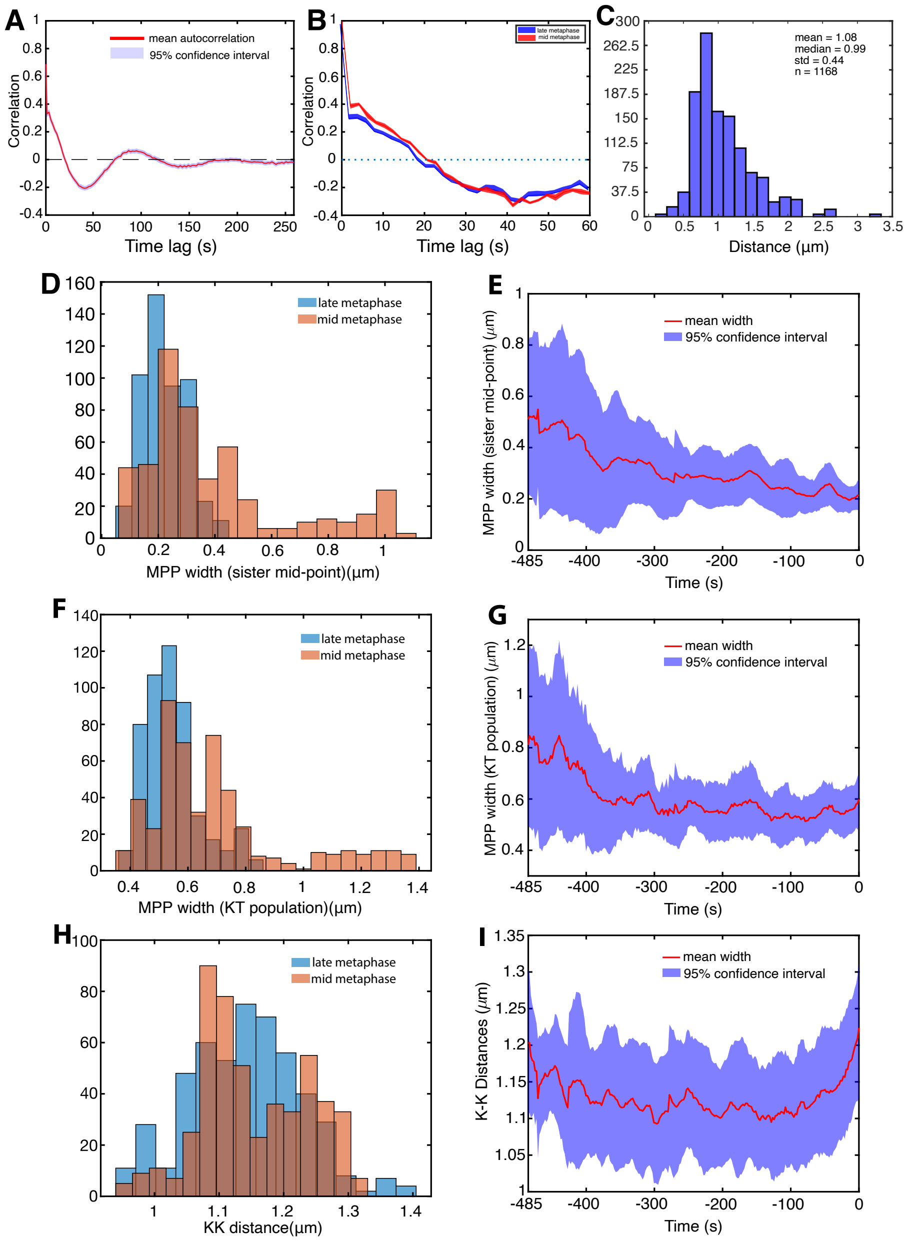

Fig C. Quantification of intrametaphase maturation in nocodazole washout treated cells. **A** Autocorrelation plot showing the temporal correlation of metaphase oscillations. **B** Autocorrelation of metaphase oscillations in late (red) and mid (blue) metaphase. **C** Sister kinetochore (KK) distance pooled over all KT pairs and time. **D/E** MPP width as measured by paired sister mid-point width (smallest eigenvalue of the covariance matrix of kinetochore mid-points). MPP statistics: **F/G** Metaphase plate (MPP) width as measured by the covariance matrix of the KT population (smallest eigenvalue). **H/I** KK distance. Comparison of mid (orange) and late (blue) metaphase in **D,F,H**, and time-series (time before anaphase onset) in **E,G,I**, mean (red) and standard deviation (blue). Data are based on cells having at least 30 sisters both tracked for 75% of the movie (31 cells in total). Early and mid-metaphase median estimates of MPP in D and F where significantly different (minimum $p_{MW} < 10^{-30}$, while the KK-distance in early an mid-metaphase remained the same, $p_{MW} = 0.026$.

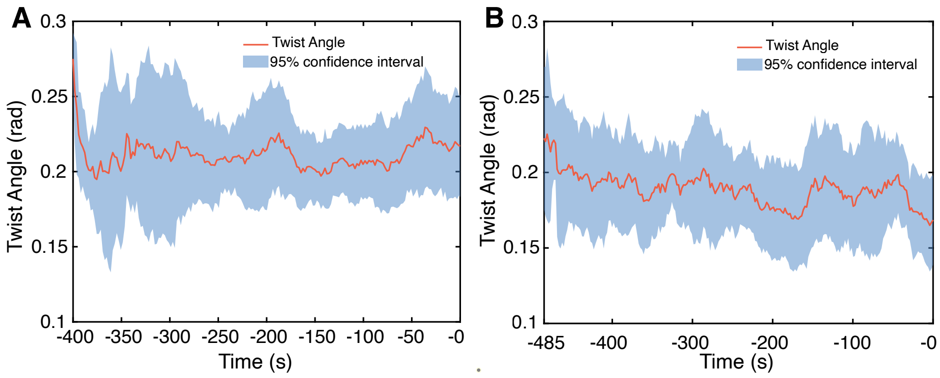

Fig D. Mean twist angle (in radians) in **A** DMSO **B** nocodazole washout treated data. Shaded blue areas denote the 95% confidence intervals. Time 0 denotes the anaphase onset.

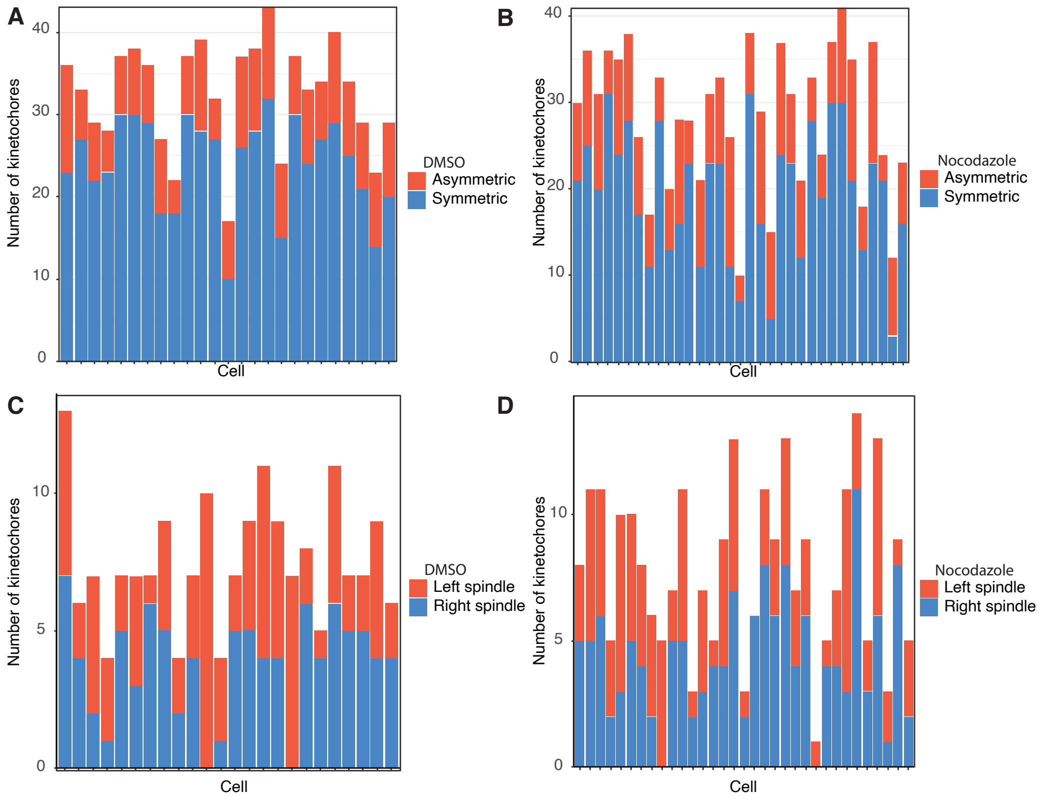

Fig E. All cells present a significant fraction of asymmetric sisters and no half-spindle bias is observed. **A**/**B** Symmetric (blue) and significantly asymmetric (red) sister pairs within a cell in **A** DMSO and **B** nocodazole washout treated cells. **C**/**D** Number of stronger pulling asymmetric sisters positioned in the left (blue) and right (red) half-spindle for **C** DMSO and **D** nocodazole washout treated cells. Stronger pulling asymmetric sisters proportions are not statistically different to $p = 0.5$ in left and right spindle with $p_{Binom} = 0.88$ for DMSO and $p_{Binom} = 0.10$ for nocodazole washout treated cells (Binomial test).

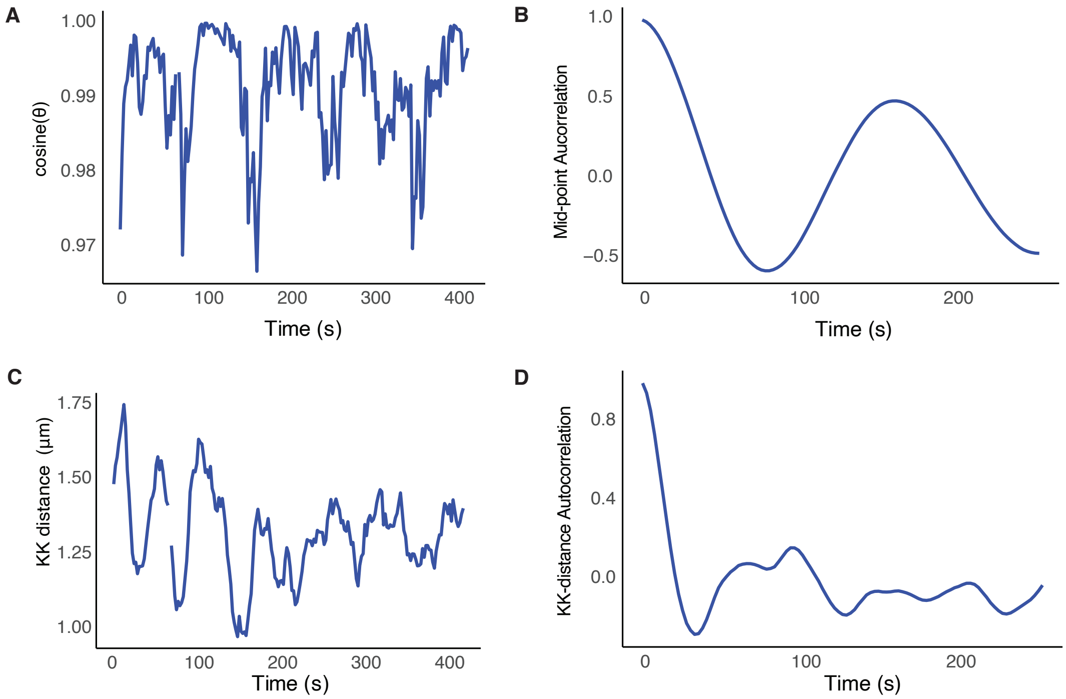

Fig F. Additional figures for the KT-pair of Fig 4A of the main text. **A** Time series of $cosine(\theta_t)$, where θ_t is the angle between the normal to the MPP and the sister to sister vector at time t. **B** ACF of sister pair midpoint, confirming that the pair is a good oscillator. **C** Intersister (KK) distance time series. **D** ACF of KK distance (breathing oscillation)

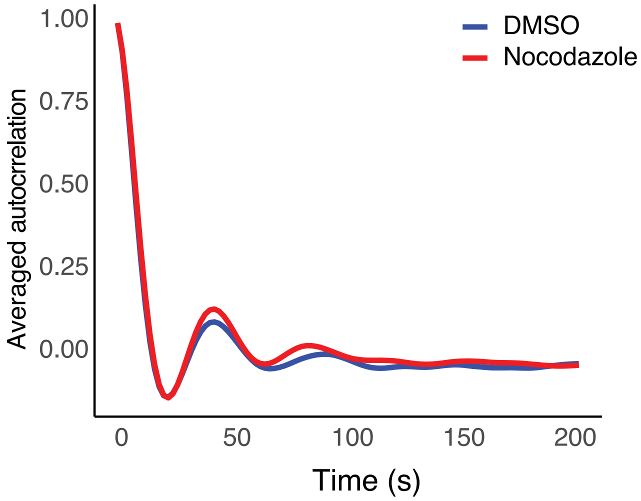

Fig G. Autocorrelation function of Kinetochore to Kinetochore (KK) sister distance (breathing oscillation): averaged ACF of the distance between sister kinetochores, *i.e.,* the Kinetochore to Kinetochore (KK) distance for DMSO (blue) and nocodazole washout (red) treated cells. Data based on 26 DMSO cells, 33 nocodazole washout cells.

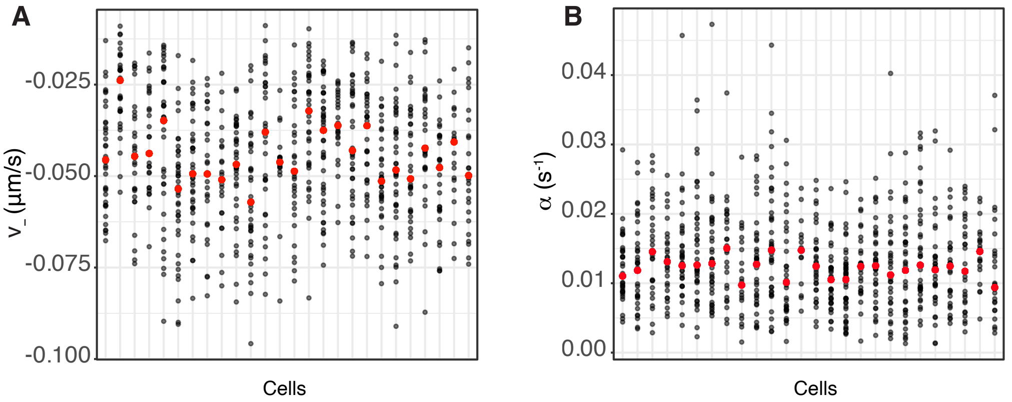

Fig H. Posterior median estimations of **A** v_- and **B** α shown with respect the 26 DMSO treated cells (x-axis). Posterior medians are plotted for each KT pair (grey dot).Red dots are the median (of plotted data) per cell. Between cell variation is evident in both plots.

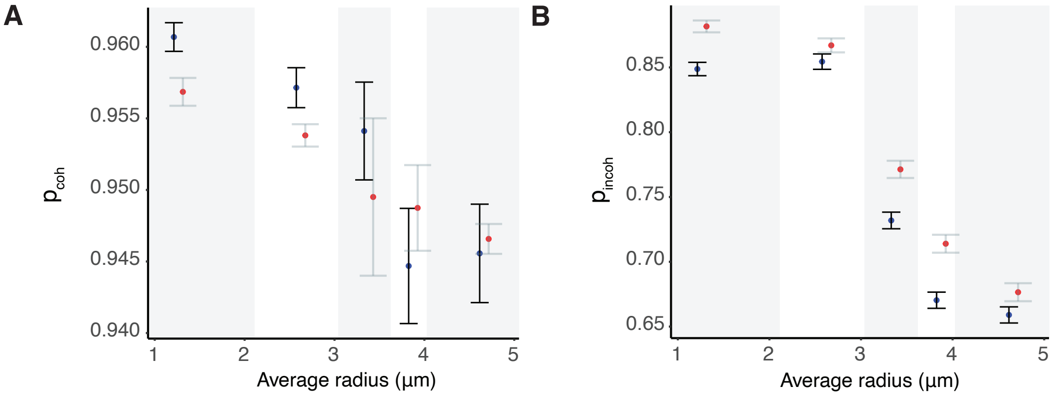

Fig I. Trends in KT pair directional switching control across the metaphase plate for DMSO (blue) and nocodazole washout (red) treated cells, (complimenting Fig 10 of the main text). Groups are equisized (containing the same number of KT pairs), with the five groups defined by the range of the average radial distance: $[0, 2.03], (2.03, 2.95], (2.95, 3.58], (3.580, 4.01], (4.01, 5.03]$. Posterior median and standard deviation are plotted for each partition. **A** Probability of remaining coherent per frame (states $+-$ or $-+$), p_{coh}. **B** Probability of remaining incoherent (states $++$ or $--$), p_{incoh}. Data from 798 and 984 KT pairs, DMSO, nocodazole washout treatment respectively, over 59 cells.

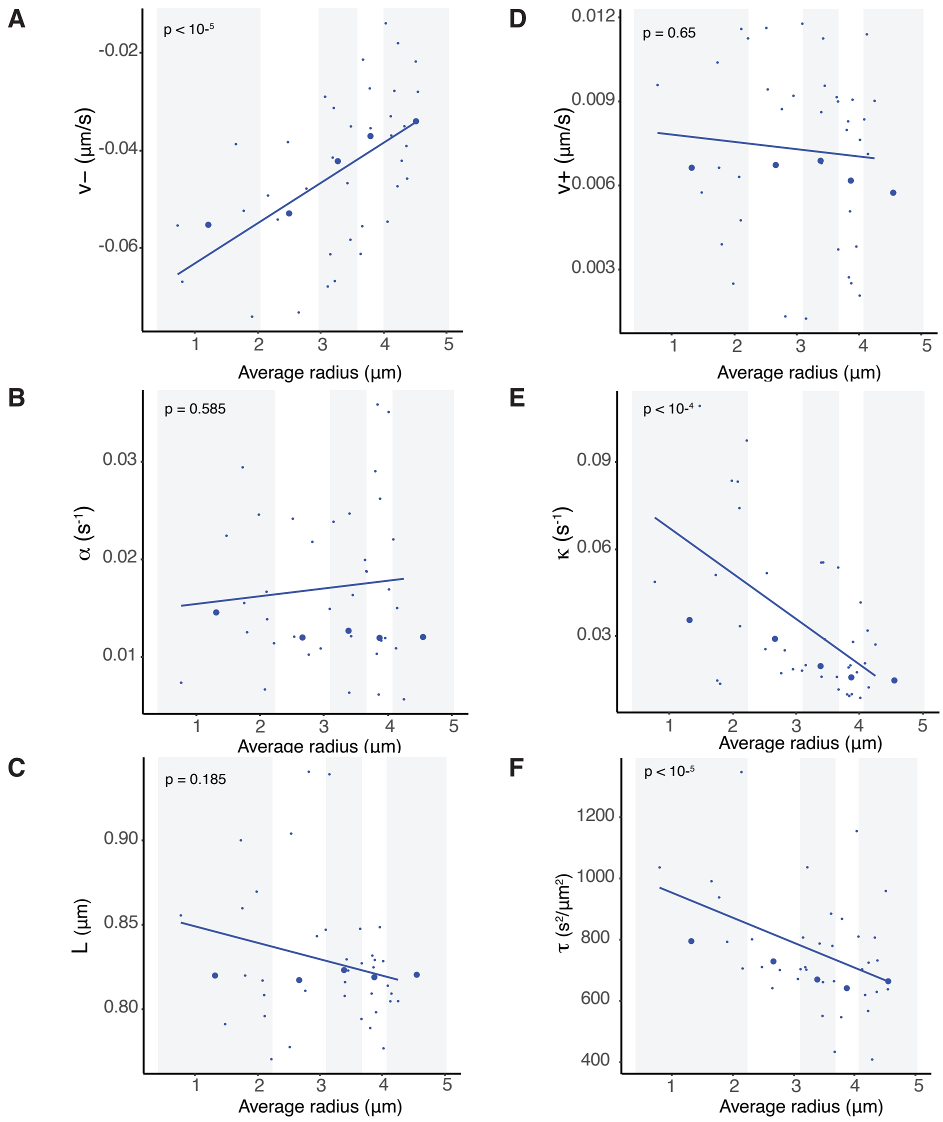

Fig J. Spatial biophysical parameter trends across the metaphase plate for a single (DMSO) cell. Trends are similar to that observed in pooled cell data, Fig. 7 of main text. Data partitioned into 5 groups for the average radial KT-pair distance within the metaphase plate as in Fig. 7, *i.e.*, , $[0, 2.03], (2.03, 2.95], (2.95, 3.58], (3.580, 4.01], (4.01, 5.03]$. Small dots correspond to a KT pair measurement (posterior median) and large dots median of the group. Regression line in blue. **A** Pulling forces v_-, **B** Pushing forces v_+, **C** Spring constant κ, **D** Centralising forces, **E** precision τ, **F** Natural length of the centromeric chromatin spring connecting the kinetochore sisters L.

Maturation during metaphase

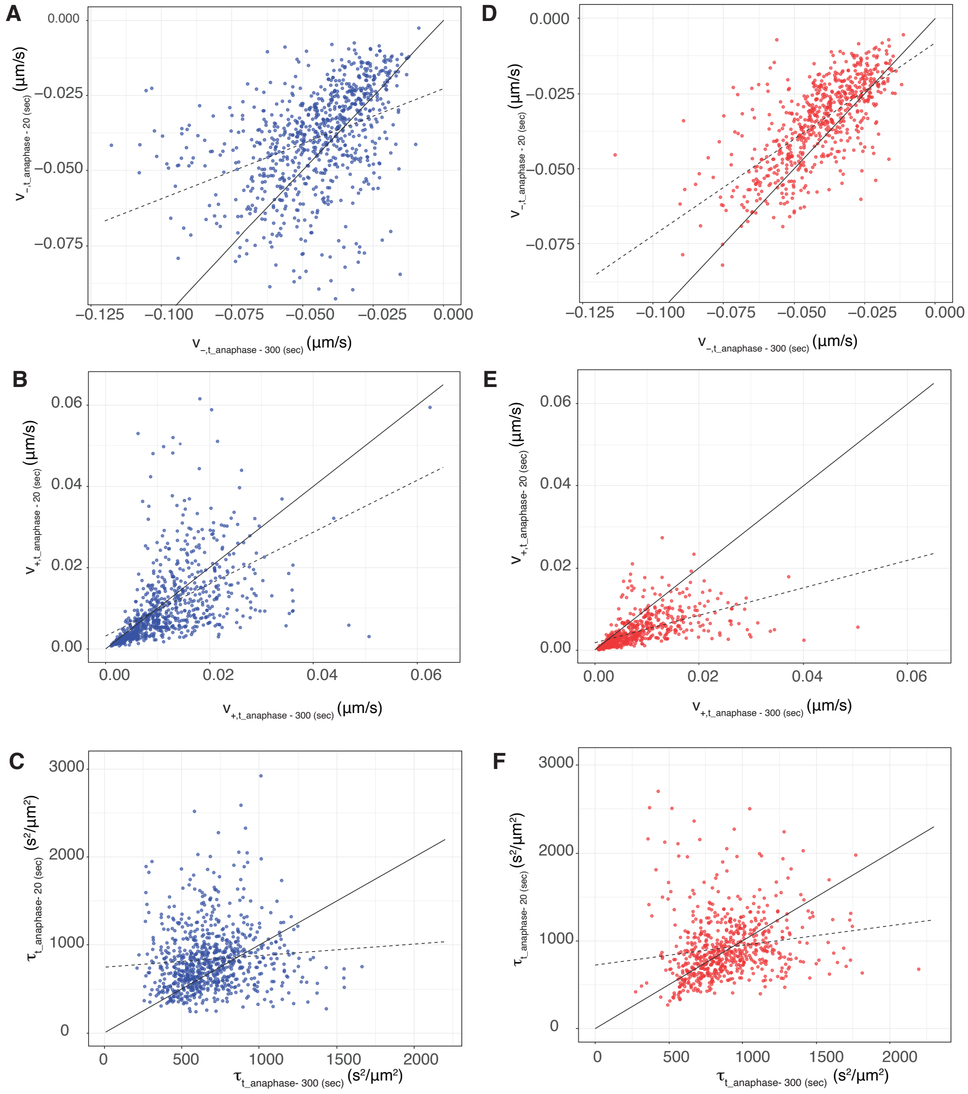

Fig K. Maturation towards an anaphase ready state. Temporal changes of speed and noise across the last 5 mins of metaphase. Estimated parameters of the model with time dependent parameters v_-, v_+ and τ for **A**/**B**/**C** DMSO (blue) **D**/**E**/**F** nocodazole washout (red) treated cells, respectively. Estimations are based on the median posterior parameters and calculated at two different time points at 300 seconds and 30 seconds prior to anaphase, *i.e.,* $t = t_{anaphase} - 20$ (y-axis) and $t = t_{anaphase} - 300$ (x-axis). The discrete map comprising the line of regression and the 1:1 diagonal indicate the existence of a stable fixed point at $v_- = -0.04$ ($v_- = -0.02$), $v_+ = 0.01$ ($v_+ = 0.003$) and $\tau = 849$ ($\tau = 923$) in DMSO (nocodazole washout) treated cells.

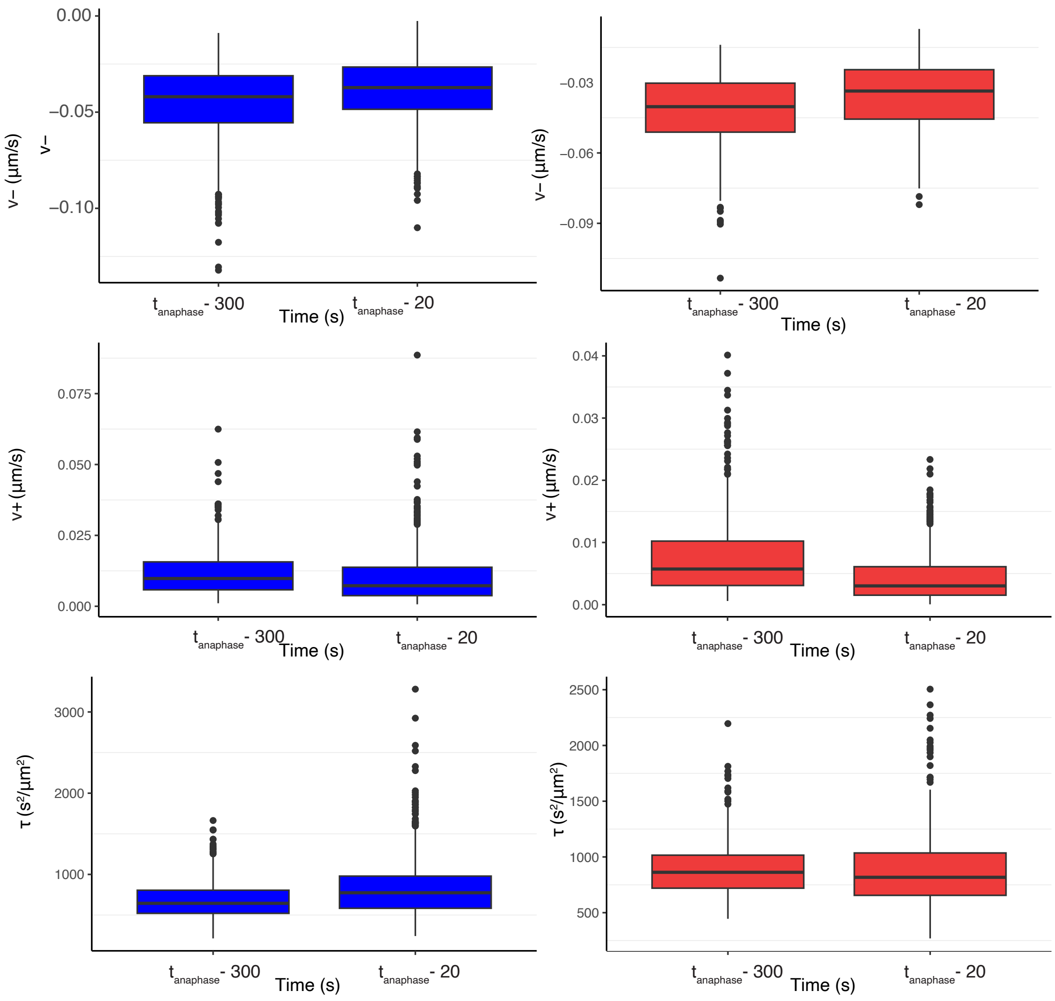

Fig L. Maturation of dynamic parameters from mid-metaphase (300 s before anaphase) and just before anaphase onset (20s before anaphase). Box plots of the posterior estimation of $v_-(t) = v_{-0} \exp\{v_{-1}t\}, v_+(t) = v_{+0} \exp\{v_{+1}t\}, \tau(t) = \tau_0 \exp\{\tau_1 t\}$, evaluated at time t set to 300 seconds and 20 seconds prior to anaphase, *i.e.,* $t = t_{anaphase} - 300$ and $t = t_{anaphase} - 20$. DMSO (blue) and nocodazole washout (red) treated cells.

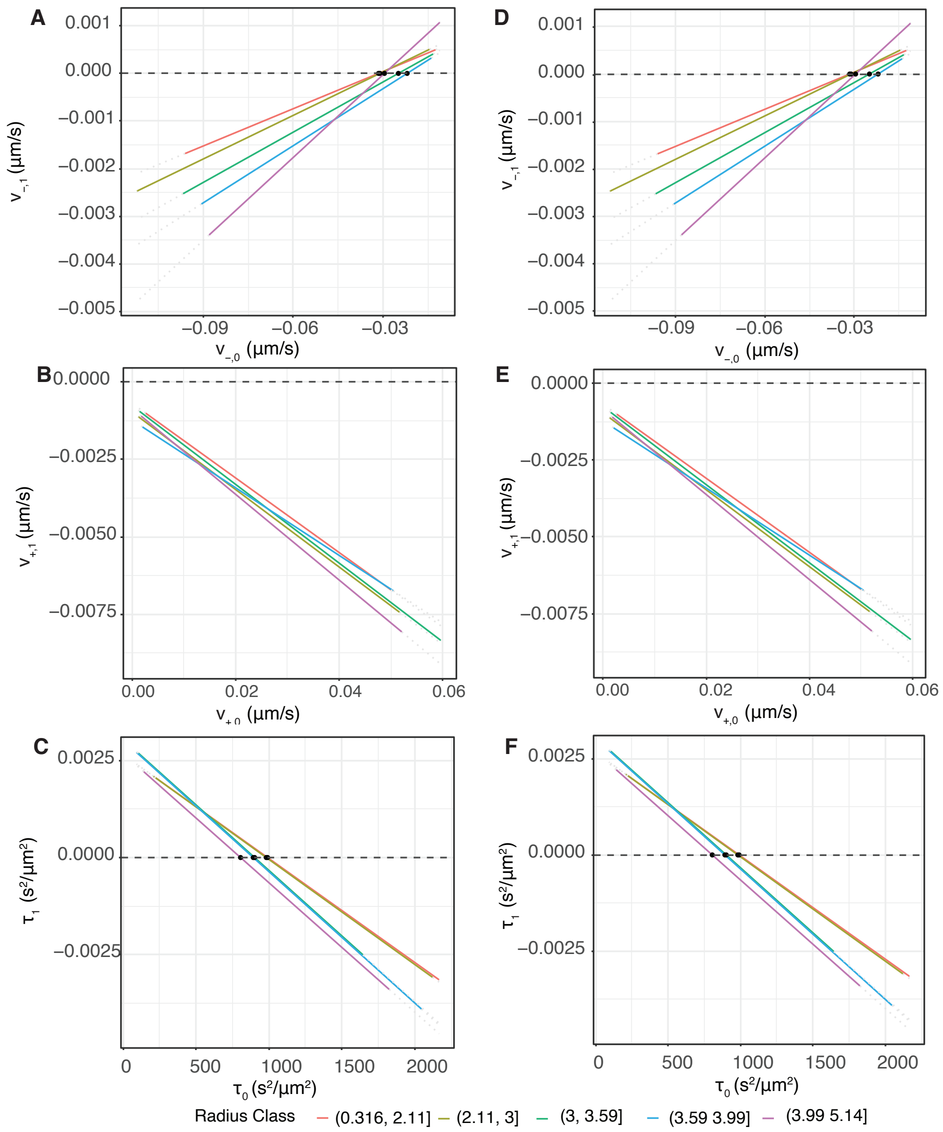

Fig M. Tuning is consistent across the MPP. Plots of the regression lines for the KT pairs belonging in each of the 5 radius classes (with the five groups defined by their radial distances, *i.e.,* $[0, 2.11]$, $(2.113]$, $(3, 3.59]$, $(3.59, 3.99]$, $(3.99, 5.14]$ respectively) estimates of initial p_0 with time dependent parameter p_1. **A/D** Pulling forces v_- in DMSO, nocodazole washout treated cells, respectively. **B/E** Pushing forces v_+ in DMSO, nocodazole washout treated cells, respectively.**C/F** Noise τ in DMSO, nocodazole washout treated cells, respectively.

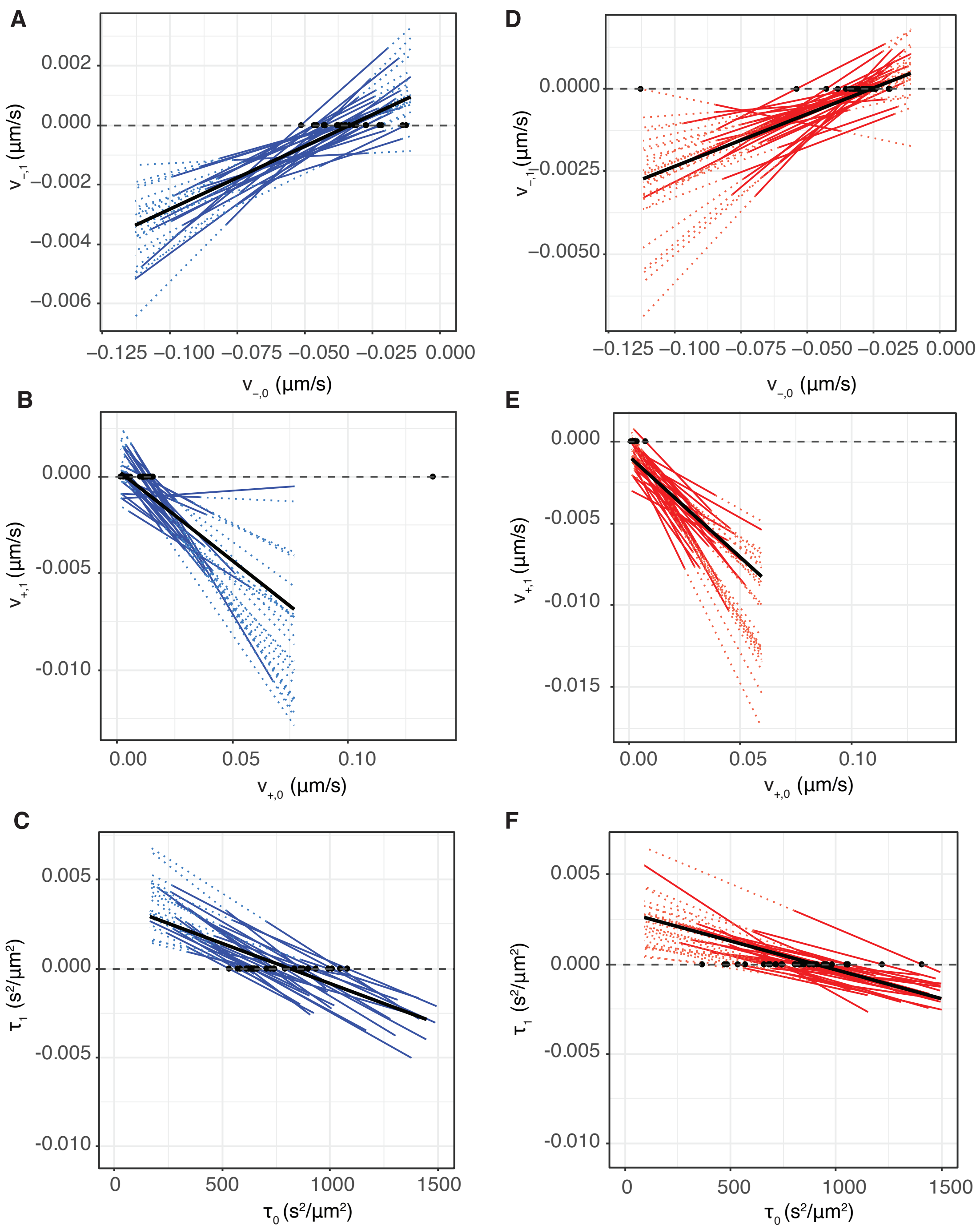

Fig N. Tuning occurs in individual cells. Plots of the regression lines for individual cells of KT pair estimates of initial p_0 with time dependent parameter p_1. **A/D** Pulling forces v_- in DMSO, nocodazole washout treated cells. 92.3% and 83% of cells have physical (negative) anaphase ready state estimates (intercept of regression line with $v_{-1} = 0$, **B/E** pushing forces v_+ in DMSO, nocodazole washout treated cells, 73.1% and 67.7% of cells have physical (positive) anaphase ready state, **C/F** precision τ, in DMSO, nocodazole washout treated cells,respectively, both datasets have 100% positive intercept at $\tau_1 = 0$.

Nocodazole washout treated cells: Asymmetry and maturation during metaphase

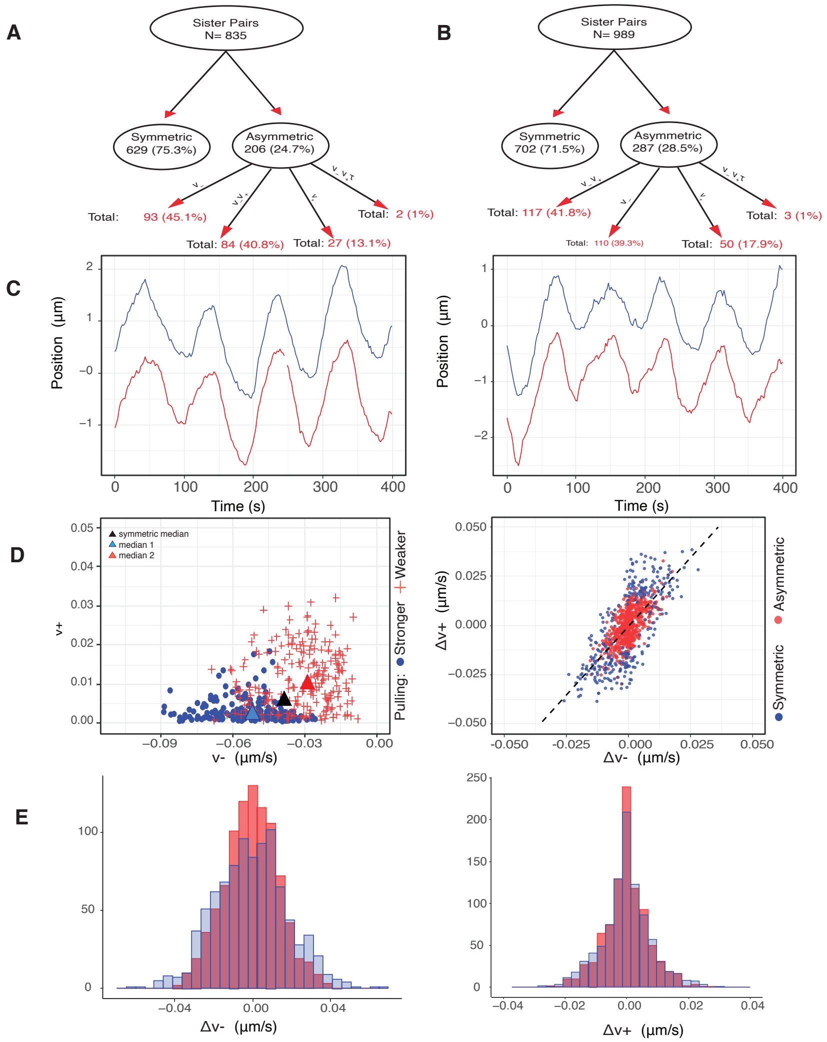

Fig O. Asymmetry in sister kinetochores is unaffected by nocodazole washout treatment. **A/B** Graphical representation of asymmetry model preference network in DMSO (A) and nocodazole washout (B) treated cells. **C** Typical symmetric (left) and asymmetric (right) trajectory. No distinctive characteristics between the symmetric and asymmetric sister pairs, nocodazole washout treated. **D** (LEFT) Comparison of inferred $v-$ and $v+$, using the asymmetric on v_-, v_+ model, over the nocodazole washout asymmetric pairs. Distinction of sisters within a pair is based on their pulling force strength. Stronger and weaker pulling sisters are denoted with blue and red. Population medians are marked with triangle, while black triangle shows the estimated population median of the symmetric pairs.(RIGHT) Direct comparison of $\Delta\left(v_-^1, v_-^2\right)$ versus $\Delta\left(v_+^1, v_+^2\right)$ of symmetric and asymmetric nocodazole washout treated sister pairs, in red and blue respectively, when inferring them with the v_-, v_+ model. Asymmetric pairs do not present any distinctive pattern. **E** Histograms of the nocodazole washout sister difference distribution, *i.e.*, $\Delta\left(v_-^1, v_-^2\right)$ (left) and of $\Delta\left(v_+^1, v_+^2\right)$ (right), when accounting for the sister pairing (red) and disturbing randomizing the pairing (blue), while accounting for the spatial metaphase plate distance.

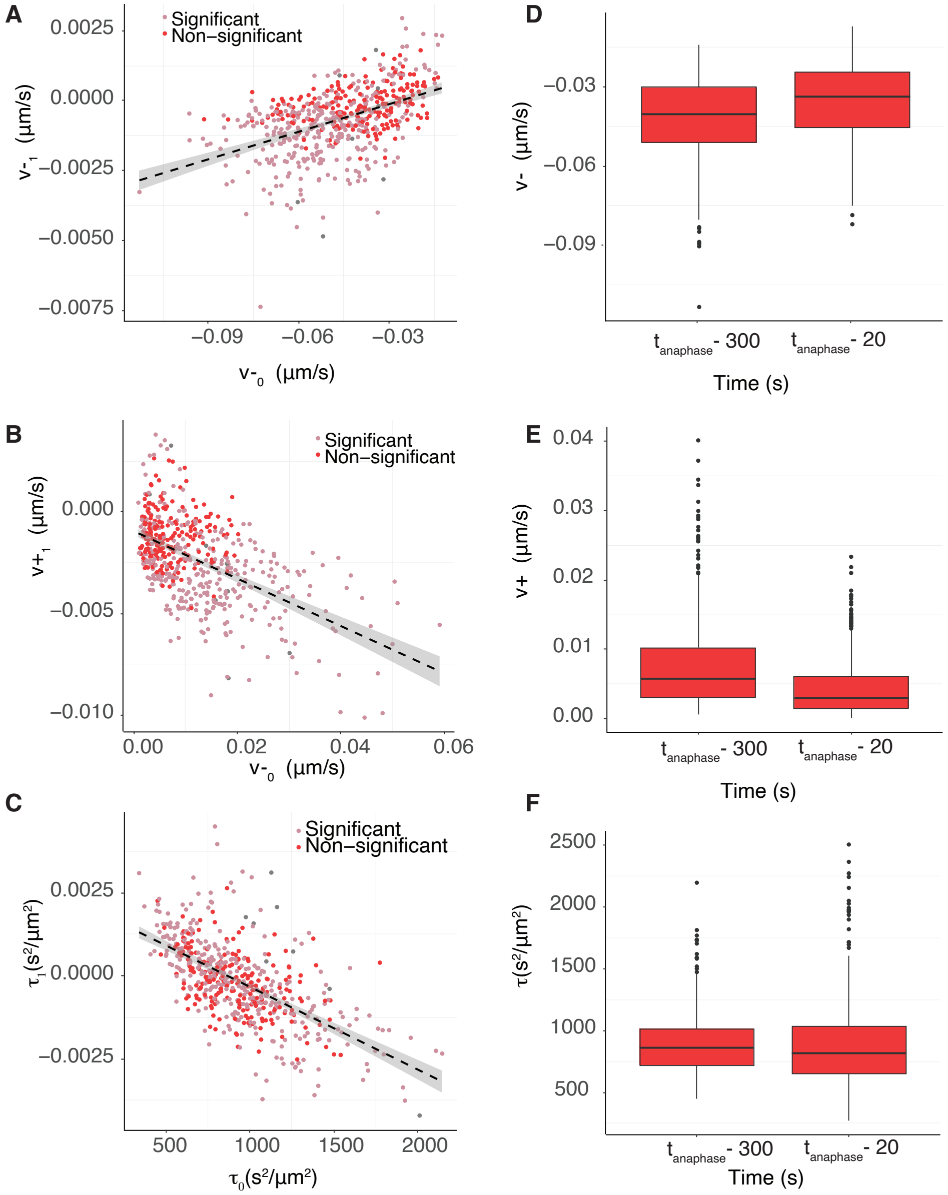

Fig P. Mechanical tuning in nocodazole washout treated cells. **A/B/C** Scatter plot of posterior median of time dependence p_1 versus initial parameter value p_0 for **A** pushing forces, $v_{1,+}$ versus $v_{0,+}$; **B** pulling forces, $v_{1,-}$ versus $v_{0,-}$; **C** $\tau_{1,+}$ versus $\tau_{0,+}$. **D/E/F** Boxplots of posterior median parameter at the beginning and the end of the trajectory for **D** pushing forces v_+; **E** pulling forces v_-; and **F** noise τ. Parameters v_-, v_+ are statistically different over time (mid-metaphase to late-metaphase comparison), ($p_{MW} < 10^{-16}, p_{MW} < 10^{-9}, p_{MW} < 10^{-16}$ for v_-, v_+, respectively), while τ is not found to be statistically different over time. Finally, variances over time are statistically different with ($p_{BF} < 10^{-17}, p_{BF} < 10^{-35}, p_{BF} < 10^{-3}$ for v_-, v_+, τ respectively). Parameters are inferred on the $M_{v\pm\tau}$ model.

KT positioning analysis

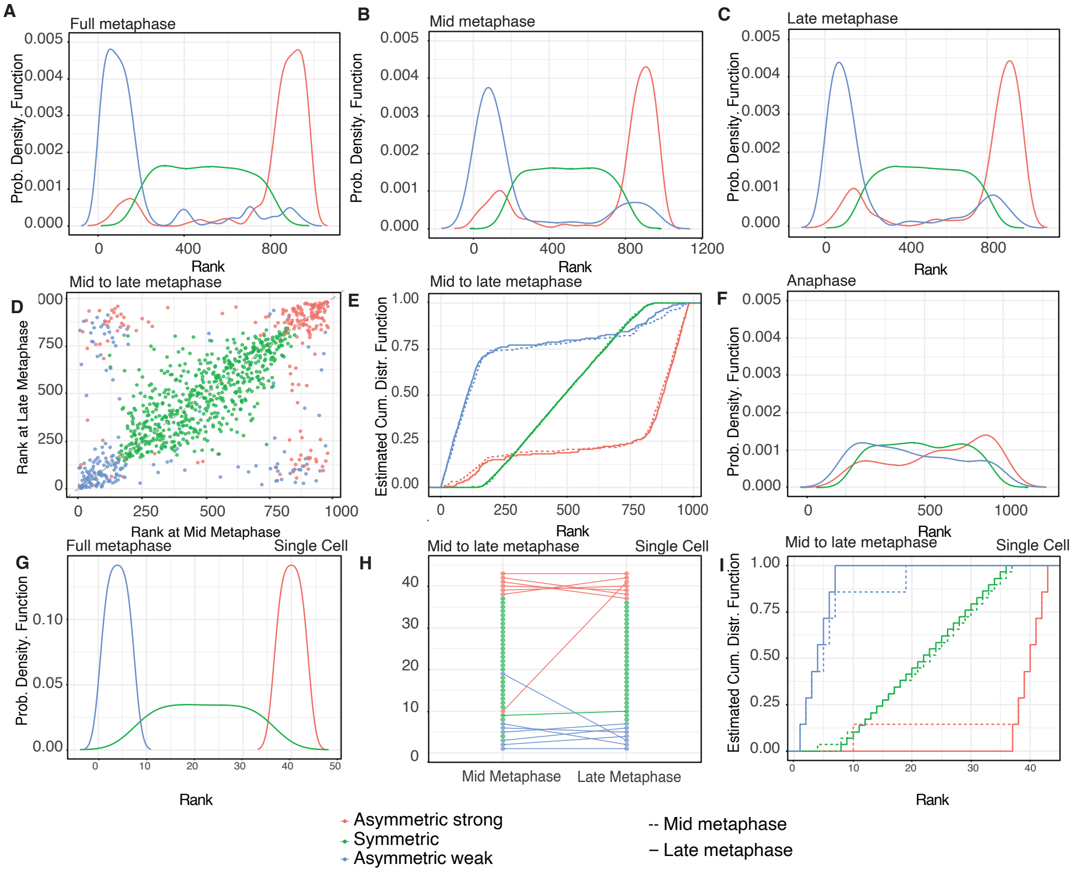

Fig Q. A rank analysis of the lateral organisation of the MPP in DMSO cultured cells. **A/B/C/F** Estimated probability density function of the ranks of averaged positions (from the centre of the metaphase plate) of DMSO treated kinetochores over different periods. **A** full metaphase (185 frames), **B** mid metaphase (80 frames), **C** late metaphase (80 frames) and **F** anaphase (80 frames). **D/E** Change of rank of the averaged positions from mid to late metaphase, for DMSO treated cells, averaged over 50 frames. **D** Scatter plot, **E** estimated cumulative distribution. **G** Estimated probability density function of the ranks of the averaged positions (from the centre of the metaphase plate) of kinetochores over the whole metaphase (185 frames) for a single cell. **H** Rank changes of the positions of kinetochores in a cell, from mid to late metaphase. Lines connecting points from mid to late metaphase indicate the rank of a particular sister KT at these two periods, for the same cell as in **G**. **I** Estimated cumulative distribution of rank changes from mid to late metaphase for the same cell as in G. Red colour denotes the stronger pulling asymmetric sisters, blue the weaker pulling asymmetric sisters and green the symmetric sisters.

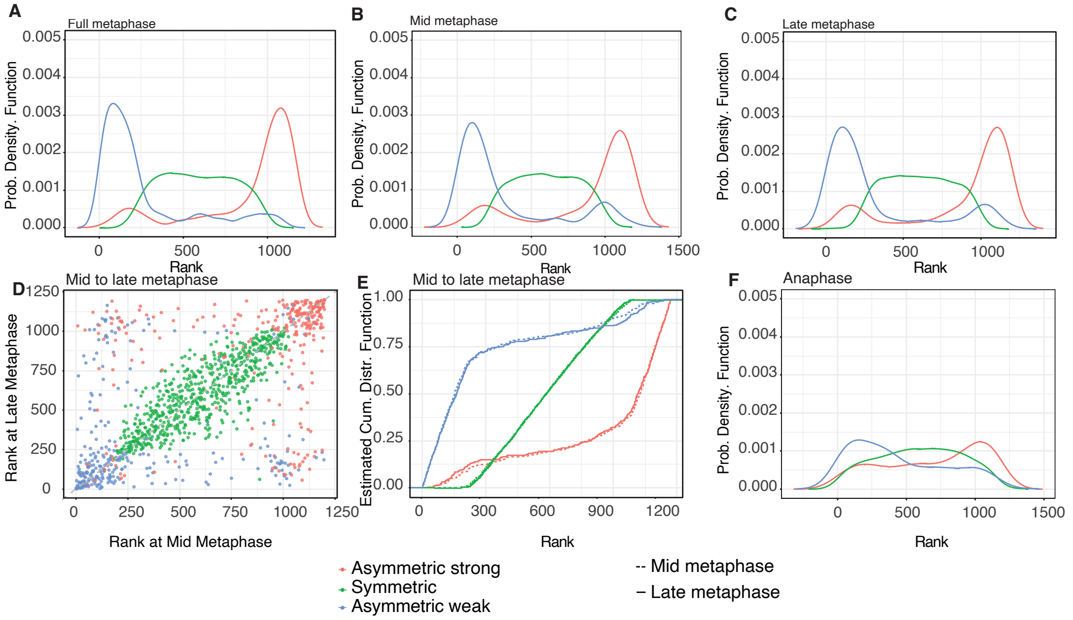

Fig R. A rank analysis of the lateral organisation of the MPP in nocodazole washout treated cells. **A**/**B**/**C**/**F** Estimated probability density function of the ranks of averaged positions (from the centre of the metaphase plate) of nocodazole washout kinetochores over different periods. **A** full metaphase (185 frames), **B** mid metaphase (80 frames), **C** late metaphase (80 frames) and **F** anaphase (80 frames). **D**/**E** Change of rank of the averaged positions from mid to late metaphase, averaged over 50 frames. **D** Scatter plot, **E** estimated cumulative distribution. Red colour denotes the stronger pulling asymmetric sisters, blue the weaker pulling asymmetric sisters and green the symmetric sisters.

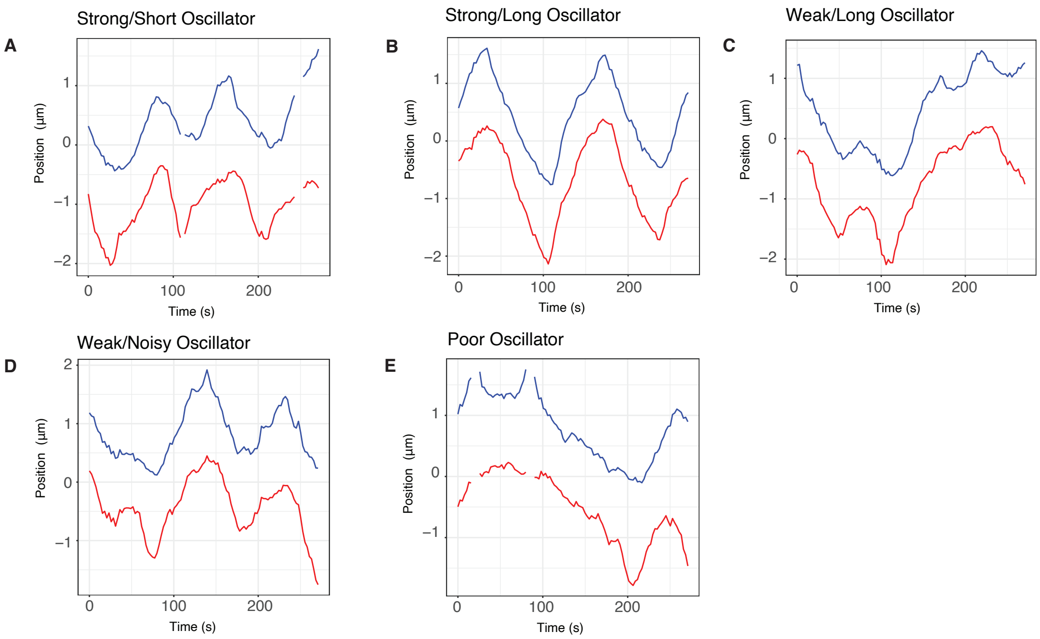

Fig S. Examples of KT pair trajectories for each of the five oscillator categories: strong oscillators with short period, strong oscillators with long period, weak oscillators with long period, weak and noisy oscillators and poor oscillators/non-oscillating KT pairs.

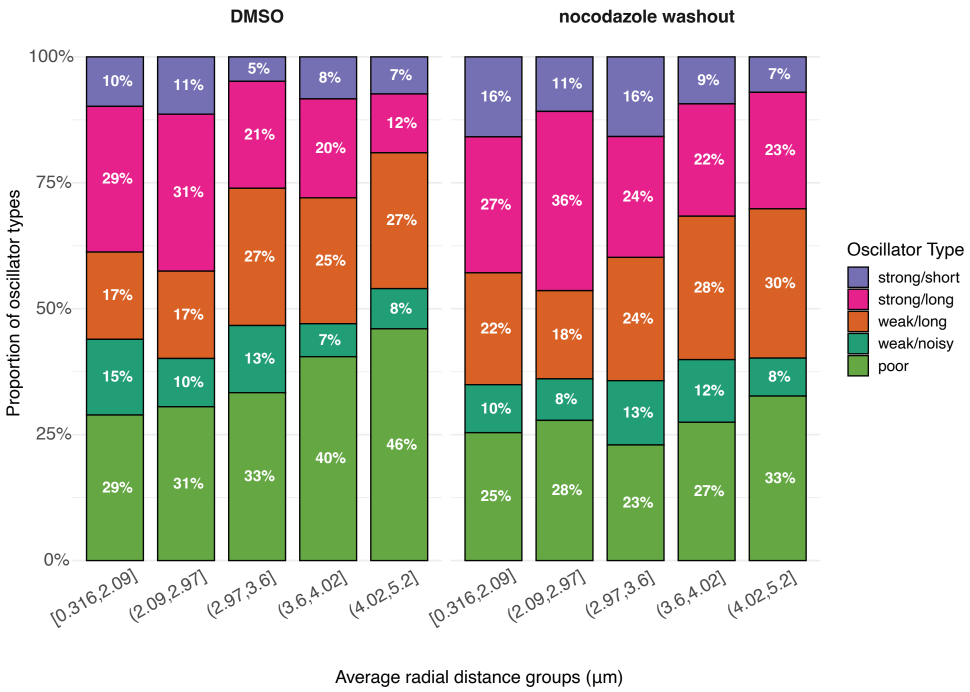

Fig T. Oscillator type profile shifts from strong to poor with distance from the centre of the MPP. Proportion of KT pairs in the 5 oscillator clusters for the shown radial partitions.

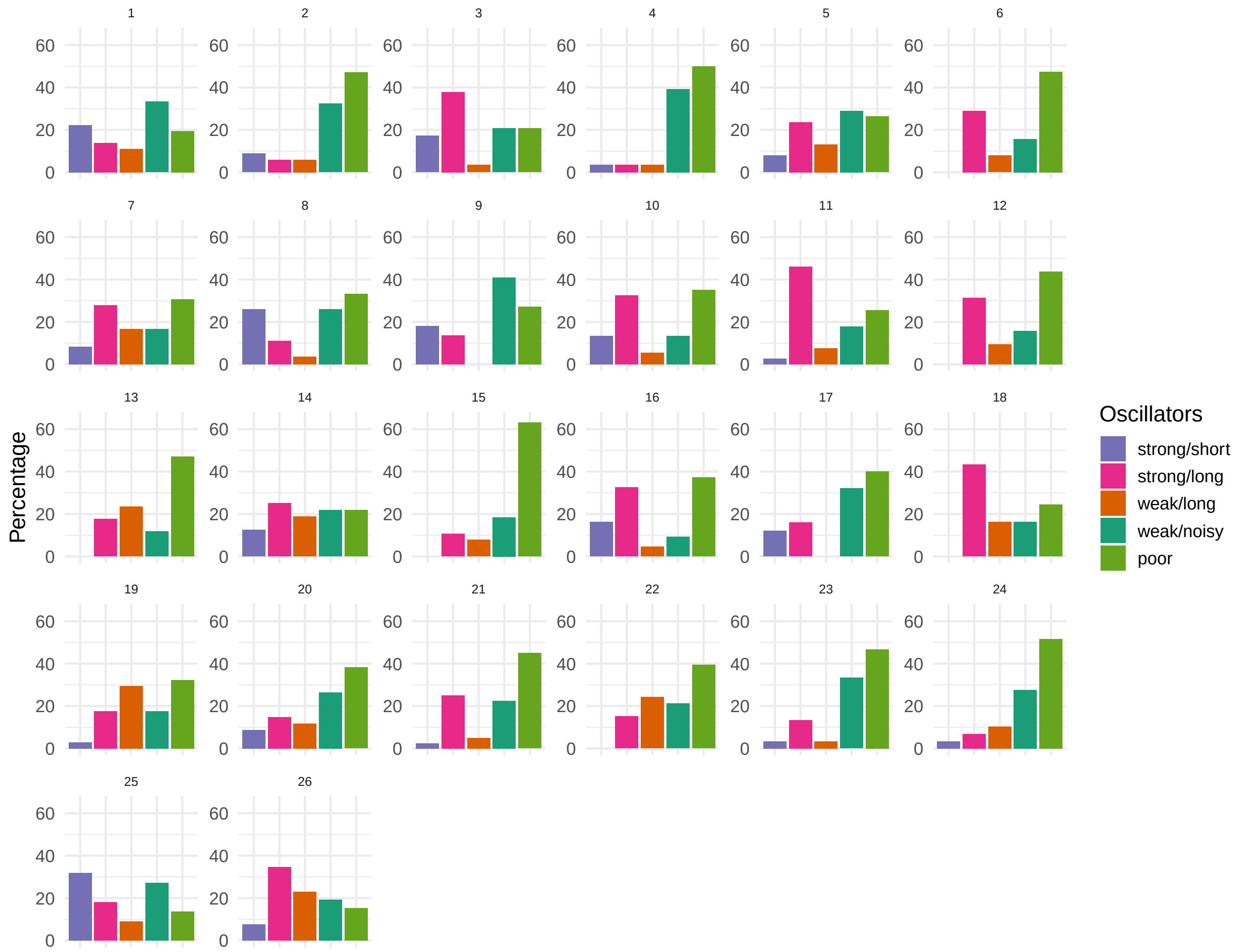

Fig U. Different oscillator types across each of the 26 DMSO treated cells.

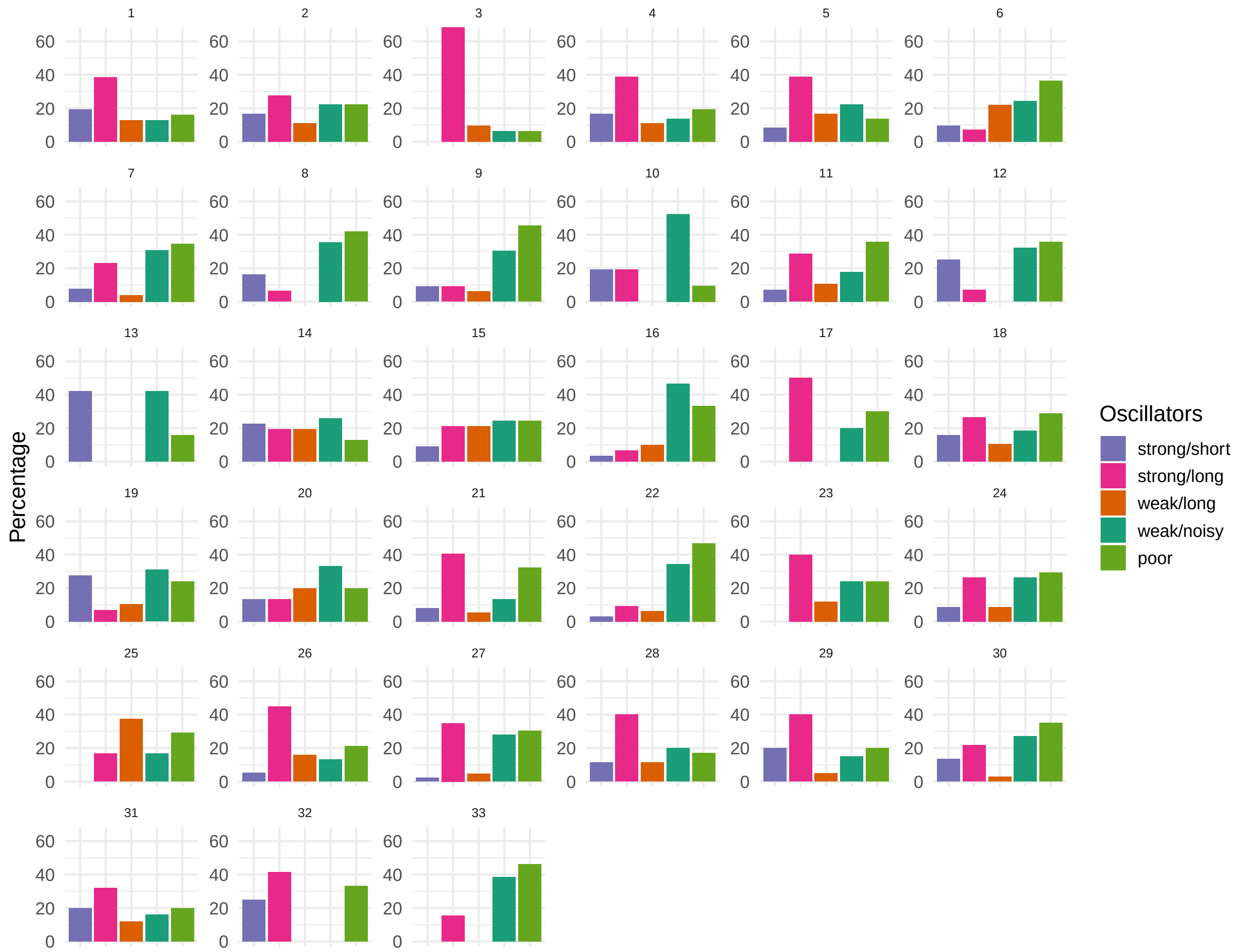

Fig V. Different oscillator types across each of the 33 nocodazole washout treated cells.

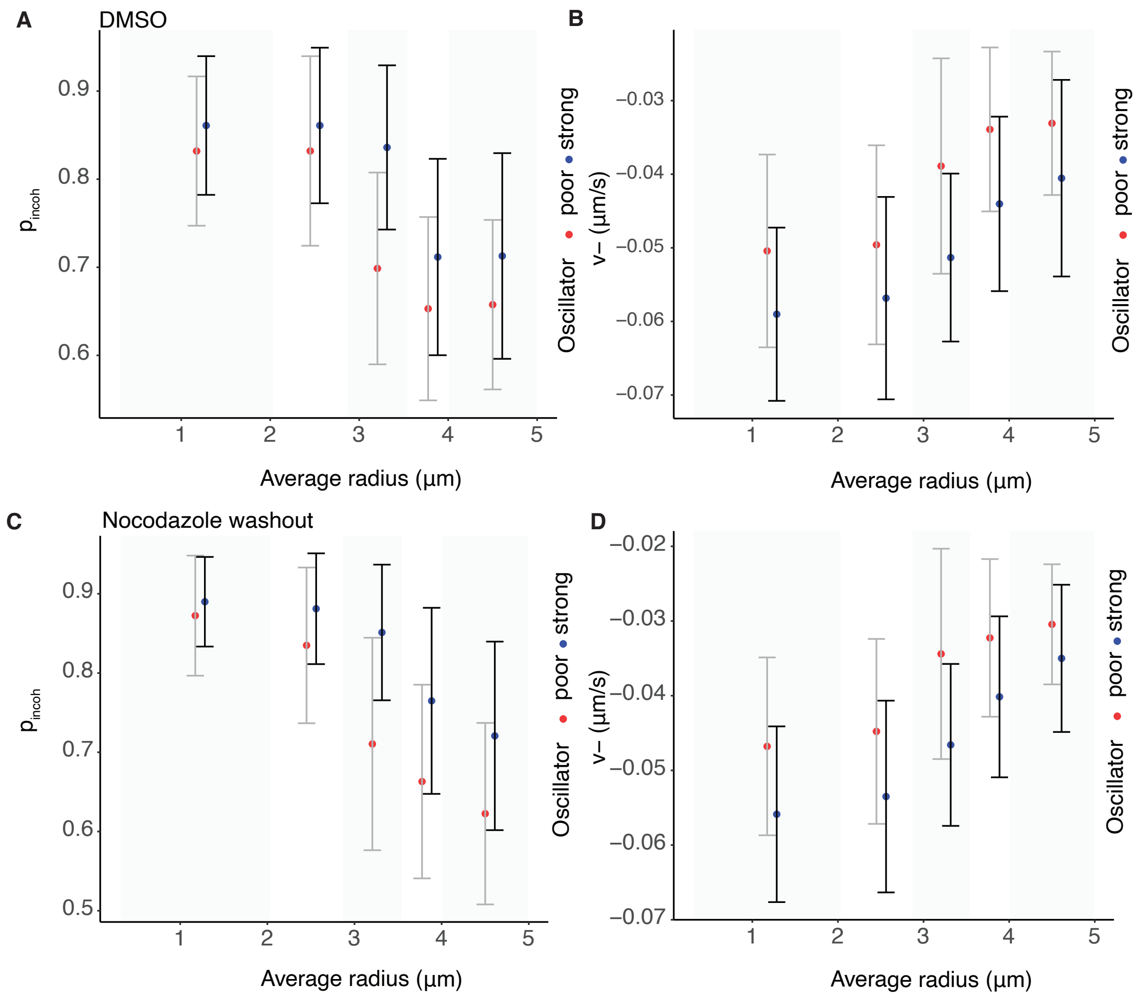

Fig W. Spatial trends in the MPP of the poor and strong oscillator clusters. Data is partitioned into 5 groups according to average radial distance in the MPP of the KT pair. Groups are equisized and defined by the radial partitions: $[0, 2.03], (2.03, 2.95], (2.95, 3.58], (3.580, 4.01], (4.01, 5.03]$. **A, C** The probability of remaining incoherent between frames, p_{incoh}. **B, D** K-fiber pulling force v_{-}. Treatments are **A, B** DMSO and **C, D** nocodazole washout treated cells. Median and standard deviation of each partition is plotted for poor oscillators (red), strong oscillators (blue). Parameters inferred on the asymmetric $M^{a}_{v\pm}$ model.

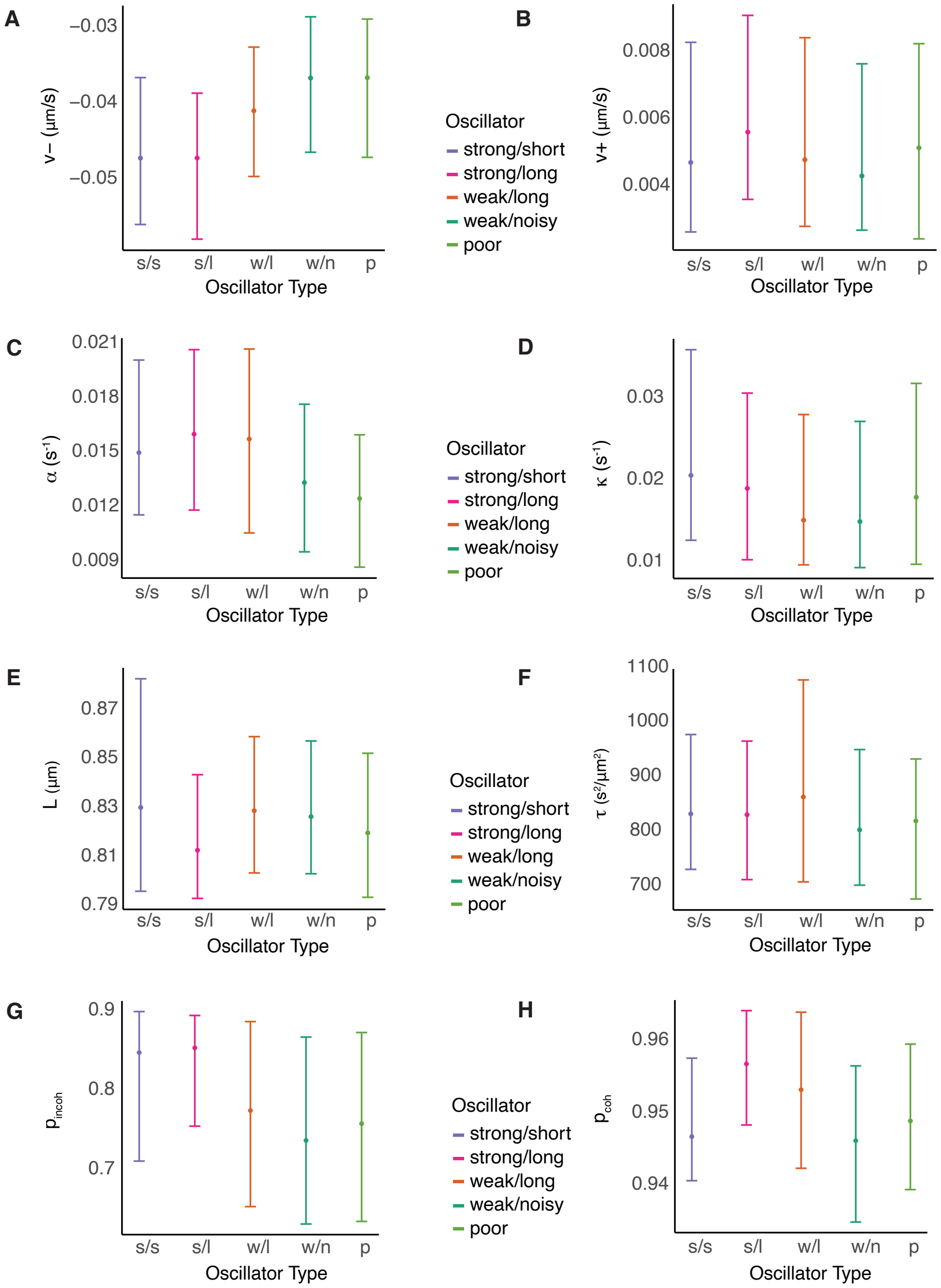

Fig X. Biophysical parameter variation with oscillation quality (nocodazole washout). Cluster median and standard deviation of stated parameter (posterior median) for the asymmetric $M^a_{v\pm}$ model are plotted by oscillator type. We observe a decreasing trend in pulling strength, $|v_-|$ and the probability of remaining incoherent between frames, p_{incoh}, as the quality of oscillation deteriorates. Other parameters show little variation. Figure compliments Fig. 12 for DMSO treated cells in main text.

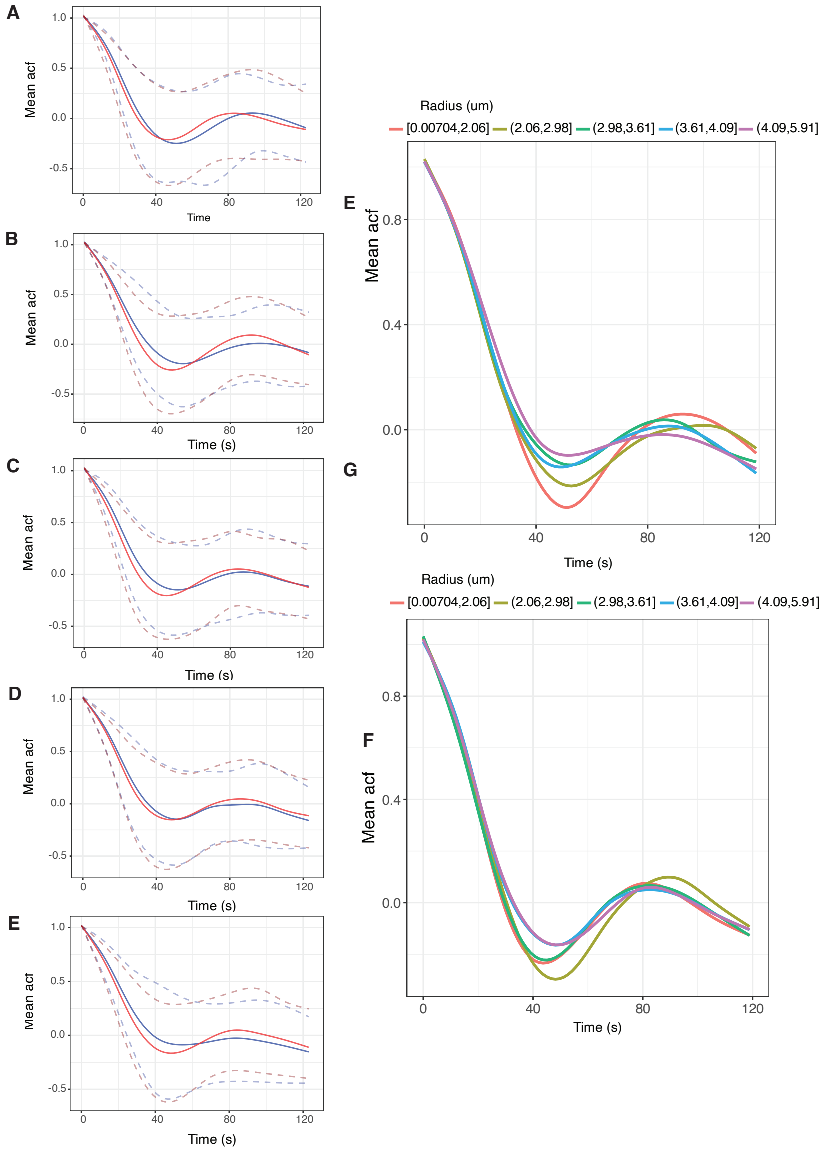

Fig Y. Autocorrelation plot analysis across the metaphase plate: **A-E** Autocorrelation plots for DMSO (blue) and nocodazole washout (red) treated cells. Plots A to E present the mean autocorrelations for sister pairs that belong in one of five groups defined by their radial distances, *i.e.,* $[0, 2.07], (2.07, 2.97], (2.97, 3.59], (3.59, 4.01], (4.01, 5.20]$ respectively. Dashed lines denote the 5% and 95% autocorrelations percentile for each time point. **E, F** Summary of mean autocorrelation plots for DMSO and nocodazole washout treated cells for all 5 classes of radial distances.

C Methodology

C.1 Data processing and Quality Control

We analyse mitosis in immortalised human retinal pigment epithelial cells (RPE1), a karyotypically stable, non-transformed cell line, using Lattice Light Sheet Microscopy (LLSM, [83]). We achieve a high signal-to-noise ratio and a temporal resolution of 2s/frame; this enables analysis of mitosis dynamics in a normal human cell line whilst ensuring minimal photobleaching and phototoxicity (all our cells transition to anaphase). We demonstrate near-complete 3D tracking of the 46 kinetochore pairs for up to 15 mins, and parametrise the models by fitting to KT trajectories.

Kinetochore tracking is performed using the software package KiT v3.0. The tracking algorithm proceeds by detecting candidate spots via a constant false alarm rate (CFAR) detection method, [43], to set a KT-wise dynamic threshold per image frame in a movie. Candidate spot locations are refined by fitting a Gaussian mixture model. Spot locations are linked between frames by solving a linear assignment problem using the Hungarian algorithm, with motion propagation via a Kalman filter. A "track" refers to a temporally ordered set of KT positions (coordinates) associated with a single KT across sequential image frames, constructed by linking detections frame-to-frame. A "gap" is defined as one or more consecutive frames within a track during which the KT was not detected; tracks are permitted to contain gaps of up to 3 frames, after which the track is considered terminated.

Tracked kinetochores are paired by solving a second linear assignment problem. Sister kinetochore pairing used spatial and temporal trajectory data. Sister kinetochores are closer (d_{ij}, average distance) and exhibit less distance variation (v_{ij}, distance variance) than non sisters. In the presence of a metaphase plate, their connecting vector aligns with the plate's normal (α_{ij}, average angle). Pairing costs were calculated as the product $d_{ij}v_{ij}$ without a plate or $d_{ij}v_{ij}\alpha_{ij}$ with a plate. Trajectories had to overlap for at least 10 frames, and the pairing configuration with the minimum global cost was selected.

Detection and tracking was near complete.

The tracklet plot, Fig 1C of the main text, shows that 82 KTs were tracked throughout the movie, whilst a total of 100 tracks were recorded (tracks are allowed to have gaps of at most 3 missing frames). Additionally, 43 KT pairs were tracked for at least 75% of the movie, with 38 KTs remaining paired for the entire duration of metaphase (sisters are allowed gaps of at most 3 frames), Fig 1D,E of the main text. The scatter plot of duration of pairing against duration either sister exists demonstrates near complete pairing over the duration of KT detection, Fig 1F of the main text. The average sister track length for this movie was 705 s. Spot detection and tracking performance are unaffected by the transition to anaphase.

36 RPE1 cells were imaged during metaphase-anaphase of varying durations in DMSO. A quality control filter was applied to ensure good sister pair coverage per cell. Specifically, at least 30 sister pairs had to be tracked for 75% of the movie length, resulting in 31 cells meeting the criteria and 1281 sister pair kinetochores being tracked. On average, 40 sister pairs per cell were tracked (quartiles: Q1 = 38.5, Q3 = 43), with both sisters tracked for at least 200 seconds (100 frames).

Prior to fitting the models, we implemented a data quality control. We discarded cells with a low coverage of sister pairs or a very high proportion of poor oscillators (indicating poor health), and for the metaphase-anaphase model, if there was insufficient anaphase. This reduced the analysed cells from 36 DMSO treated cells to 28 cells. We also filtered KT-pair trajectories, requiring no more than 20% missing data, and cell videos are required to have at least 120 metaphase frames. Trajectories with poor inference, identified by severe divergences in STAN, were excluded, and cells were discarded if a high proportion of sister pair trajectories showed frequent divergences, resulting to a further reduction of the analysed cells to 26. While divergences indicate the model isn't fully capturing the dynamics, they were rare ($\sim$1.5%, with slight variations between the different models). Hence, in the model analysis there are slight

differences in the number of trajectories considered due to performance issues. For the metaphase dynamics models (that don't include the anaphase transition), we truncated the trajectories to metaphase. Specifically, we used a change point model to detect the anaphase transition per KT pair, Section E of S1 Appendix. Then we truncated 30s before this, to ensure the cell is in metaphase. For nocodazole washout treated data, we have initially tracked 51 cells. Applying our filtering criteria of sister pair coverage, poor oscillators and sufficient anaphase part as in DMSO treated cells, we ended up with 33 nocodazole washout treated cells.

C.2 Likelihood and posterior distribution computation

The metaphase dynamics of Eq (1) in the main text, are described in detail in [18]. The model is a discretised system of equations – discrete measurement time with equal time steps:

$$\left(X^1_{t+\Delta t} - X^1_t\right) = \Delta t \left(-v_{\sigma^1_t} - \kappa\left(X^1_t - X^2_t - L\cos\theta_t\right) - \alpha X^1_t\right) + \sqrt{\Delta t}\mathcal{N}\left(0, \tau^{-1}\right),$$
$$\left(X^2_{t+\Delta t} - X^2_t\right) t = \Delta t \left(+v_{\sigma^2_t} - \kappa\left(X^2_t - X^1_t + L\cos\theta_t\right) - \alpha X^2_t\right) + \sqrt{\Delta t}\mathcal{N}\left(0, \tau^{-1}\right),$$

The kinetochore pair K-fiber state, σ_t, (hidden), takes values in the 4 states,

$$(\sigma^1, \sigma^2) \in \{(+,+), (+,-), (-,+), (-,-)\}, \tag{1}$$

and has transition matrix P (parametrised by p_{coh}, p_{icoh}, the probabilities of remaining in the same state (either coherent or incoherent).

In sum, the observations obey a Stochastic Differential Equation (SDE), with the observation at time t depends only on the previous observation x_{t-1} and the state at time t, while the hidden states are described by the transition probability matrix.

$$x_t \mid \sigma_t, x_{t-1} \sim \mathcal{N}\left(\Delta t f_{\sigma_t}\left(x_{t-1}\right), \sqrt{\Delta t}/\tau\right) \quad \text{and} \quad \sigma_t \mid \sigma_{t-1} \sim \text{categorical}\left(P\right).$$

with

$$f^k_i\left(\sigma^i_k\right) = (-1)^k v_{\sigma^k_i} + (-1)^k \kappa\left(d_i - L\cos\left(\theta_i\right)\right) - \alpha\left(x^k_i\right)$$

The likelihood is given by

$$L\left(x^1, x^2 \mid v_\pm, \kappa, \alpha, \tau, L, \sigma^k_i, \theta_i\right) = (2\pi)^{-\frac{1}{2}(n-1)} \tau^{\frac{1}{2}(n-1)} \prod_{k=1,2}\left(\exp\left(-\frac{\tau}{2}\sum_{i=1}^{n-1}\left(\left(dx^k_i - \Delta t f^k_i\left(\sigma^i_k\right)\right)^2\right)\right)\right)$$

This model can be modified accordingly to allow for asymmetry, as in Eq (3) of the main text, and time dependency, as in Eq (5) of the main text. The likelihood calculation for the asymmetric model is straightforward and hence is omitted. However, while the generalization to asymmetric models is relatively straightforward, the increased complexity lead us to use the following transformations for velocities

$$v^1_{+/-} = v^{mean}_{+/-} + d_{+/-} \quad \text{and} \quad v^2_{+-} = v^{mean}_{+/-} - d_{+/-}, \quad \text{with} \quad -2v^{mean}_{+/-} \leq d_{+/-} \leq 2v^{mean}_{+/-},$$

which improve the mixing of the Hamiltonian Monte Carlo algorithm. The likelihood for the full time dependent model is shown below.

$$\begin{aligned} & L\left(x^1, x^2 \mid v_{\pm,0}, \kappa_0, \alpha_0, \tau_0, L, v_{\pm,1}, \kappa_1, \alpha_1, \tau_1, \sigma^k_i, \theta_i\right) \\ &= (2\pi)^{-\frac{1}{2}(n-1)} \tau_0^{\frac{1}{2}(n-1)} \exp\left(\tau_1 \frac{n(n-1)}{2}\Delta t\right) \prod_{k=1,2}\left(\exp\left(-\frac{\tau_0}{2}\sum_{i=1}^{n-1}\left(\exp\left(\tau_1 i\Delta t\right)\left(dx^k_i - \Delta t f^k_i\left(\sigma^i_k\right)\right)^2\right)\right)\right) \\ & f^k_i\left(\sigma^i_k\right) = (-1)^k v_{\sigma^k_i,0}\exp\left(v_{\sigma^k_i,1} i\Delta t\right) + (-1)^k \kappa_0 \exp\left(\kappa_1 i\Delta t\right)\left(d_i - L\cos\left(\theta_i\right)\right) - \alpha_0\exp\left(\alpha_1 i\Delta t\right)\left(x^k_i\right) \end{aligned} \tag{2}$$

C.3 Scaled Forward - Backward Algorithm

We leverage a modified Forward-Backward (FB) algorithm, [84], to calculate the likelihood and make inference on the parameters. In particular, we use the Scaled Forward - Backward algorithm within the STAN environment, [85].

The FB algorithm is designed to exploit via recursion the conditional independencies in the HMM. The forward likelihood is defined as $l_t\left(i\right) = P\left(x_{1:t}, \sigma_t = i \mid \theta\right)$, which is the joint likelihood of the data $x_{1:t}$ and the state $\sigma_t = i$ averaging over the states up to time $t-1$. The likelihood contribution from data $x_{1:t}$ is just the sum $l_t^* = \sum_i l_t(i)$. Let $f_t = P\left(x_t \mid x_{1:t-1}\right)$, $\eta_{j,t} = P\left(x_t \mid \ x_{1:t-1}, \sigma_t = j; \theta\right)$, be the likelihood contribution of an observation, x_t under all states j, $P\left(\sigma_t = i \mid \sigma_{t-1} = j\right)$ the probability that at time t the state will be i given at $t-1$ was at state j, as given by the HMM transition probability matrix and θ all the unknown parameters. A recursive relationship can be derived as follows

$$\begin{aligned}
l_t\left(i\right) &= P\left(x_{1:t}, \sigma_t = i \mid \theta\right)\\
&= \sum_j P\left(x_t, x_{1:t-1}, \sigma_t = i, \sigma_{t-1} = j; \theta\right)\\
&= \sum_j P\left(x_t \mid x_{1:t-1}, \sigma_t = i, \sigma_{t-1} = j; \theta\right) P\left(\sigma_t = i, \sigma_{t-1} = j, x_{1:t-1}\right)\\
&= \sum_j P\left(x_t \mid x_{1:t-1}, \sigma_t = i; \theta\right) P\left(\sigma_t = i \mid \sigma_{t-1} = j, x_{1:t-1}\right) P\left(\sigma_{t-1} = j, x_{1:t-1}\right)\\
&= \sum_j P\left(x_t \mid x_{1:t-1}, \sigma_t = i; \theta\right) P\left(\sigma_t = i \mid \sigma_{t-1} = j\right) P\left(\sigma_{t-1} = j, x_{1:t-1}\right)\\
&= \sum_j P\left(x_t \mid x_{1:t-1}, \sigma_t = i; \theta\right) P\left(\sigma_t = i \mid \sigma_{t-1} = j\right) l_{t-1}(j)\\
&= \sum_j \eta_{i,t}\left(x_t; \theta\right) P\left(\sigma_t = i \mid \sigma_{t-1} = j\right) l_{t-1}(j) \qquad (3)
\end{aligned}$$

It is then obvious that the log-likelihood is the sum of all states at time $t = T$, *i.e.,*

$$\log\left(L\left(x\right)\right) = \log\left(P\left(x_{1:T}; \theta\right)\right) = \log\left(\sum_t f_t\right) = \log\left(\sum_i l_T\left(i\right)\right)$$

In our codes, we are using log-likelihoods for all our calculations as they are more numerically stable.

Now, let $\xi_{j,t} = P(\sigma_t = j \mid x_{1:t}; \theta)$ be the probability of being in state σ_t at time t given all the data up to that time. With initial condition $\xi_0 = P(\sigma_0 = j \mid \theta) = [0, 0.5, 0.5, 0]^T$, *i.e.,* at time $t = 0$ it is equally probable to be in one of the coherent states $(+-, -+)$. Observe that

$$l_t(i) = P\left(x_{1:t}, \sigma_t = i\right) = P\left(\sigma_t = i \mid x_{1:t}\right) P\left(x_{1:t}\right) = \xi_{i,t} P\left(x_{1:t}\right) = \xi_{i,t} \sum_i l_t\left(i\right)$$

and note that $f_t = P\left(x_t \mid x_{1:t-1}\right) = \sum_i \sum_j \eta_{i,t}\left(x_t; \theta\right) P\left(\sigma_t = i \mid \sigma_{t-1} = j\right) \xi_{j,t-1}\left(i\right)$ then Eq (9) can be modified and calculate the forward probabilities $\xi_{i,t} = \frac{l_t(i)}{\sum_i l_t(i)}$ instead and derive our final recursive relationship

$$\begin{aligned}
\xi_{i,t}\left(i\right) &= \frac{1}{P\left(x_{1:t}\right)} \sum_j \eta_{i,t}\left(x_t; \theta\right) P\left(\sigma_t = i \mid \sigma_{t-1} = j\right) \xi_{j,t-1}\left(i\right) P\left(x_{1:t-1}\right)\\
&= \sum_j \eta_{i,t}\left(x_t; \theta\right) P\left(\sigma_t = i \mid \sigma_{t-1} = j\right) \frac{\xi_{j,t-1}\left(i\right)}{P\left(x_t \mid x_{1:t-1}\right)}\\
&= \sum_j \eta_{i,t}\left(x_t; \theta\right) P\left(\sigma_t = i \mid \sigma_{t-1} = j\right) \frac{\xi_{j,t-1}\left(i\right)}{f_t}
\end{aligned}$$

In our setting, we have

$$\eta_{\sigma_t,t} \equiv \mathcal{N}\left(x_t \mid x_{t-1} + \Delta t\, M x_{t-1} + \Delta t\, \tilde{M} \sigma_t + \Delta t \mu, \Delta t \tau^{-1}\right)$$

where M, $\tilde{M}$, and μ are derived from the linear SDE and given by

$$M = \begin{bmatrix} \kappa - \alpha & \kappa \\ \kappa & -\kappa - \alpha \end{bmatrix}$$

and

$$\tilde{M} = \begin{bmatrix} -v_{+} & -v_{+} & -v_{-} & -v_{-} \\ v_{+} & v_{-} & v_{+} & v_{-} \end{bmatrix}$$

$$\mu = [\kappa L \cos(\theta_t), \kappa L \cos(\theta_t)]^T.$$

In this notation, the state variable $x_t = [X_t^1, X_t^2]^T$ represents the positions of both sisters in a pair at time t and the state $\sigma_t \in \{[1,0,0,0]^T, [0,1,0,0]^T, [0,0,1,0]^T, [0,0,0,1]^T\}$ corresponding to the states $\{++, +-, -+, --\}$ for the sister kinetochore pair at time t.

In order to make statements about switching events, we need to consider sequences of states forming a pattern corresponding to coherent switches from one coherent state to another via an intermediate state. To address this, we sample from the full hidden state sequence given all the data, and assess this for switches. We use the stochastic backward simulation to simulate/sample the hidden states, which is a rapidly mixing algorithm, [84]. Observe that

$$P(\sigma_{1:T} \mid x_{1:T}, \theta) = P(\sigma_T \mid x_T) \prod_{t=1}^{T-1} P(\sigma_{T-t} \mid \sigma_{T-t+1} x_{1:T})$$

Then, keeping the terms that depend on σ_t, the it is easy to show the following

$$\begin{aligned} P(\sigma_t = i \mid \sigma_{t+1} = j, x_{1:T}) &= \frac{P(\sigma_t = i, \sigma_{t+1}, x_{1:T})}{P(\sigma_{t+1} = j, x_{1:T})} \\ &\propto P(\sigma_t = i, x_{1:T}) P(\sigma_{t+1} = j \mid \sigma_t = i) P(x_{t+1:T} \mid \sigma_{t+i}) \\ &\propto P(\sigma_t = i, x_{1:t}) P(\sigma_{t+1} = j \mid \sigma_t = i) \\ &\propto \xi_{i,t} P(\sigma_{t+1} = j \mid \sigma_t = i) \end{aligned} \tag{4}$$

The strategy for the backward sampling algorithm is therefore to sample initially from $p(\sigma_T \mid x_{1:T}) = \xi_{T,i}$ as derived in the forward recursion and subsequently to simulate backward in time from $T-1$ to 1 via the conditional distribution given in Eq (10).

C.4 Missing data treatments

Suppose we have missing observations. Let's assume that the data comprises two sections $[1 : T_1]$ and $[T_2 : T]$, with $T_2 = T_1 + 1 + l > T_1$, *i.e.,* a gap of size $l \geq 1$ where data on both sisters is missing. Given the Markovian property of the observations, we can construct the multi-time point conditional, $P(X_{t+k\Delta t}, \sigma_{t+k\Delta t} \mid X_t, \sigma_t, \theta)$. Incorporating the dependence on the hidden state path, we get the following expression,

$$P(X_{t+k\Delta t}, \sigma_{t+1:t+k\Delta t} \mid X_t, \sigma_t, \theta) = P(\sigma_{t+1:t+k\Delta t} \mid \sigma_t, \theta) P\left(X_{t+k\Delta t} \mid X_t, \sigma_{t:t+(k-1)\Delta}, \theta\right)$$

Thus, we have the full history dependency $P(X_{T_2} \mid X_{1:T_1})$ across a gap.

We consider then obtain the likelihood,

$$L(x_{1:T_1 \cup T_2:T}, \sigma_{1:T} \mid \theta) = P(x_{1:T_1}, \sigma_{1:T_1} \mid \theta) P(x_{T_2}, \sigma_{T_1+1:T_2} \mid x_{T_1}, \sigma_{T_1}, \theta) P(x_{T_2+1:T}, \sigma_{T_2:T} \mid x_{T_2}, \sigma_{T_2}, \theta)$$

and the marginalised likelihood $L(x_{1:T_1 \cup T_2:T} \mid \theta)$ given by summation over the hidden state $\sigma_{1:T}$.

There are a lot of different methods that we can utilise that deal with the gap (middle) terms,

$$P(x_{T_2}, \sigma_{T_1+1:T_2} \mid x_{T_1}, \sigma_{T_1}, \theta)$$

, and each comes with its own set of strengths and weaknesses.

In brief, there we have four options. First, we could fill in the missing values with linear interpolation, secondly, treat sections as independent, *i.e.,* multiply the likelihoods for each section, thirdly, remove data points from the likelihood calculation but retain hidden state propagation, fourthly, impute the hidden values by sampling from the posterior predictive distribution

$$P\left(x_{missing} \mid x_{obs}, \theta\right)$$

(using a Brownian Bridge sampler). The first two methodologies are approximating the actual time-series, as if there were no missing values and thus, they introduced a bias which can be, in some cases, non- negligible. The third method, doesn't utilise all the available information, while the fourth option – even though is the most flexible option for extending to missing values of a single sister – it is likely computationally expensive for large gaps. Taking into account the advantages and limitations of each method, we have chosen to work with the third method, which we have found that it optimally balances the trade-off between reducing computational complexity and introducing estimation bias.

C.5 Reduced likelihood method

Consider the full likelihood. The gap term can be expressed as,

$$P\left(x_{T_2}, \sigma_{T_1+1:T_2} \mid x_{T_1}, \sigma_{T_1}, \theta\right) = P\left(x_{T_2} \mid x_{T_1}, \sigma_{T_1:T_{T_2-1}}, \theta\right) P\left(\sigma_{T_1+1:T_2} \mid \sigma_{T_1}, \theta\right)$$

The idea here is to exclude the propagation term for x_{T_2}, i.e, use the reduced full likelihood

$$L_r\left(x_{1:T_1 \cup T_2:T}, \sigma_{1:T} \mid \theta\right) = P\left(x_{1:T_1}, \sigma_{1:T_1} \mid \theta\right) P\left(\sigma_{T_1+1:T_2} \mid \sigma_{T_1}, \theta\right) P\left(x_{T_2+l:T}, \sigma_{T_2:T} \mid x_{T_2}, \sigma_{T_2}, \theta\right).$$

giving the corresponding reduced marginalised likelihood $L_r\left(x_{1:T_1 \cup T_2+1:T} \mid \theta\right)$ (here we removed x_{T_2} in notation as information in knowing x_{T_2} is not used. However, the likelihood depends on x_{T_2}, as it is part of the history).

We *propose* to modify the forward algorithm, based on the above lines. In particular, we change the forward likelihood calculation by assuming that the likelihood contribution of the missing observations is 0 and hence, the likelihood contribution at that specific time point, is only defined by the transition probabilities.

We have made a series of experiments, to proof that this methodology is robust and gives unbiased results. In particular, we have compared the likelihood estimations of different simulated data sets, with varying missing data proportions and different distributed missing values (changing the size of the gaps). Also, we have compared the results of this methodology with the results of the first method,*i.e.,* filling the missing data by linear interpolation, which gave similar results, when the missing values' gaps were small.

In Table M of S1 Appendix, we present a case study where we have simulated 10 different time-series of length $T = 180$ and we have assessed whether the different mean posterior estimates where greater than the actual values with probability $p \neq 0.5$. To asses this hypothesis, we performed a two-sided binomial test. In all of our experiments the "reduced likelihood computation" was accurate, with small estimation bias.

C.6 Prior distributions

We impose broad prior distributions on the parameters of the biophysical model, as shown in Table N of S1 Appendix. For the natural length of the spring, L, we impose an informative prior based on an additional nocodazole washout experiment (Nocodazole interferes with polymerization of microtubules). This avoids an unidentifiabilty in the model as in [18]. Additionally, we use an informative prior for the time of anaphase, t_A, based on first fitting a changepoint model (with a uniform prior on t_A) to get an initial estimate for anaphase onset to guide the biophysical model and avoid exploring parameter space corresponding to pathological behaviour such as anaphase at the start or end of movies.

Actual values	No missing			Single missing			Double missing			Triple missing			All-in-a-row		
	Mean	Variance	p-value	Mean	Variance	p-value	Mean	Variance	p-value	Mean	Variance	p-value	Mean	Variance	p-value
$\tau^1 = 500$	535.53	72.80	0.75	536.75	82.07	1.00	514.33	80.23	0.75	542.16	79.12	1.00	536.98	77.69	1.00
$\tau^2 = 300$	289.01	38.85	0.35	289.82	43.34	1.00	294.92	43.25	1.00	294.84	41.93	1.00	287.27	41.11	0.75
$\kappa = 0.02$	0.022	0.0090	0.35	0.021	0.0097	0.22	0.022	0.0095	0.35	0.021	0.0092	1.00	0.022	0.0097	1.00
$\alpha = 0.06$	0.064	0.0084	1.00	0.061	0.0097	0.75	0.062	0.0096	1.00	0.0062	0.0091	1.00	0.064	0.0092	0.75
$v_-^1 = -0.04$	-0.040	0.008	1.00	-0.039	0.008	0.11	-0.039	0.008	0.35	-0.040	0.008	1.00	-0.040	0.008	1.00
$v_-^2 = -0.06$	-0.059	0.008	1.00	-0.060	0.009	0.51	-0.059	0.008	0.75	-0.058	0.008	0.75	-0.060	0.008	0.75
$v_+^1 = 0.02$	0.020	0.006	0.75	0.019	0.007	1.00	0.018	0.007	1.00	0.019	0.006	1.00	0.019	0.007	1.00
$v_+^2 = 0.042$	0.042	0.007	1.00	0.041	0.007	0.11	0.042	0.007	1.00	0.040	0.007	1.00	0.042	0.007	0.75
$L = 0.75$	0.789	0.119	0.02*	0.789	0.119	0.002*	0.789	0.118	0.02*	0.789	0.119	0.001*	0.789	0.119	0.02*
$p_{icoh} = 0.75$	0.736	0.074	0.35	0.757	0.079	0.35	0.715	0.089	0.35	0.744	0.080	1.00	0.736	0.075	1.00
$p_{coh} = 0.93$	0.914	0.024	0.35	0.915	0.026	1.00	0.907	0.027	1.00	0.914	0.025	1.00	0.912	0.025	0.51

Table M. Summary of the case study for checking the bias of our algorithm when we have no missing data and 10% missing data points under various settings, *i.e.,* single missing data points, double missing data points, triple missing data points and all-in-a-row missing data points. The case study comprises ten different simulated time-series of length $T = 180$, from a common multivariate distribution, in an effort to Average posterior mean MCMC estimates, average posterior MCMC variance and p-values of the two-sided exact binomial test presented. The null hypothesis is that there is 0.5 probability that the posterior mean estimates are greater than the actual value. Our algorithm gives unbiased results

**Parameter**	**Prior**	**Support**
**Symmetric-Asymmetric Model**		
α	$\mathcal{N}\left(\mu = 0.01, \sigma^2 = 0.1^2\right)$	$[0, \infty)$
κ	$\mathcal{N}\left(\mu = 0.05, \sigma^2 = 0.1^2\right)$	$[0, \infty)$
v_-	$\mathcal{N}^0\left(\mu = -0.03, \sigma^2 = 0.1^2\right)$	$(-\infty, 0]$
v_+	$\mathcal{N}_0\left(\mu = 0.3, \sigma = 0.1^2\right)$	$[0, \infty)$
p_{icoh}	$\mathcal{B}eta\,(2, 1)$	$(0, 1)$
p_{coh}	$\mathcal{B}eta\,(2.5, 1)$	$(0, 1)$
L	$\mathcal{N}_0\left(\mu = 0.79, \sigma^2 = 0.119^2\right)$	$[0, \infty)$
$\tau^{1,2}$	$\mathcal{G}amma\left(0.5, 10^{-3}\right)$	$[0, \infty)$
**Asymmetric Model**		
v_-^{mean}	$\mathcal{N}^0\left(\mu = -0.03, \sigma^2 = 0.1^2\right)$	$(-\infty, 0]$
d_-	$\mathcal{N}_{-2v_-^{mean}}^{2v_-^{mean}}\left(\mu = 0, \sigma^2 = 0.1^2\right)$	$[-2v_-^{mean}, 2v_-^{mean}]$
v_+^{mean}	$\mathcal{N}_0\left(\mu = 0.3, \sigma = 0.1^2\right)$	$[0, \infty)$
d_+	$\mathcal{N}_{-2v_+^{mean}}^{2v_+^{mean}}\left(\mu = -0.03, \sigma^2 = 0.1^2\right)$	$[-2v_+^{mean}, 2v_+^{mean}]$
**Temporal Parameters**		
p_1	$\mathcal{N}\left(\mu = 0, \sigma^2 = 0.00382^2\right)$	$[0, \infty)$
$*$ Note: p_1: any temporal variable		
**Metaphase-Anaphase Model**		
$v_{anaphase}^{1,2}$	$\mathcal{N}^0\left(\mu = 0.3, \sigma^2 = 0.1^2\right)$	$[0, \infty)$
$t_{anaphase}$	$\mathcal{N}_t\left(\mu = t^*, \sigma^2 = (3\Delta t)^2\right)$	$[0, \infty)$
$*$ Note 1: $\mathcal{N}_t$: lower truncated Normal distribution at t		
$*$ Note 2: $\mathcal{N}^t$: upper truncated Normal distribution at t		
$*$ Note 3: $\mathcal{N}_{t_1}^{t_2}$: lower and upper truncated Normal distribution at t_1 and t_2 respectively		
$*$ Note 4: t^*: median (initial) estimate of anaphase time over all KT pairs in a cell		

Table N. Priors for the symmetric, asymmetric, temporal, metaphase-anaphase models.

C.7 Statistical Tests

The tests that have been used throughout this study are summarised in Table O of S1 Appendix, describing each test and the abbreviation we use when quoting the p-values in the text.

Test	Type	Null Hypothesis (H_0)	Abbreviation
Kolmogorov-Smirnov	Non-parametric	Equality of 2 continuous distributions	KS
Mann-Whitney (Wilcoxon rank)	Non-parametric	Equality of 2 distributions	MW
Pearson's (χ^2)	Non-parametric	Count numbers come from a Multinomial distribution	Pearson
		Goodness of fit	
		Homogeneity of counts for ≥ 2 groups under the same variable	
		Independence of 2 variables (via Contingency table)	
Spearman's	Non-parametric	No monotonic relationship between 2 ranked variables	Spearman
Kendall's	Non-parametric	Dependence of 2 distributions is 0	Kendall
Pearson's Correlation	Parametric	No linear correlation	Corr
Binomial	Exact test	Two categories occur with equal frequency	Binom
Hoeffding's	Non-parametric	Independence of 2 continuous distributions	Hoef
Distance Covariance	Non-parametric	Multivariate independence	DC
Brown-Forsythe test	Non-parameteric	Variances of 2 groups are homogeneous (equal)	BF
Cochran-Mantel-Haenszel test	Non-parametric	No association between a binary predictor and a binary outcome	CMH
		while controlling for confounding factors/ stratification	
Z-test	Parametric	Mean of a population is μ_0	Ztest
		Equality of the means of 2 populations	
F-test	Parametric	Equality of variances	Ftest

Table O. Summary of tests used in this analysis. The first column refers to the test, the second the type of the test, *i.e.,* assumptions, the third column describes the null hypothesis and hence the test, while the last column states the abbreviation that is used throughout this study, when referring to p-values.

C.8 Implementation

All our models were implemented in STAN, [85] and fitted using the R-package "rstan". STAN is based on a C++ language which obtains posterior samples of the Bayesian models by defining the aforementioned likelihoods and priors. We then used R to access the output, evaluate the posterior densities and likelihoods to make inference on the parameters.

Model selection

Model selection is based on pairwise comparison of models using the Bayes Factors (BF). The BF for model M' relative to a simpler model M is is just the ratio of the models' marginal likelihoods

$$B = \frac{\pi (D \mid M')}{\pi (D \mid M)} = \frac{P (M' \mid D) P (M')}{P (M \mid D) P (M)}$$

Assuming that the models are equally probable a priori, *i.e.,* $P(M') = P(M)$, then the BF can be seen as the posterior odds ratio. We use the criteria of [54] and chose the more complex model if it had at least substantial preference over the simpler model ($BF > 3.2$). We confirmed that this gave reasonable false positive rates on a single example; the false positive rate (preferring (BF > 3.2) the asymmetric model when the original data is generated from the symmetric/vanillla model) was evaluated for the asymmetric $v_{\pm}$ model relative to the symmetric model, giving a false positive rate of around 1% on simulated data.

We compute the BF of all models relative to our vanilla model, Eq (1) of the main text. Then, we determine which models have a substantial preference over the base model. If there are more than one models with substantial preference, and all of these models have the same

complexity (*i.e.,* equal number of parameters), we choose the model with the highest BF. If the set of models with substantial preference, contains models with different complexities and the more complex models (*i.e.,* models with higher number of parameters), are nested to any of the other models in the set,then we compute the Bayes Factor of the complex-nested model relative to the simpler model and we choose the more complex model only when the preference of the complex model relative to simpler model is at least substantial and hence increasing the complexity is justified. Finally, if the set of models with substantial preference are models with different complexities but none of them is nested to another, then again we choose the model with the highest BF over the base model.

We use the “bridgesampling” package in R, which computes the log-marginal likelihood for every model, using the log-likelihood samples passed from STAN HMC samples (which were in turn computed in the Forward Backward algorithm). This package utilizes the bridge sampling, [53, 86]. Then, the BF is just the ratio of the marginal likelihoods.

Convergence diagnostics

Convergence and mixing of MCMC chains is assessed via the Gelman-Rubin $\hat{R}$ statistic, [87, 88], using only results where $\hat{R} < 1.05$ for all parameters.

Of $N = 82$ cells tracked, MCMC chains were run successfully for $N = 59$ cells, *i.e.,* 26 and 33 DMSO and nocodazole washout. Where MCMC chains failed to run this was due either to poor tracking results in that cell (insufficient tracked kinetochore pairs or existence of too many missing data) or long time series such that the MCMC chains progressed extremely slowly (failed to find the typical set). Of the $n = 1577$ kinetochore pairs across 59 cells where MCMC chains ran successfully, MCMC chains from $n = 27$ (1.5%) kinetochore pairs failed to converge as assessed by the $\hat{R}$ statistic, leaving estimates from $n = 1550$ kinetochore pairs.

Convergence proportions were slightly lower for the time dependent and metaphase-anaphase model, due to the increased complexity of these models.

D Anaphase transition: modelling and analysis

D.1 Modelling the metaphase-anaphase transition

To analyse anaphase further, we extended the asymmetric model (Eq (3) of the main text) to incorporate anaphase dynamics and an anaphase transition event, implemented similar to the metaphase-anaphase model in [61]. Specifically, we introduce an additional hidden state, A, the anaphase state with dynamics given by,

$$\begin{aligned} X^1_{t+\Delta t} &= X^1_t + v^1_A \Delta t + \sqrt{\Delta t} N\left(0, \tau^{-1}\right), \\ X^2_{t+\Delta t} &= X^2_t - v^2_A \Delta t + \sqrt{\Delta t} N\left(0, \tau^{-1}\right). \end{aligned} \tag{5}$$

where v^k_{A}, s^k are sister k's velocity and diffusion coefficient in anaphase. Since the sisters are no longer connected the spring forces are removed, whilst the velocity in anaphase may be different to the depolymerising K-fibre velocity. We also removed the mid-plane centralising forces from the model since the PEF decays over anaphase, although the decay dynamics is unknown. Our model is only appropriate for the part of anaphase when the speed is approximately constant and the separation between the descending KT clusters is not too large, since as they approach the poles they slow down. We only fitted the model to linear regions (truncating movies if there were signs of slow down). Anaphase portions were around 50 frames, enough to infer the anaphase speed and anaphase onset time.

The transition to anaphase is modelled as a smooth switch with a transition probability to A at time t given by,

$$p_{\mathrm{A}}(t) = \frac{1}{1 + \exp\left(-\left(t - t_{\mathrm{A}}\right)/\beta\right)}.$$

Thus, the transition to anaphase occurs around time t_{A} with $\beta = \Delta t/2$ (fixed) determining the range over which switching can occur. We assume that the anaphase state A is equally accessible from each of the other states, but transitions back from anaphase to metaphase are not possible, *i.e.,* state A is absorbing. Thus, once anaphase onset occurs the chromosomes segregate.

Switches between hidden states (see Fig 3B of the main text) occur at each time step according to the time dependent transition matrix (state order as Eq (7), with the anaphase state A in the last column/row),

$$\mathbf{P}(t) = \begin{bmatrix} p_{incoh}p_{incoh}q_{\mathrm{A}}(t) & p_{incoh}q_{incoh}q_{\mathrm{A}}(t) & p_{incoh}q_{incoh}q_{\mathrm{A}}(t) & q_{incoh}q_{incoh}q_{\mathrm{A}}(t) & p_{\mathrm{A}}(t) \\ p_{coh}q_{coh}q_{\mathrm{A}}(t) & p_{coh}p_{coh}q_{\mathrm{A}}(t) & q_{coh}q_{coh}q_{\mathrm{A}}(t) & p_{coh}q_{coh}q_{\mathrm{A}}(t) & p_{\mathrm{A}}(t) \\ p_{coh}q_{coh}q_{\mathrm{A}}(t) & q_{coh}q_{coh}q_{\mathrm{A}}(t) & p_{coh}p_{coh}q_{\mathrm{A}}(t) & p_{coh}q_{coh}q_{\mathrm{A}}(t) & p_{\mathrm{A}}(t) \\ q_{incoh}q_{incoh}q_{\mathrm{A}}(t) & p_{incoh}q_{incoh}q_{\mathrm{A}}(t) & p_{incoh}q_{incoh}q_{\mathrm{A}}(t) & p_{incoh}p_{incoh}q_{\mathrm{A}}(t) & p_{\mathrm{A}}(t) \\ 0 & 0 & 0 & 0 & 1 \end{bmatrix}$$

with $q_{coh} = 1 - p_{coh}$ and $q_{incoh} = 1 - p_{incoh}$ as before, and $q_{\mathrm{A}}(t) = 1 - p_{\mathrm{A}}(t)$, where p_{coh} and p_{incoh} are the probabilities of a kinetochore remaining in the coherent, respectively incoherent state over a time interval Δt, and p_A is the probability of transition to the anaphase state, A.

D.2 Inference of anaphase parameters

We fitted the anaphase model to 790 KT pair trajectories (out of 798 pairs, 99.0%, 26 cells in DMSO) requiring at least 20 frames in anaphase. At anaphase the sister pairs separate, with 2 clusters of KTs approaching respective poles. These clusters separate at the same speed in DMSO and nocodazole washout treated cells, Fig ZA of S1 Appendix, although nocodazole washout treated cell clusters spread substantially compared to DMSO, that have approximately constant cluster width, Fig ZB of S1 Appendix. Although K-fibers are depolymerising in anaphase, we observed occasional reversals in individual KT trajectories, whereby a short reversal of direction occurs, Fig ZC of S1 Appendix. The inference algorithm for this metaphase-anaphase model displayed more divergences than our base model, rising to 3% and reducing the analysable KT-pairs to 767. In addition, as this model doesn't incorporate events with transient anti-poleward KT movement in anaphase (reversals), which would reduce the inferred anaphase speed, we filtered out the KT pairs which had anaphase reversals by visual inspection. Hence, we report the fitting of the anaphase model to 611 KT pairs (of 767 pairs, 79.7%) in DMSO.

Inference of the anaphase model on nocodazole washout treated cells, (Eq (11) of the main text), had more divergences than in DMSO, *i.e.,* 835 out of 927 pairs had no divergences, (90.1%) in nocodazole washout, compared to 767 out of 790 (97.1%) in DMSO, consistent with the higher frequency of reversals under nocodazole washout treated cells, Fig ZD of S1 Appendix. We filtered out KT pair trajectories with reversals leaving 456 KT pairs (from 30 cells) for which the inference is reliable. Anaphase dynamics in nocodazole washout treated cells was similar to DMSO based on the reversal free trajectories, Fig AD of S1 Appendix with similar dispersion of anaphase onset times, and similar anaphase speeds.

An example of anaphase inference on an experimental trajectory is shown in Fig ABA in S1 Appendix, where both anaphase timing and the anaphase speed are inferred with high confidence and consistent with expert judgment. Within a cell we observe substantial variation in anaphase timing of KTs, Fig ABB in S1 Appendix, with an average standard deviation of 10.3 secs (range 5 secs to 32 secs) across the 26 cells. Anaphase timing varies with MPP location, Fig ABD in S1 Appendix, ($\rho = -0.27$, $p_{Corr} < 10^{-14}$), peripheral KT pairs entering anaphase earlier. The average anaphase pulling force was typically smaller than the average K-fiber pulling force, *i.e.,* 67% of the pulling force on average. This is expected since the model ignores the centralising force in anaphase, and the K-fiber pulling force typically decreases towards anaphase whilst we are comparing v_a to the average metaphase force v_-.

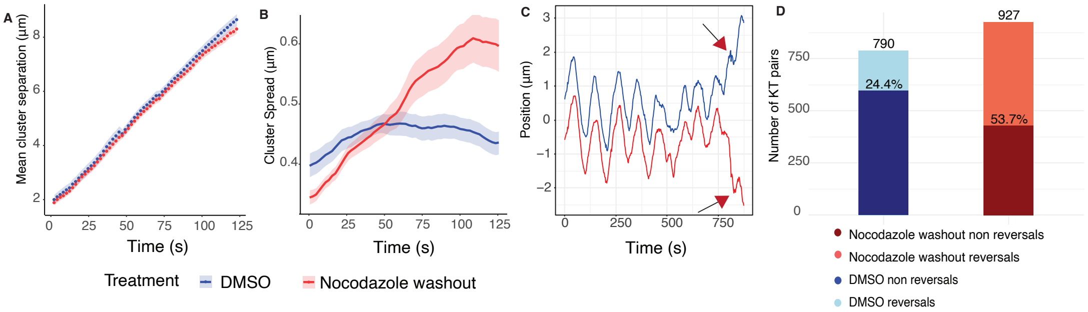

Fig Z. Anaphase clusters for DMSO (blue) and nocodazole washout (red) treated cells. Time $t = 0$ marks the median of estimated anaphase onset time per cell. **A** Mean cluster separation. Shaded areas denote the standard errors. **B** Cluster spreads during time and left axis based on 699 DMSO treated (blue) and 644 nocodazole washout treated (red) KT-pairs, which had long enough anaphase time points. **C** Example of a sister pair trajectory with reversals in anaphase. Arrows indicate reversal events. **D** Summary of KT-pairs with reversals and no reversals in DMSO (blue) and nocodazole washout cells (red). From 790 (927) KT-pairs 611 (465) pairs had no reversals, *i.e.,* 77.3% (49.2%) for DMSO (nocodazole washout treatment).

There is a weak correlation between v_- and v_a ($\rho = 0.17$, $p_{Corr} < 10^{-15}$), Fig ABF in S1 Appendix, although there is no trend in v_a across the MPP (Fig ABC in S1 Appendix), which is in stark contrast to the strong decrease in the strength of the pulling force with r. The only correlations of note are that the anaphase speed of sisters is correlated ($\rho = 0.39$, $p_{Corr} < 10^{-15}$), Fig ABH of S1 Appendix, and there is positive correlation ($\rho = 0.247$, $p_{Corr} < 10^{-9}$) between anaphase speed and anaphase time relative to median anaphase time, *i.e.,* sisters with a later transition to anaphase have a higher anaphase speed. Similar results were obtained if reversals were not filtered out.

The onset of anaphase, when kinetochore pairs begin to separate and segregate towards their respective spindle poles is tightly controlled temporally, [89], and appears entirely synchronous at low time resolution, but is in fact asynchronous, Fig ABB of S1 Appendix, (previously reported for RPE1 cells in [61, 90]). Peripheral sister-chromatids initiate anaphase earlier than central sister-chromatids by 4s in RPE1 cells, and have a lower anaphase speed, Fig ABC,D in S1 Appendix (there is positive correlation ($\rho = 0.247$, $p_{Corr} < 10^{-9}$) between anaphase speed and relative anaphase time). However, sister pairs with stronger pulling asymmetry are not biased towards having a higher anaphase speed, Fig AC of S1 Appendix. Further, we observed a weak correlation between the pulling speed v_-^k and v_a^k, $\rho = 0.174$, $p_{Corr} < 10^{-15}$, Fig ABF of S1 Appendix, thus although our estimated anaphase force (our model is ignoring PEF in anaphase) is the same order as the K-fiber pulling forces in metaphase, there is only a weak correlation between K-fiber pulling in metaphase and anaphase, whilst spatial trends in the MPP are distinctly different.

This likely reflects the fact that K-fibers in metaphase and anaphase are regulated by different mechanisms and the KTs are in different states, although it is also reminiscent of the anaphase speed governor, [73]. K-fibers in metaphase undergo dynamic instability and rarely have high coherence between MTs in the bundle, [72], whilst anaphase K-fibers depolymerise for extended periods and high coherence is expected. However, we do observe a fraction of KTs undergoing transient reversals of the usual poleward motion during anaphase, Fig Z in S1 Appendix. These reversals were reported in previous studies, [5], whilst metaphase-like chromosome oscillations have been shown to persist into anaphase upon inhibition of protein dephosphorylation, [74]. The cause of these reversals is not understood, but suggests that coherence of microtubules within anaphase K-fibers may be dependent on factors that are perturbed under nocodazole washout, potentially the efficacy of dephosphorylation.

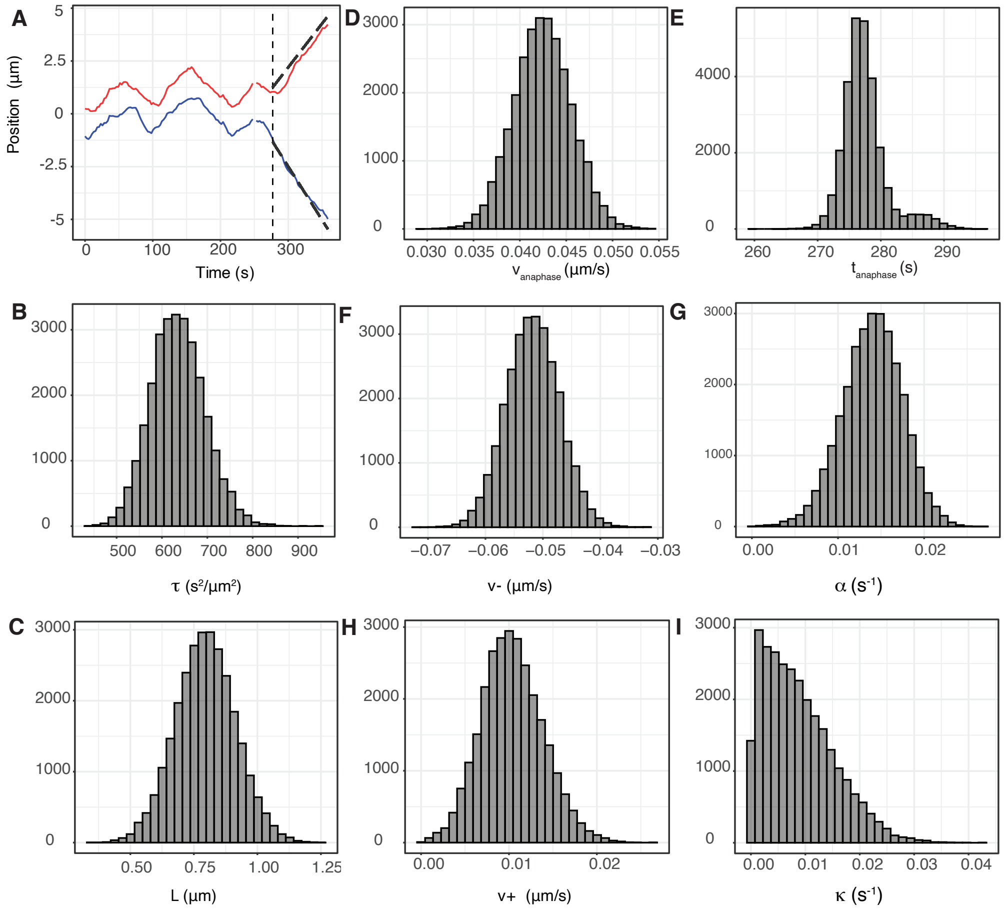

Fig AA. Single trajectory metaphase-anaphase model inference showing parameter estimates and trajectory annotation (DMSO treated cell). **A** Observed KT sister pair trajectory. **B-I** Marginal posterior distribution of the biophysical parameters for the trajectory data in A using the metaphase-anaphase model. Note: v_-, v_+ and $v_{anaphase}$ are averaged over the two sisters.

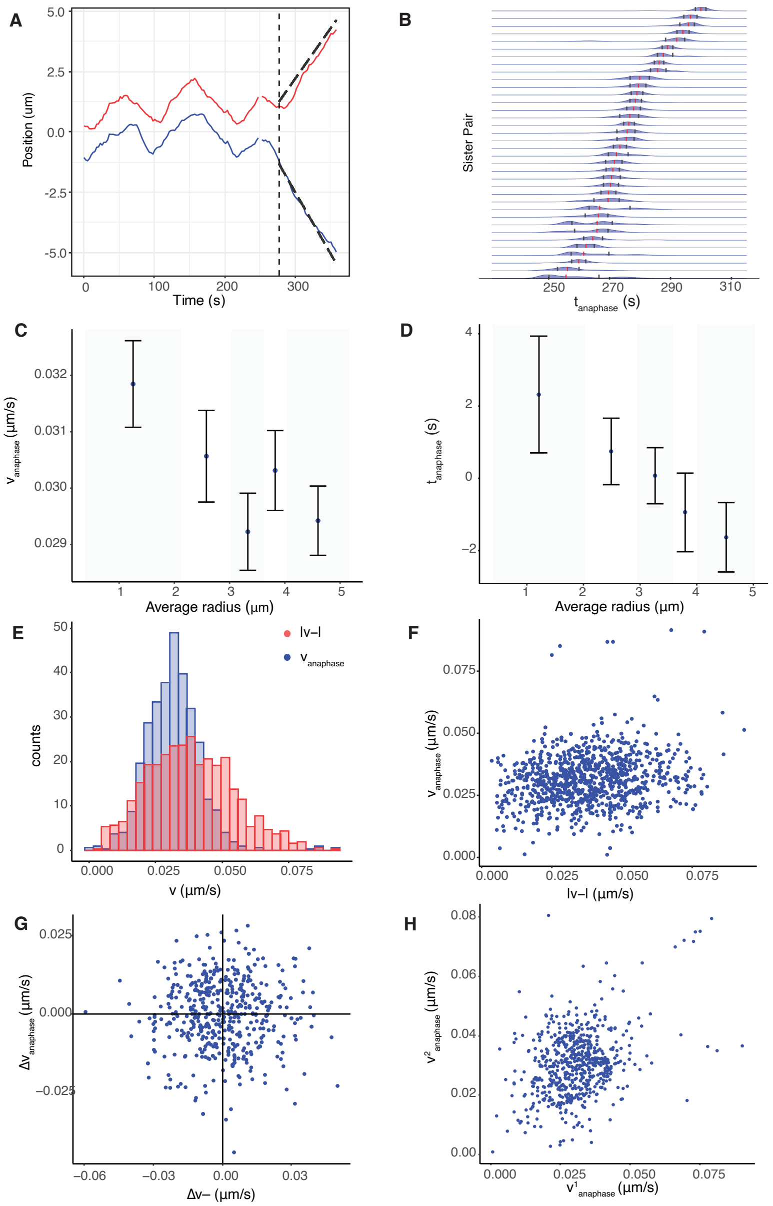

Fig AB. Anaphase K-fibers are dynamically homogeneous across the MPP despite variation in anaphase initiation (DMSO). **A** Example of the anaphase inference on a KT sister pair trajectory with anaphase event indicated with vertical line, and dashed lines above/below anaphase trajectory have gradients of v_a^k. **B** Anaphase timing of KT pairs in an individual cell. Red dots denote the distribution median and blue dots define the interquartile range. **C/D** Spatial biophysical parameter trends across the metaphase plate for anaphase speed/anaphase timings (relative to the median anaphase time per cell). The radial position of the KT pairs refers to their position during metaphase. The correlation between anaphase speed and anaphase timings is 0.247 ($p_{Corr} < 10^{-9}$). **E** Estimated densities of $v_{anaphase}$ (blue) and pulling force (red), (normalised by the drag coefficient). **F** Correlation of anaphase speed and absolute v_- (correlation $= 0.174$, $p_{MW} < 10^{-15}$). **G** Difference of $v^1_{anaphase}$ and $v^2_{anaphase}$ versus v^1_- and v^2_- only on asymmetric (on v_-) sister pairs. No statistically significant correlation is observed. **H** Correlation of anaphase speeds for sister 1 and sister 2 (correlation $= 0.386$, $p_{Corr} < 10^{-15}$). Figures are based on a subset of KT pair trajectories ($N = 611$ from 26 cells cultured in DMSO), which exhibited no reversals during anaphase (visual inspection), had adequate coverage of anaphase.

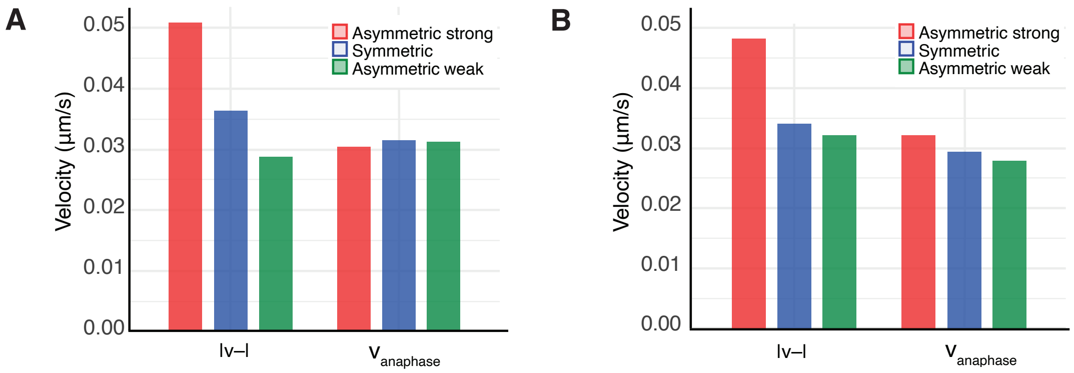

Fig AC. Comparison of averaged median speeds v_- and $v_{anaphase}$ for pulling strength groups on **A** DMSO ($N = 611$) and **B** nocodazole washout ($N = 767$) treated cells, (restricted to KT pairs with no reversals).

E Additional Models

Changepoint model

A simple changepoint model [90] to assess the time of anaphase assumes that the intersister distance between a kinetochore pair is constant in metaphase, and increases linearly in time during anaphase. If d_t is the 1D intersister distance (in the x direction perpendicular to the metaphase plate) between kinetochore sisters, then at time point t_i

$$d_{t_i} = \begin{cases} a_1 + \varepsilon_i \text{ for } t_i < t_A, \\ a_2 + b_2 t_i + \varepsilon_i \text{ for } t_i \geq t_A, \end{cases}$$

with the condition that $a_1 = a_2 + b_2 t_A$. Since this model is simpler than the biophysical model, a uniform prior can be used for t_A and weakly informative priors for other parameters.

Modelling joint pulling/pushing strength of K-fibers: a multivariate Normal mixture model analysis

We addressed if there is evidence of s specific asymmetric subpopulation. The v_+^k, v_-^k parameters are not independent (testing for independence on the full asymmetric model, using all KTs: $p_{Hoef} < 10^{-18}$, $p_{DC} < 10^{-4}$, $p_{Corr} < 10^{-5}$), with a significant correlation between pushing (v_+) and pulling (v_-) forces, Fig 5D of the main text. Therefore we modelled the joint empirical distribution of v_-, v_+ by fitting a mixture of multivariate Gaussians.

The multivariate Normal (Gaussian) model is a generalisation of the univariate normal model

$$\mathcal{N}_d\left(x \mid \mu, \Sigma\right) = \frac{1}{(2\pi)^{D/2}} \frac{1}{|\Sigma|^{1/2}} exp\{-\frac{1}{2}(x-\mu)^T \Sigma^{-1} \left(x-\mu\right)\}$$

with $\mu \in \mathcal{R}^d$, the location vector and $\Sigma \in \mathcal{R}^{d \times d}$, the covariance matrix. We assume that v_- and v_+ follow a multivariate normal distribution (allowing for v_- and v_+ to be dependent). Moving a step further, we have found that the model which explains the 2-dimensional distribution of pulling and pushing forces, is a 3-component multivariate normal mixture model which is defined as a linear superposition of the aforementioned multivariate normal

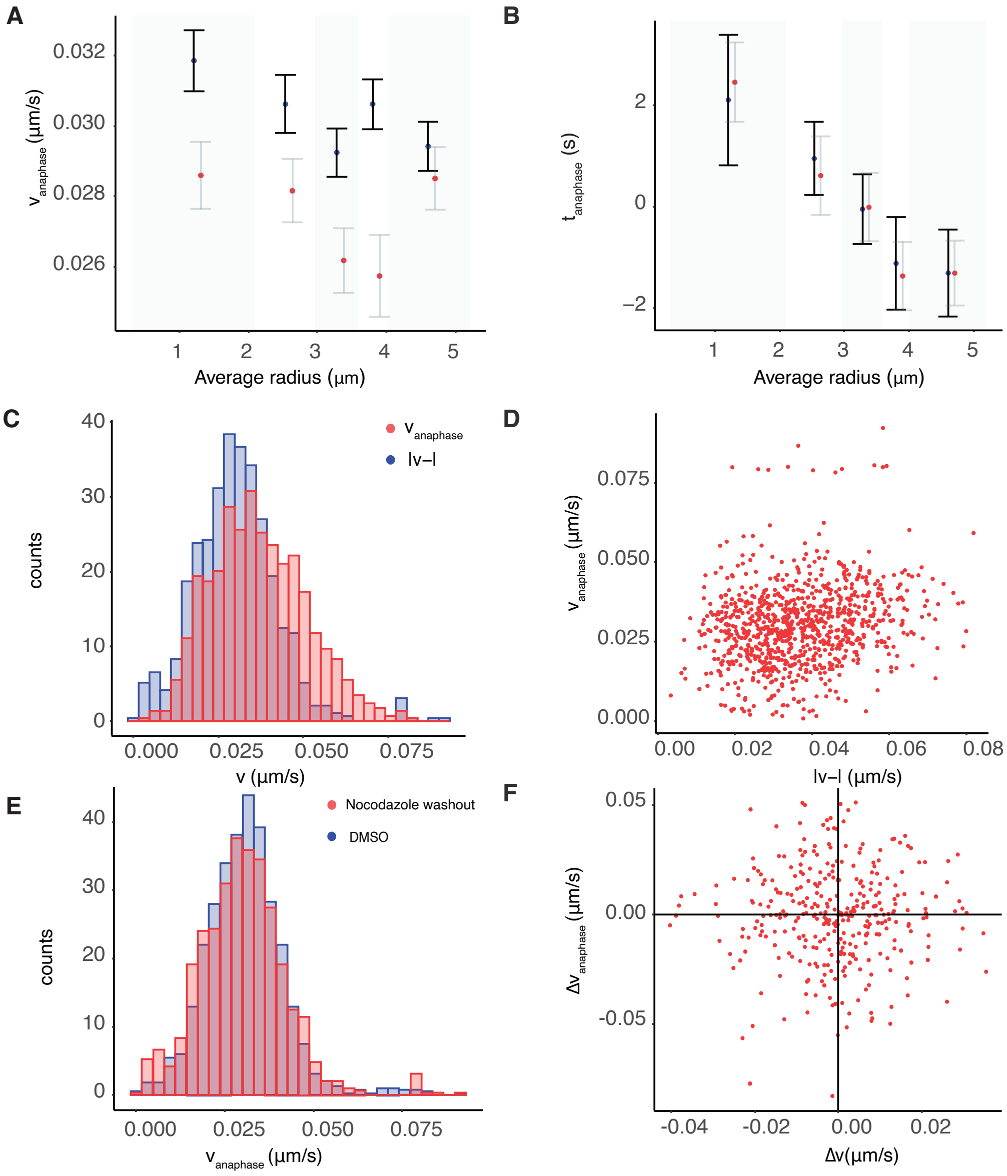

Fig AD. Homogeneity of anaphase K-fibers in DMSO cultured and nocodazole washout cells. **A/B** Spatial biophysical parameter trends across the metaphase plate for anaphase speed/anaphase timings for DMSO (blue) and nocodazole washout (red) treated cells. Data are partitioned into 5 equisized groups, defined by their radial positions, *i.e.*, $[0, 2.03], (2.03, 2.95], (2.95, 3.58], (3.580, 4.01], (4.01, 5.03]$. The estimated correlation in nocodazole washout treated cells between anaphase speed and relative anaphase timings is 0.125 ($p_{Corr} < 10^{-4}$). **C:F** Analysis based on nocodazole washout treated cells. **C** Estimated densities of $v_{anaphase}$ (red) and pulling force (blue) for nocodazole washout treated cells. **D** Correlation of anaphase speed and absolute v_- (estimated correlation $= 0.174$, $p_{Corr} < 10^{-15}$). **E** $v_{anaphase}$ for DMSO (blue) and nocodazole washout treated cells. Distributions are not statistically different ($p_{MW} = 0.165$), with the variance of $v_{anaphase}$ nocodazole washout pairs being greater ($p_{Ftest} < 10^{-4}$). **F** Difference of $v^1_{anaphase}$ and $v^2_{anaphase}$ versus v^1_- and v^2_- only on asymmetric (on v_-) sister pairs, for nocodazole washout treated cells. No statistically significant correlation is observed. Inference is based on 456 pairs across 30 nocodazole treated cells.

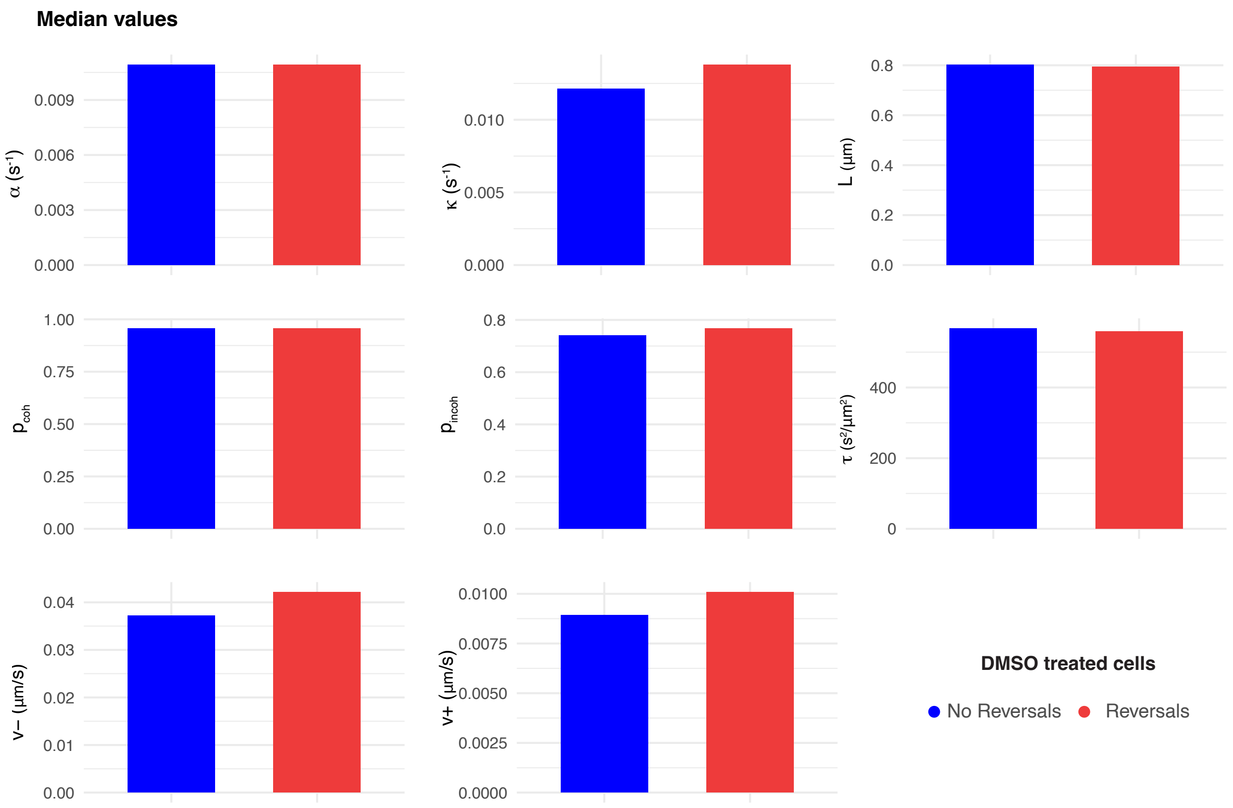

Fig AE. Bar plots of the median of the posterior median estimates of the trajectories with reversal events (red) and no reversal events (blue) during anaphase for DMSO treated cells. As shown in Table L, the distributions of v_-, v_+ and L are significantly different.

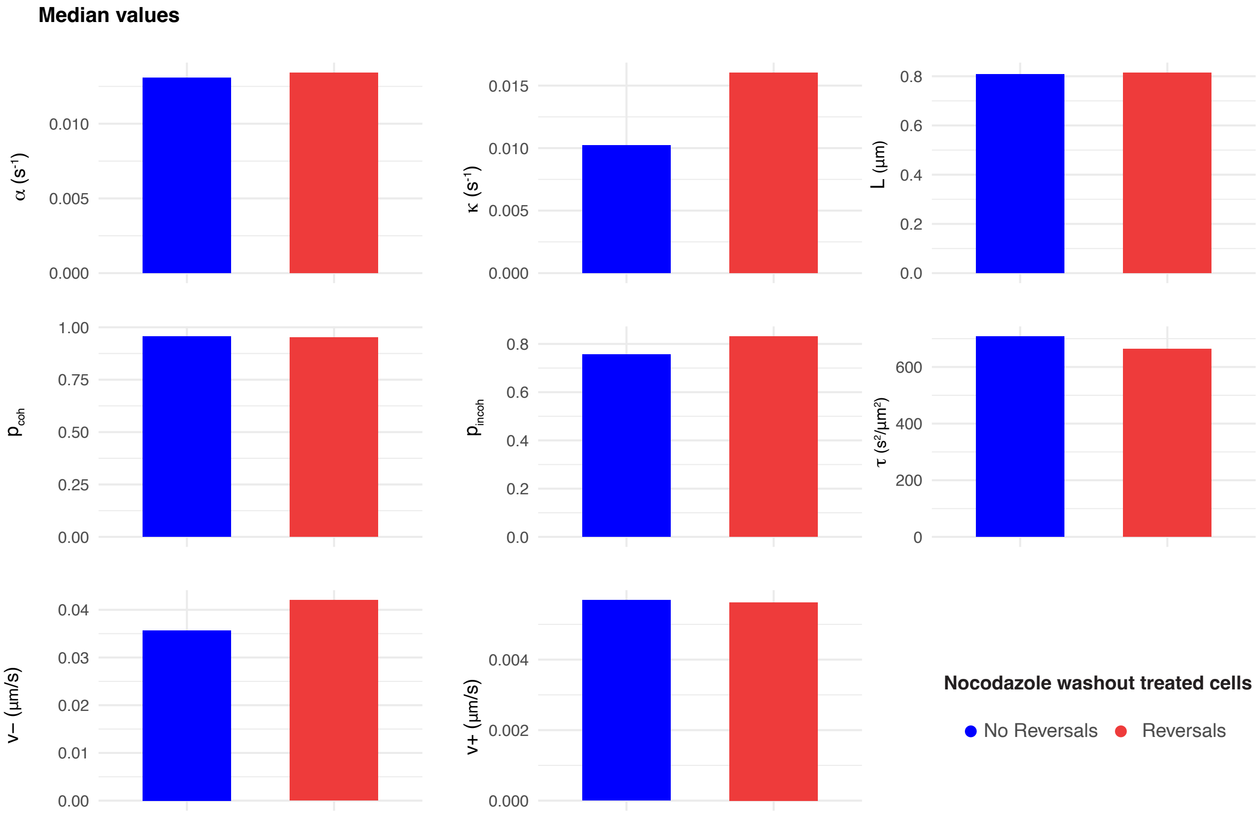

Fig AF. Bar plots of the median of the posterior median estimates of the trajectories with reversal events (red) and no reversal events (blue) during anaphase for nocodazole washout treated cells. As shown in Table L, the distributions of all parameters but v_+ and α are significantly different.

model, *i.e.*, v_- and v_+ have the following distribution,

$$f(x) = \sum_{k=1}^{K} \pi_k \mathcal{N}(x \mid \mu_k, \Sigma_k) \quad \text{subject to} \quad \Sigma_{k=1}^{K} \pi_k = 1$$

with $k = 3$, the number of Gaussian components, $\pi_1 ... \pi_k$, the mixture weights of the components, $\mu = \mu_1 ... \mu_k$, a vector containing the mean of each component and $\Sigma_1 ... \Sigma_k$, the covariance matrices of the components.

This model fitted the data extremely well, the QQ plot in fact indicating that there are no extremal subpopulations with large, or small, $|v_-|$, Fig AGB. Hence, this suggests that sister asymmetry is caused by natural variation of the K-fibres within a cell.

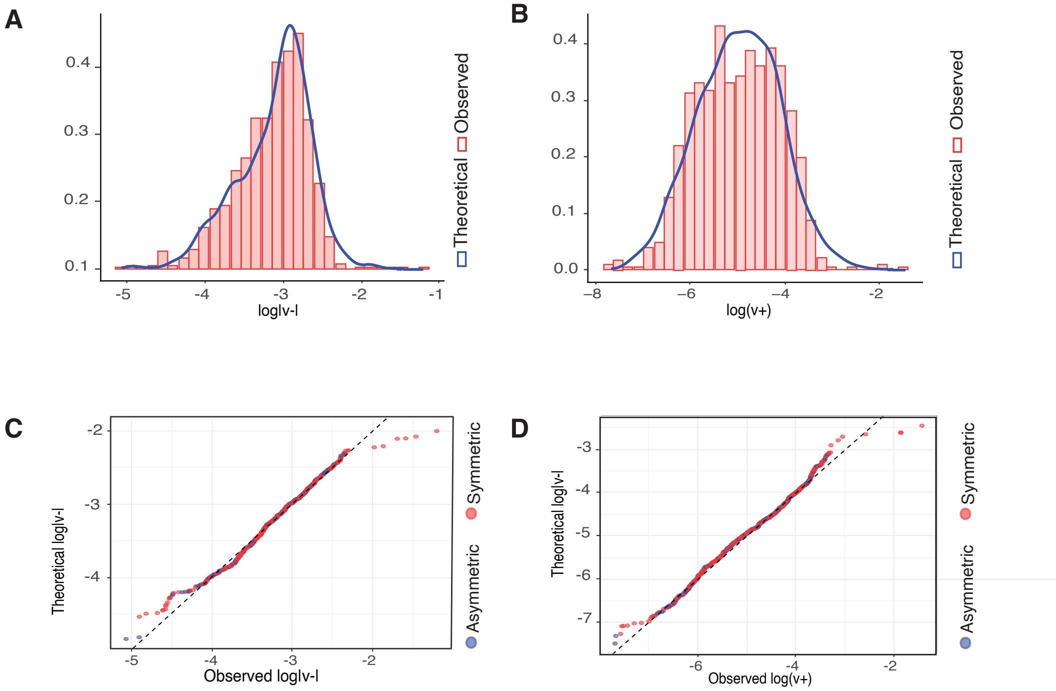

Fig AG. Mixture model of $v_\pm$ joint distribution. **A,B** Histograms of the estimated (red) and theoretical (blue) **A** $\log |v_-|$ and **B** $\log(v_+)$ of 3−component multivariate normal mixture model. **C,D** QQ plots for 3-component multivariate mixture model showing excellent fit.

References

[1] Mitchison T, Kirschner M. Dynamic instability of microtubule growth. Nature. 1984;312(5991). doi:10.1038/312237a0.

[2] Kiewisz R, Fabig G, Conway W, Baum D, Needleman D, Müller-Reichert T. Three-dimensional structure of kinetochore-fibers in human mitotic spindles. eLife. 2022;11:e75459. doi:10.7554/eLife.75459.

[3] Rago F, Cheeseman IM. The functions and consequences of force at kinetochores. Journal of Cell Biology. 2013;200(5):557–565.

[4] McAinsh AD, Marston AL. The Four Causes: The Functional Architecture of Centromeres and Kinetochores. Annual Review of Genetics. 2022;56(Volume 56, 2022):279–314. doi:https://doi.org/10.1146/annurev-genet-072820-034559.

[5] Skibbens RV, Skeen VP, Salmon ED. Directional Instability of Kinetochore Motility during Chromosome Congression and Segregation in Mitotic Newt Lung Cells : A Push-Pull Mechanism. Journal of Cell Biology. 1993;122(4).

[6] Wan X, Cimini D, Cameron LA, Salmon E. The coupling between sister kinetochore directional instability and oscillations in centromere stretch in metaphase PtK1 cells. Molecular Biology of the Cell. 2012;23(6):1035–1046.

[7] Jaqaman K, King EM, Amaro AC, Winter JR, Dorn JF, Elliott HL, et al. Kinetochore alignment within the metaphase plate is regulated by centromere stiffness and microtubule depolymerases. Journal of Cell Biology. 2010;188(5):665–679.

[8] Burroughs NJ, Harry EF, McAinsh AD. Super-resolution kinetochore tracking reveals the mechanisms of human sister kinetochore directional switching. Elife. 2015;4:e09500.

[9] Dick AE, Gerlich DW. Kinetic framework of spindle assembly checkpoint signalling. Nature Cell Biology. 2013;15(11):1370–1377. doi:10.1038/ncb2842.

[10] Tan CH, Gasic I, Huber-Reggi SP, Dudka D, Barisic M, Maiato H, et al. The equatorial position of the metaphase plate ensures symmetric cell divisions. eLife. 2015;4:e05124. doi:10.7554/eLife.05124.

[11] Cimini D, Howell B, Maddox P, Khodjakov A, Degrassi F, Salmon E. Merotelic kinetochore orientation is a major mechanism of aneuploidy in mitotic mammalian tissue cells. Journal of Cell Biology. 2001;153(3):517–528.

[12] Smith CA, McAinsh AD, Burroughs NJ. Human kinetochores are swivel joints that mediate microtubule attachments. Elife. 2016;5:e16159.

[13] Matos I, Pereira AJ, Lince-Faria M, Cameron LA, Salmon ED, Maiato H. Synchronizing chromosome segregation by flux-dependent force equalization at kinetochores. Journal of Cell Biology. 2009;186(1):11–26.

[14] Iemura K, Natsume T, Maehara K, Kanemaki MT, Tanaka K. Chromosome oscillation promotes Aurora A–dependent Hec1 phosphorylation and mitotic fidelity. Journal of Cell Biology. 2021;220(7):e202006116.

[15] Iemura K, Yoshizaki Y, Kuniyasu K, Tanaka K. Attenuated Chromosome Oscillation as a Cause of Chromosomal Instability in Cancer Cells. Cancers. 2021;13(18). doi:10.3390/cancers13184531.

[16] Harasymiw LA, Tank D, McClellan M, Panigrahy N, Gardner MK. Centromere mechanical maturation during mammalian cell mitosis. Nature communications. 2019;10(1):1–21.

[17] Risteski P, Božan D, Jagrić M, Bosilj A, Pavin N, Tolić IM. Length-dependent poleward flux of sister kinetochore fibers promotes chromosome alignment. Cell Rep. 2022;40(5):111169. doi:10.1016/j.celrep.2022.111169.

[18] Armond JW, Harry EF, McAinsh AD, Burroughs NJ. Inferring the forces controlling metaphase kinetochore oscillations by reverse engineering system dynamics. PLoS Computational Biology. 2015;11(11):e1004607.

[19] Civelekoglu-Scholey G, He B, Shen M, Wan X, Roscioli E, Bowden B, et al. Dynamic bonds and polar ejection force distribution explain kinetochore oscillations in PtK1 cells. Journal of Cell Biology. 2013;201(4):577–593.

[20] Vladimirou E, Mchedlishvili N, Gasic I, Armond JW, Samora CP, Meraldi P, et al. Nonautonomous movement of chromosomes in mitosis. Developmental Cell. 2013;27(1):60–71.

[21] Elting MW, Prakash M, Udy DB, Dumont S. Mapping load-bearing in the mammalian spindle reveals local kinetochore fiber anchorage that provides mechanical isolation and redundancy. Current Biology. 2017;27(14):2112–2122.

[22] Vladimirou E, Harry E, Burroughs N, McAinsh AD. Springs, clutches and motors: driving forward kinetochore mechanism by modelling. Chromosome Research. 2011;19(3):409–421. doi:10.1007/s10577-011-9191-x.

[23] Civelekoglu-Scholey G, Cimini D. Modelling chromosome dynamics in mitosis: A historical perspective on models of metaphase and anaphase in eukaryotic cells. Interface Focus. 2014;4(3).

[24] Hill TL. Theoretical problems related to the attachment of microtubules to kinetochores. Cell Biology. 1985;82:4404–4408.

[25] Joglekar AP, Hunt AJ. A simple, mechanistic model for directional instability during mitotic chromosome movements. Biophysical Journal. 2002;83(1):42–58.

[26] Civelekoglu-Scholey G, Sharp DJ, Mogilner A, Scholey JM. Model of Chromosome Motility in Drosophila Embryos : Adaptation of a General Mechanism for Rapid Mitosis. Biophysical Journal. 2006;90(11):3966–3982. doi:10.1529/biophysj.105.078691.

[27] Banigan EJ, Chiou KK, Ballister ER, Mayo AM, Lampson MA, Liu AJ. Minimal model for collective kinetochore–microtubule dynamics. Proceedings of the National Academy of Sciences. 2015;112(41):12699–12704.

[28] Schwietert F, Kierfeld J. Bistability and oscillations in cooperative microtubule and kinetochore dynamics in the mitotic spindle. New Journal of Physics. 2020;22(5):053008.

[29] Medina MAC, Iemura K, Kimura A, Tanaka K. A mathematical model of kinetochore-microtubule attachment regulated by Aurora A activity gradient describes chromosome oscillation and correction of erroneous attachments. Biomedical Research. 2021;42(5):203–219. doi:10.2220/biomedres.42.203.

[30] Kajtez J, Solomatina A, Novak M, Polak B, Vukušić K, Rüdiger J, et al. Overlap microtubules link sister k-fibres and balance the forces on bi-oriented kinetochores. Nature Communications. 2016;7(1):1–11.

[31] Miles CE, Zhu J, Mogilner A. Mechanical Torque Promotes Bipolarity of the Mitotic Spindle Through Multi-centrosomal Clustering. Bulletin of Mathematical Biology. 2022;84(2):29. doi:10.1007/s11538-021-00985-2.

[32] Holy TE, Leibler S. Dynamic instability of microtubules as an efficient way to search in space. Proc Natl Acad Sci U S A. 1994;91(12):5682–5. doi:10.1073/pnas.91.12.5682.

[33] Liu J, Desai A, Josȩ Onuchic N, Hwa T. An integrated mechanobiochemical feedback mechanism describes chromosome motility from prometaphase to anaphase in mitosis. Proceedings of the National Academy of Sciences. 2008;105(37):13752–13757. doi:10.1073/pnas.0807007105.

[34] Mogilner A, Wollman R, Civelekoglu-Scholey G, Scholey J. Modeling mitosis. Trends in Cell Biology. 2006;16(2):88–96.

[35] Loughlin R, Heald R, Nédélec F. A computational model predicts Xenopus meiotic spindle organization. Journal of Cell Biology. 2010;191(7):1239–1249. doi:10.1083/jcb.201006076.

[36] Zaytsev AV, Grishchuk EL. Basic mechanism for biorientation of mitotic chromosomes is provided by the kinetochore geometry and indiscriminate turnover of kinetochore microtubules. Molecular biology of the cell. 2015;26(22):3985–3998.

[37] Blackwell R, Sweezy-Schindler O, Edelmaier C, Gergely ZR, Flynn PJ, Montes S, et al. Contributions of microtubule dynamic instability and rotational diffusion to kinetochore capture. Biophysical Journal. 2017;112(3):552–563.

[38] Kliuchnikov E, Marx KA, Mogilner A, Barsegov V. Interrelated effects of chromosome size, mechanics, number, location-orientation and polar ejection force on the spindle accuracy: a 3D computational study. Mol Biol Cell. 2023;34(6):ar57. doi:10.1091/mbc.E22-11-0507.

[39] Chatterjee S, Sarkar A, Zhu J, Khodjakov A, Mogilner A, Paul R. Mechanics of Multicentrosomal Clustering in Bipolar Mitotic Spindles. Biophys J. 2020;119(2):434–447. doi:10.1016/j.bpj.2020.06.004.

[40] Li X, Bloomfield M, Bridgeland A, Cimini D, Chen J. A fine balance among key biophysical factors is required for recovery of bipolar mitotic spindle from monopolar and multipolar abnormalities. Mol Biol Cell. 2023;34(9):ar90. doi:10.1091/mbc.E22-10-0485.

[41] Gay G, Courtheoux T, Reyes C, Tournier S, Gachet Y. A stochastic model of kinetochore-microtubule attachment accurately describes fission yeast chromosome segregation. J Cell Biol. 2012;196(6):757–74. doi:10.1083/jcb.201107124.

[42] Kass RE, Raftery AE. Bayes factors. Journal of the American Statistical Association. 1995;90(430):773–795.

[43] Daniyan A, Inchingolo AV, McAinsh A, Burroughs N. Enhanced Kinetochore Detection During Mitotic Human Cell Division using CFAR. In: 2024 27th International Conference on Information Fusion (FUSION); 2024. p. 1–7.

[44] Harrison JU, Sen O, McAinsh AD, Burroughs NJ. Kinetochore tracking in 3D from lattice light sheet imaging data with KiT. Bioinformatics. 2022;.

[45] Roscioli E, Germanova TE, Smith CA, Embacher PA, Erent M, Thompson AI, et al. Ensemble-level organization of human kinetochores and evidence for distinct tension and attachment sensors. Cell Reports. 2020;31(4):107535.

[46] Dudka D, Castrogiovanni C, Liaudet N, Vassal H, Meraldi P. Spindle-length-dependent HURP localization allows centrosomes to control kinetochore-fiber plus-end dynamics. Current Biology. 2019;29(21):3563–3578.

[47] Stan Development Team. Stan Modeling Language Users Guide and Reference Manual, version 2.26.24; 2024. Available from: `https://mc-stan.org`.

[48] Carpenter B, Gelman A, Hoffman MD, Lee D, Goodrich B, Betancourt M, et al. Stan: a probabilistic programming language. Journal of Statistical Software. 2017;76(1):1–32.

[49] Betancourt M, Girolami M. Hamiltonian Monte Carlo for hierarchical models. Current trends in Bayesian methodology with applications. 2015;79(30):2–4.

[50] Neal R. MCMC Using Hamiltonian Dynamics. In: Brooks S, Gelman A, Jones GL, Meng XL, editors. Handbook of Markov Chain Monte Carlo. Chapman and Hall/CRC; 2011. p. 116–162.

[51] Hines KE, Middendorf TR, Aldrich RW. Determination of parameter identifiability in nonlinear biophysical models: A Bayesian approach. The Journal of General Physiology. 2014;143(3):401–416.

[52] Browning AP, Warne DJ, Burrage K, Baker RE, Simpson MJ. Identifiability analysis for stochastic differential equation models in systems biology. Journal of the Royal Society Interface. 2020;17(173):20200652.

[53] Gronau QF, Singmann H, Wagenmakers EJ. bridgesampling: An R Package for Estimating Normalizing Constants. Journal of Statistical Software. 2020;92(10):1–29. doi:10.18637/jss.v092.i10.

[54] Kass RE, Raftery AE. Bayes Factors. Journal of the American Statistical Association. 1995;90(430):773–795. doi:10.1080/01621459.1995.10476572.

[55] Gasic I, Nerurkar P, Meraldi P. Centrosome age regulates kinetochore–microtubule stability and biases chromosome mis-segregation. eLife. 2015;4:e07909. doi:10.7554/eLife.07909.

[56] Mosgöller W, Leitch AR, Brown JKM, Heslop-Harrison JS. Chromosome arrangements in human fibroblasts at mitosis. Human Genetics. 1991;88(1):27 – 33. doi:10.1007/BF00204924.

[57] Booth DG, Beckett AJ, Molina O, Samejima I, Masumoto H, Kouprina N, et al. 3D-CLEM Reveals that a Major Portion of Mitotic Chromosomes Is Not Chromatin. Molecular Cell. 2016;64(4):790–802. doi:10.1016/j.molcel.2016.10.009.

[58] Embacher PA, Germanova TE, Roscioli E, McAinsh AD, Burroughs NJ. Bayesian inference of multi-point macromolecular architecture mixtures at nanometre resolution. PLOS Computational Biology. 2022;18(12):1–34. doi:10.1371/journal.pcbi.1010765.

[59] Tulu US, Fagerstrom C, Ferenz NP, Wadsworth P. Molecular requirements for kinetochore-associated microtubule formation in mammalian cells. Curr Biol. 2006;16(5):536–41. doi:10.1016/j.cub.2006.01.060.

[60] Worrall JT, Tamura N, Mazzagatti A, Shaikh N, van Lingen T, Bakker B, et al. Non-random mis-segregation of human chromosomes. Cell Reports. 2018;23(11):3366–3380.

[61] Sen O, Harrison JU, Burroughs NJ, McAinsh AD. Kinetochore life histories reveal an Aurora-B-dependent error correction mechanism in anaphase. Developmental Cell. 2021;.

[62] Murtagh F, Contreras P. Algorithms for hierarchical clustering: an overview. WIREs Data Mining and Knowledge Discovery. 2012;2(1):86–97. doi:https://doi.org/10.1002/widm.53.

[63] Montero P, Vilar JA. TSclust: An R Package for Time Series Clustering. Journal of Statistical Software. 2014;62(1):1–43.

[64] Ke K, Cheng J, Hunt AJ. The distribution of polar ejection forces determines the amplitude of chromosome directional instability. Current Biology. 2009;19(10):807–815.

[65] Civelekoglu-Scholey G, He B, Shen M, Wan X, Roscioli E, Bowden B, et al. Dynamic bonds and polar ejection force distribution explain kinetochore oscillations in PtK1 cells. Journal of Cell Biology. 2013;201(4):577–593. doi:10.1083/jcb.201301022.

[66] Gnesotto FS, Mura F, Gladrow J, Broedersz CP. Broken detailed balance and non-equilibrium dynamics in living systems: a review. Reports on Progress in Physics. 2018;81(6):066601. doi:10.1088/1361-6633/aab3ed.

[67] Vukušić K, Buđa R, Bosilj A, Milas A, Pavin N, Tolić IM. Microtubule sliding within the bridging fiber pushes kinetochore fibers apart to segregate chromosomes. Developmental Cell. 2017;43(1):11–23.e6.

[68] Pavin N, Tolić IM. Mechanobiology of the mitotic spindle. Developmental Cell. 2020;.

[69] Dumont M, Gamba R, Gestraud P, Klaasen S, Worrall JT, De Vries SG, et al. Human chromosome-specific aneuploidy is influenced by DNA-dependent centromeric features. The EMBO journal. 2020;39(2):e102924.

[70] Drpic D, Almeida AC, Aguiar P, Renda F, Damas J, Lewin HA, et al. Chromosome segregation is biased by kinetochore size. Current Biology. 2018;28(9):1344–1356.

[71] Navarro AP, Cheeseman IM. Kinetochore assembly throughout the cell cycle. Seminars in Cell & Developmental Biology. 2021;117:62–74. doi:10.1016/j.semcdb.2021.03.008.

[72] Armond JW, Vladimirou E, Erent M, McAinsh AD, Burroughs NJ. Probing microtubule polymerisation state at single kinetochores during metaphase chromosome motion. Journal of Cell Science. 2015;128(10):1991–2001.

[73] Anjur-Dietrich MI, Kelleher CP, Needleman DJ. Mechanical mechanisms of chromosome segregation. Cells. 2021;10(2):465.

[74] Su KC, Barry Z, Schweizer N, Maiato H, Bathe M, Cheeseman IM. A regulatory switch alters chromosome motions at the metaphase-to-anaphase transition. Cell Reports. 2016;17(7):1728–1738.

[75] Vázquez-Novelle MD, Sansregret L, Dick AE, Smith CA, McAinsh AD, Gerlich DW, et al. Cdk1 inactivation terminates mitotic checkpoint surveillance and stabilizes kinetochore attachments in anaphase. Current Biology. 2014;24(6):638–645.

[76] Stephens AD, Haggerty RA, Vasquez PA, Vicci L, Snider CE, Shi F, et al. Pericentric chromatin loops function as a nonlinear spring in mitotic force balance. Journal of Cell Biology. 2013;200(6):757–772. doi:10.1083/jcb.201208163.

[77] Novak M, Polak B, Simunić J, Boban Z, Kuzmić B, Thomae AW, et al. The mitotic spindle is chiral due to torques within microtubule bundles. Nature Communications. 2018;9(1):1–10.

[78] Guerreiro A, De Sousa F, Liaudet N, Ivanova D, Eskat A, Meraldi P. WDR62 localizes katanin at spindle poles to ensure synchronous chromosome segregation. J Cell Biol. 2021;220(8). doi:10.1083/jcb.202007171.

[79] Jagrić M, Risteski P, Martinčić J, Milas A, Tolić IM. Optogenetic control of PRC1 reveals its role in chromosome alignment on the spindle by overlap length-dependent forces. eLife. 2021;10:e61170. doi:10.7554/eLife.61170.

[80] Shimamoto Y, Maeda YT, Ishiwata S, Libchaber AJ, Kapoor TM. Insights into the micromechanical properties of the metaphase spindle. Cell. 2011;145(7):1062–1074.

[81] Nazockdast E, Rahimian A, Needleman D, Shelley M. Cytoplasmic flows as signatures for the mechanics of mitotic positioning. Mol Biol Cell. 2017;28(23):3261–3270. doi:10.1091/mbc.E16-02-0108.

[82] Worrall JT, Tamura N, Mazzagatti A, Shaikh N, van Lingen T, Bakker B, et al. Non-random mis-segregation of human chromosomes. Cell Reports. 2018;23(11):3366–3380.

[83] Chen BC, Legant WR, Wang K, Shao L, Milkie DE, Davidson MW, et al. Lattice light-sheet microscopy: imaging molecules to embryos at high spatiotemporal resolution. Science. 2014;346(6208). doi:10.1126/science.1257998.

[84] Scott SL. Bayesian Methods for Hidden Markov Models. Journal of the American Statistical Association. 2002;97(457):337–351. doi:10.1198/016214502753479464.

[85] Stan Development Team. RStan: the R interface to Stan; 2024. Available from: `https://mc-stan.org/`.

[86] Meng XL, Wong WH. Simulating Ratios of Normalizing Constants via a Simple Identity: A Theoretical Exploration. Statistica Sinica. 1996;6(4):831–860.

[87] Gelman A, Rubin DB. Inference from iterative simulation using multiple sequences. Statistical Science. 1992;7(4):457–472.

[88] Vehtari A, Gelman A, Simpson D, Carpenter B, Bürkner PC. Rank-normalization, folding, and localization: An improved $\hat{R}$ for assessing convergence of MCMC. Bayesian Analysis. 2021;1(1):1–28.

[89] Holt LJ, Krutchinsky AN, Morgan DO. Positive feedback sharpens the anaphase switch. Nature. 2008;454(7202):353–357.

[90] Armond JW, Dale KL, Burroughs NJ, McAinsh AD, Vladimirou E. The dynamics of centromere motion through the metaphase-to-anaphase transition reveal a centromere separation order. BioRxiv. 2019; p. 582379.
