# Supplementary material for: Bayesian data driven modelling of kinetochore dynamics: Space-time organisation of the human metaphase plate
Source: PLoS Comput Biol. 2026 Jan 22;22(1):e1013884. doi: 10.1371/journal.pcbi.1013884 (PMC12935310; doi:10.1371/journal.pcbi.1013884)
Supplement: Attachment — Submitted filename: Comments_on_Koki_et_al_manuscript_2025.pdf [file pcbi.1013884.s002.pdf]

In their manuscript "Bayesian Data-Driven Modelling of Kinetochore Dynamics: Space-Time Organisation of the Human Metaphase Plate," Koki and colleagues investigate kinetochore dynamics in the human metaphase plate through experimental tracking and data-driven modeling. During mitosis, the cell self-organizes the spindle architecture—an intricate molecular machine responsible for ensuring accurate chromosome segregation into resulting daughter cells. High-fidelity segregation requires precise chromosome positioning at the spindle midzone and accurate chromosome connections to spindle poles via microtubules. This study explores the mechanics of the metaphase-to-anaphase transition by tracking kinetochore trajectories in experiments and analyzing underlying dynamics through fitting various force balance models. The research and methodological advancements presented in this paper are of significant interest to those studying the mechanics of spindle assembly. Overall, the manuscript is detailed, but certain sections are difficult to follow, particularly where trends from data-fitting are presented without a clear figure arrangement or sufficient and proper biological explanation. I suggest revisions to improve the storytelling and clarity of the key findings. Specific comments are listed below:

Major comments:

1. In Eq. 1, The authors denote $v_{\sigma_t^k}$ as the polymerization/depolymerization force. The model does not consider force generation due to microtubule sliding around the spindle equator. The authors should provide a perspective on how it would impact the model results.

2. By and large, all the tracks presented in Figure 1H are oscillatory. The authors should quantify the fraction of oscillatory vs non-oscillatory chromosomes within a cell and the cell population as a whole. How does the parameter landscape change when Eq. 1. is fitted to largely non-oscillatory chromosomes?

3. The spatial profile for PEF is chosen to be linear. What happens if other alternative profiles (such as exponentially diminishing with increasing pole-KT distance) are taken into account?

4. The model presented in Eq. 1 is essentially a 5-parameter model ($v_{\sigma_t^k}$, α, L, τ, κ). It would be insightful to examine the ranking of the parameters in terms of relative sensitivity to the fitted output. The same analysis would be valuable for the other models presented in the paper as well.

5. It is important to quantify the oscillation amplitude and time period of the chromosome oscillation during metaphase (e.g. Fig. 1H). Is it possible to identify and map specific biophysical parameter regimes based on oscillation characteristics and vice versa?

6. In Table 1, please include the inferred biophysical parameters for Hela cells as well for comparison.

7. In Figure 4H, the prior density plot for natural length L is plotted with red dashed lines on top of posterior histograms. Is there any particular reason why that has not been done for other histogram plots (for example, Figure 4D-G and Figure 4I)?

8. In line 238-242, the authors report the comparison of microtubule associated parameters between RPE1 and HeLa cells. While this is very informative, this follows an indirect approach of fitting the data into the proposed model followed by parameter extraction. A more direct approach

to validate this is to directly measure instability parameters of microtubules, poleward flux etc. in these two cells using live cell imaging.

9. The authors should clearly outline why they introduced asymmetry in section 2.4. The models without the asymmetry seem to explain the data to a reasonable degree.

10. In line 263-265 the authors report that "In RPE1 cells, 24.4% of sister kinetochores have significant asymmetry, with most, (42.6% of asymmetric pairs) preferring asymmetry in v−v+ whilst 87.7% have significant asymmetry in v−". What is the biological basis of this? It would be better to provide any direct experimental measurements of poleward flux to support this.

11. In line 279, the authors report that "pairs where a sister is both a stronger puller and pusher (respectively weaker puller and weaker pusher) are in a minority". It would be good to have a perspective on why this is so. Why can a stronger puller NOT transition into a stronger pusher or vice versa?

12. In line 325-328, the authors talk about the variation in centromeric stiffness and PEF with the distance of the kinetochores from the center of the metaphase plate (r). It would be interesting if authors could elaborate on why PEF is invariant with r. Although in line 602-616, the authors provide a perspective on why the stiffness changes with r, it is helpful to point to that in the results as well.

13. In line 381-383, some of the reported values do not match with the values mentioned in Table 5. The authors should also outline why the centromeric stiffness do not show any significant time dependence. Though the authors have cited Harasymiw et al. (2019), the above-mentioned point has not been addressed. Could the authors provide their perspective on this issue?

14. The authors should provide an estimate on the attachment statistics over the chromosome-microtubule attachments. Are they all largely amphitelic in the cases they analyzed? How does the incorrect attachments impact the models? What happens in Nocodazole washout cases where microtubules regrow after washout and progressively establish attachments anew? How significant is the cortical microtubule population during metaphase and anaphase? How would the spindle force balance change if cortical interactions are taken into consideration?

15. It seems to from the data presented here, the authors do not observe chromosome 'breathing' during oscillation. It would be interesting to include a discussion on that.

16. Although the section title in 2.9 is "Anaphase onset time is heterogeneous", nothing about the heterogeneity in discussed specifically in this section.

17. In line 491-492, the authors report that "although K-fibers are depolymerising in anaphase, we observed occasional reversals in individual KT trajectories, whereby a short reversal of direction occurs…". How did the authors quantify the reversal? How long did they scan through the anaphase for scoring reversal events? Does the reversal indicate the change of direction shown at > 750s in 11C? If so, the authors should point that in the figure for better clarity.

18. In line 452-455, the authors report that "Nocodazole washout treated cells have on average weaker K-fibers, specifically the magnitude of v+, v− were reduced by 37.5% (pMW < 10−15),

and 5.8% (pMW < 10−4) respectively, Appendix Table A3. They also have significantly reduced diffusive noise, (pMW < 10−15, the precision increased by 20%), a higher PEF (pMW < 10^{-10}, α increased by 15%) and the spring constant is reduced by 40% (pMW < 10−9).” Why did PEF increase here in contrast to the decrease in K-fiber flux? How did nocodazole treatment impact centromeric stiffness? The authors should provide an explanation. Additionally, the authors argue that in this scenario “fewer MTs are bundled into K-fibers, leaving a higher density of spindle MTs that would explain the increase in the strength of the PEF.” Do they have any experimental quantification to support this?

19. Throughout the paper, the authors present various estimates of $v_{+/-}$ as a measure of K-fiber mediated mechanical force. In reality, $v_{+/-}$ would depend on the interplay among microtubule dynamics at the plus ends, molecular motor activity and net poleward flux. The authors should include a discussion on that and highlight the limitations of individual models in a separate section.

20. In this study, the authors did not discuss the impact of various microtubule mediated forces on the structural stability of the spindle. Spindle positioning and the mechanical parameters of the spindle (pole-pole distance, rate of anaphase, poleward flux etc.) and additional factors like cell shape, size etc. are largely intertwined. The authors should provide additional in silico characterizations to that effect. Below I list some relevant articles the authors should take note of.

I. Li X, Bloomfield M, Bridgeland A, Cimini D, Chen J. A fine balance among key biophysical factors is required for recovery of bipolar mitotic spindle from monopolar and multipolar abnormalities. Mol Biol Cell. 2023 Aug 1;34(9):ar90. doi: 10.1091/mbc.E22-10-0485. Epub 2023 Jun 21. PMID: 37342878; PMCID: PMC10398891.

II. Mechanics of Multicentrosomal Clustering in Bipolar Mitotic Spindles Biophys J . 2020 Jul 21;119(2):434-447. doi: 10.1016/j.bpj.2020.06.004

III. Model for anaphase B: Role of three mitotic motors in a switch from poleward flux to spindle elongation PNAS, 101 (45) 15938-15943

21. Did the authors quantify changes in spindle length during metaphase? If variations in spindle length were observed over time during metaphase, how did this affect the model outcomes? Additionally, did spindle length vary on average across different cells? The authors should provide the statistics on this.

Minor comments:

1. In line 18, the authors should include the following reference:

Mitchison T, Kirschner M. Dynamic instability of microtubule growth. Nature. 1984 Nov 15-21;312(5991):237-42. doi: 10.1038/312237a0. PMID: 6504138.

2. In line 40, for better clarity, the authors should clearly illustrate what they are referring to as “active maturation of dynamics”.

3. Similarly, in line 49, the authors should clearly define what they allude to as ‘maturation of metaphase plate’ for general reader.

4. The authors cover a broad range of modeling studies in line 58-71. However, some key references should be discussed and included as suggested in the following:

I. Holy TE, Leibler S. Dynamic instability of microtubules as an efficient way to search in space. Proc Natl Acad Sci U S A. 1994 Jun 7;91(12):5682-5. doi: 10.1073/pnas.91.12.5682. PMID: 8202548; PMCID: PMC44060.

II. Chatterjee S, Sarkar A, Zhu J, Khodjakov A, Mogilner A, Paul R. Mechanics of Multicentrosomal Clustering in Bipolar Mitotic Spindles. Biophys J. 2020 Jul 21;119(2):434-447. doi: 10.1016/j.bpj.2020.06.004. Epub 2020 Jun 12. PMID: 32610087; PMCID: PMC7376091.

III. Banigan EJ, Chiou KK, Ballister ER, Mayo AM, Lampson MA, Liu AJ. Minimal model for collective kinetochore-microtubule dynamics. Proc Natl Acad Sci U S A. 2015 Oct 13;112(41):12699-704. doi: 10.1073/pnas.1513512112. Epub 2015 Sep 28.

IV. Interrelated effects of chromosome size, mechanics, number, location-orientation and polar ejection force on the spindle accuracy: a 3D computational study Evgenii Kliuchnikov, Kenneth A. Marx, Alexander Mogilner, and Valeri Barsegov Molecular Biology of the Cell 2023 34:6

V. Li X, Bloomfield M, Bridgeland A, Cimini D, Chen J. A fine balance among key biophysical factors is required for recovery of bipolar mitotic spindle from monopolar and multipolar abnormalities. Mol Biol Cell. 2023 Aug 1;34(9):ar90. doi: 10.1091/mbc.E22-10-0485. Epub 2023 Jun 21. PMID: 37342878; PMCID: PMC10398891.

VI. Loughlin R, Heald R, Nédélec F. A computational model predicts Xenopus meiotic spindle organization. J Cell Biol. 2010 Dec 27;191(7):1239-49. doi: 10.1083/jcb.201006076. Epub 2010 Dec 20. PMID: 21173114; PMCID: PMC3010074.

VII. Nazockdast E, Rahimian A, Needleman D, Shelley M. Cytoplasmic flows as signatures for the mechanics of mitotic positioning. Mol Biol Cell. 2017 Nov 7;28(23):3261-3270. doi: 10.1091/mbc.E16-02-0108. Epub 2017 Mar 22. PMID: 28331070; PMCID: PMC5687028.

5. Section 2.1 is more suitable in the methods section as it emphasizes more on the pipeline rather than the findings. I would suggest a revision for a crisp and concise results section overall.

6. Figures are poorly compiled overall in the paper. The font sizes are too small and inconsistent at places. Tics labels are also not properly assigned/incomplete in many figures. For example, in Figure 10C-10D, font sizes do not match. In Figure 10C, the ytics are incomplete. The axis tics should clearly show upper and lower limits in all figures wherever applicable. The physical units are also missing in x-label and y-label in many plots.

7. In Figure 12D, why does it show a decreasing trend with average radius? Ylabel physical unit (sec) is missing.

8. Line 559: Instead of “In addition, observed that”, it should be “In addition, we observed that”

In summary, the manuscript should be considered for publication after the revisions noted here. The quality of the data and the technical rigor are strong. However, the readability needs to be significantly improved in order to better connect the model predictions with the underlying

biological mechanisms. This is the key missing element in the current version. While the approach taken in this study is novel and rigorous, the data-driven presentation of model results in certain sections obscures the clarity of the biological underpinnings, making it a challenging read.
