# Supplementary material for: Bayesian data driven modelling of kinetochore dynamics: Space-time organisation of the human metaphase plate
Source: PLoS Comput Biol. 2026 Jan 22;22(1):e1013884. doi: 10.1371/journal.pcbi.1013884 (PMC12935310; doi:10.1371/journal.pcbi.1013884)
Supplement: Attachment — Submitted filename: Responses_to_Reviewers_comments.pdf [file pcbi.1013884.s003.pdf]

Response to Reviewers

Dear Editor and Reviewers,

Below, we provide detailed responses to each of the comments raised. We provide both a clean copy and a copy with marked changes. We feel these changes have substantially improved the manuscript, so we are grateful to the referees.

Response to Reviewer 1

Comment 1: *One major problem is that the paper is extremely dense and hard to read. At its core is the jargony and impenetrable lists of numerical procedures that will be of little use for a general reader. I strongly recommend replacing these long technical passages with a common-sensical description of ideas behind the methods. Just describe in simple words how do you obtain parameters from measured trajectories, and refer to technicalities in the appendices.*

Response: We have restructured the text to improve the readability, moved technical paragraphs to the Appendix and adding informative titles for each section/subsection. We have also attempted to make it more readable throughout.

Comment 2: *It is unclear what are the 18 models considered – needs clarification.*

Response: We have added a panel to Fig 3 to clarify the multiple models and their interrelationship. We also moved one model to the appendix (anaphase extension); model selection is in fact only on 17 models. We have adjusted the text accordingly.

Response to Reviewer 2

Major Comments

Comment 1: *In Eq. 1, The authors denote $v_{\sigma_t^k}$ as the polymerization/depolymerization force. The model does not consider force generation due to microtubule sliding around the spindle equator. The authors should provide a perspective on how it would impact the model results.*

Response: Thank you for this comment. We now refer to K-fiber pulling and pushing forces, composite forces with components from the plus end (de)polymerisation dynamics and the K-fibre flux. We discuss these components in the discussion.

Comment 2: *By and large, all the tracks presented in Figure 1H are oscillatory. The authors should quantify the fraction of oscillatory vs non-oscillatory chromosomes within a cell and the*

cell population as a whole. How does the parameter landscape change when Eq. 1. is fitted to largely non-oscillatory chromosomes?

Response: Thank you for this comment. Figure 1H was a representative cell. We have added a new section on analysis of the oscillations based on clustering of the ACF, that clusters KT pairs into various clusters in the main text in section 2.9 "Oscillations are heterogenous in quality and strength" and additional figures and tables in Appendix, Tables A8-11, Figures B20-B25. We show a breakdown into good and poor oscillators. In DMSO treated data 35.8% of chromosome pairs where bad oscillators and 23.2% weak oscillators. The main difference in the estimations of equation 1 are v_-, and p_{coh}, while in Nocodazole treated data 27.3% were bad oscillators and 34.6% were weak oscillators, with the main differences in v_-, v_+, α and p_{coh}, Appendix Tables 8,9.

Comment 3: *The spatial profile for PEF is chosen to be linear. What happens if other alternative profiles (such as exponentially diminishing with increasing pole-KT distance) are taken into account?*

Response: This is a very interesting question. However, we explored alternative spatial profiles for PEF (e.g., exponential), as this would require the development and analysis of an entirely new set of models. The fact that the spindle pole is far away from the KTs, during metaphase, makes the linear model for PEF a reasonable approach.

Comment 4: *The model presented in Eq. 1 is essentially a 5-parameter model ($v_{\sigma_t^j}, \alpha, L, \tau, \kappa$). It would be insightful to examine the ranking of the parameters in terms of relative sensitivity to the fitted output. The same analysis would be valuable for the other models presented in the paper as well.*

Response: It would indeed be interesting to determine the sensitivity of outputs such as period, amplitude and oscillation quality on parameters, but a Sobel sensitivity analysis is beyond the scope of this paper. The paper focuses on parameter fitting and data driven modelling of the metaphase oscillations, i.e., to determine which model is appropriate for analysis of oscillations, therefore such a sensitivity analysis addresses different questions.

Comment 5: *It is important to quantify the oscillation amplitude and time period of the chromosome oscillation during metaphase (e.g. Fig. 1H). Is it possible to identify and map specific biophysical parameter regimes based on oscillation characteristics and vice versa?*

Response: We have added an analysis of oscillation characteristics in the whole new Section 2.9, finding 5 clusters. We related those clusters to the heterogeneity in biophysical parameters, and conclude that oscillation quality is yet another aspect of variabilty, distinct from spatial heterogeneity.

Comment 6: *In Table 1, please include the inferred biophysical parameters for Hela cells as well for comparison.*

Response: The parameter estimates on HeLa cells (quoted from Armond et al., 2015) and the estimates in RPE1 cells are not directly comparable given the low kinetochore coverage in HeLa cells compared to the near complete tracking acheived here (as mentioned in text, due to different microscope, different flourophore (overexpressed) and lower performance tracking algorithm). Thus, placing estimates in table would be misleading as it isn't like for like. Note that we have revised the paragraph with the HeLa cells comparison.

Comment 7: *In Figure 4H, the prior density plot for natural length L is plotted with red dashed lines on top of posterior histograms. Is there any particular reason why that has not been done for other histogram plots (for example, Figure 4D-G and Figure 4I)?*

Response: It is only for the natural length L that we have imposed an informative prior. Plotting uninformative priors is unnecessary.

Comment 8: *In line 238-242, the authors report the comparison of microtubule associated parameters between RPE1 and HeLa cells. While this is very informative, this follows an indirect approach of fitting the data into the proposed model followed by parameter extraction. A more direct approach to validate this is to directly measure instability parameters of microtubules, poleward flux etc. in these two cells using live cell imaging.*

Response: We agree that a direct estimate of these parameters would be great. But sadly, techniques to do this on a single kinetochore basis do not exist.

Comment 9: *The authors should clearly outline why they introduced asymmetry in section 2.4. The models without the asymmetry seem to explain the data to a reasonable degree.*

Response: The spindles are not equal, with a young and old centrosome. Since centrosome age has impacts on MT stability, which cause half-spindle biases, it is not justifiable to assume KT dynamics is symmetric. This motivation is now added to the text. What we demonstrate is that asymmetry is both a natural consequence of parameter stochastic variability, and has consequences (transverse ordering of the MPP). The symmetric model may be adequate for some analyses, but will fail in others.

Comment 10: *In line 263-265 the authors report that "In RPE1 cells, 24.4% of sister kinetochores have significant asymmetry, with most, (42.6% of asymmetric pairs) preferring asymmetry in $v-v+$ whilst 87.7% have significant asymmetry in v-". What is the biological basis of this? It would be better to provide any direct experimental measurements of poleward flux to support this.*

Response: We provide evidence that this is due to random processes during assembly of the KT and K-fiber, so there is no asymmetric population as such as it is a continuum (the significantly asymmetric set is those KT pairs where the asymmetry is sufficiently large given the length of the time series, that determines parameter confidence). This is discussed in Discussion, bullet point 3, including a reference to the possibility that it is related to flux. New experiments are needed to identify the causes of asymmetry that are beyond the scope of this paper.

Comment 11: *In line 279, the authors report that "pairs where a sister is both a stronger puller and pusher (respectively weaker puller and weaker pusher) are in a minority". It would be good to have a perspective on why this is so. Why can a stronger puller NOT transition into a stronger pusher or vice versa?*

Response: We are reporting our observations on the correlations of pulling and pushing forces K-fiber, and discuss this signature in the context of possible explanations in the Discussion. We didn't test if the signature is time invariant. Additional experimental work is needed to unravel the cause, but this is beyond the scope of the present manuscript.

Comment 12: *In line 325-328, the authors talk about the variation in centromeric stiffness and PEF with the distance of the kinetochores from the center of the metaphase plate (r). It would be interesting if authors could elaborate on why PEF is invariant with r. Although in*

line 602-616, the authors provide a perspective on why the stiffness changes with r, it is helpful to point to that in the results as well.

Response: Since the spindle is a geometrically complex, heterogeneous object, an invariance in the centralising forces is open for explanation. We don't feel this warrants any more discussion as there are no obvious explanations to our knowledge, especially as the centralising force is a composite (3 components).

Comment 13: *In line 381-383, some of the reported values do not match with the values mentioned in Table 5. The authors should also outline why the centromeric stiffness do not show any significant time dependence. Though the authors have cited Harasymiw et al. (2019), the above-mentioned point has not been addressed. Could the authors provide their perspective on this issue?*

Response: We have have added lines in the text and in the caption explaining the different percentages. As regards time variation in the centromeric spring, all the literature reporting a change over mitosis compare prometaphase to metaphase (incl. Harasymiw et al); this is now emphasised in the introduction. There are no reports of intrametaphase changes as far as we are aware, so we don't feel the lack of a temporal dependence reported here warrants further discussion.

Comment 14: *The authors should provide an estimate on the attachment statistics over the chromosome-microtubule attachments. Are they all largely amphitelic in the cases they analyzed? How does the incorrect attachments impact the models? What happens in Nocodazole washout cases where microtubules regrow after washout and progressively establish attachments anew? How significant is the cortical microtubule population during metaphase and anaphase? How would the spindle force balance change if cortical interactions are taken into consideration?*

Response: The only way to distinguish attachments (amphitelic, merotelic, syntelic) is through higher resolution techniques such as EM or superresolution (SIM, STED), ideally in a correlative live fixed experiments, although these methods have their own issues. These are a lovely set of experiments but beyond the scope of the present manuscript. Since misattachment frequency is estimated to be low, even in nocodazole washout, a fraction of misattachments will not affect the results significantly as they are too rare.

As to the cortical (astral) microtubules, this is important for force balance and (re)orientation of the spindle, driven by forces from dynein and Eg5 motors primarily. We analyse KT dynamics which is independent of overall spindle dynamics since we use coordinates relative to the MPP and thus spindle rotation is irrelevant. In metaphase the inter spindle pole separation will also be approximately constant.

Comment 15: *It seems to from the data presented here, the authors do not observe chromosome 'breathing' during oscillation. It would be interesting to include a discussion on that.*

Response: Breathing oscillations are observed in RPE1 cells. We now include data to show these oscillations, Appendix Figures B6, B7.

Comment 16: *Although the section title in 2.9 is "Anaphase onset time is heterogeneous", nothing about the heterogeneity in discussed specifically in this section.*

Response: We have moved the section about anaphase from the main text to the Appendix D "Anaphase transition: modelling and analysis", to improve the readability and flow of the manuscript.

Comment 17: *In line 491-492, the authors report that "although K-fibers are depolymerising in anaphase, we observed occasional reversals in individual KT trajectories, whereby a short reversal of direction occurs... ". How did the authors quantify the reversal? How long did they scan through the anaphase for scoring reversal events? Does the reversal indicate the change of direction shown at ¿ 750s in 11C? If so, the authors should point that in the figure for better clarity*

Response: We identified the reversals by visual inspection of an expert. As we have moved the anaphase analysis to the appendix, please note that we have added arrows showing the reversal events in Appendix Figure D1C (previously as Figure 11C).

Comment 18: *In line 452-455, the authors report that "Nocodazole washout treated cells have on average weaker K-fibers, specifically the magnitude of $v+$, $v-$ were reduced by 37.5% ($pMW < 10^{-15}$), and 5.8% ($pMW < 10^{-4}$) respectively, Appendix Table A3. They also have significantly reduced diffusive noise, ($pMW < 10^{-15}$, the precision increased by 20%), a higher PEF ($pMW < 10^{-10}$, α increased by 15%) and the spring constant is reduced by 40% ($pMW < 10^{-9}$)." Why did PEF increase here in contrast to the decrease in K-fiber flux? How did nocodazole treatment impact centromeric stiffness? The authors should provide an explanation. Additionally, the authors argue that in this scenario "fewer MTs are bundled into K-fibers, leaving a higher density of spindle MTs that would explain the increase in the strength of the PEF." Do they have any experimental quantification to support this?*

Response: We have reported the results of our analysis on the DMSO and nocodazole washout treated cells, but can only hypothesise the mechanism. For instance, we have suggested a possible explanation of the PEF and K-fibre strength. The reduced spring constant may be due to spring nonlinearity (increasing spring 'constant' with stretch, [**?**]), so a decreased stretch gives a lower effective spring constant. However, we did not find evidence in the dynamics for nonlinear springs from the dynamics. Further work is therefore needed to validate this and understand the reason for the other changes.

Comment 19: *Throughout the paper, the authors present various estimates of $v+/-$ as a measure of K-fiber mediated mechanical force. In reality, $v+/-$ would depend on the interplay among microtubule dynamics at the plus ends, molecular motor activity and net poleward flux. The authors should include a discussion on that and highlight the limitations of individual models in a separate section.*

Response: Yes, v_+- are net forces, the combined effect of (de)polymerisation at the plus end, depolymerisation (flux) at the minus end and control through HURP. We have now defined centralising forces as a composite force, and discuss the various components in Introduction and Discussion.

Comment 20: *In this study, the authors did not discuss the impact of various microtubule mediated forces on the structural stability of the spindle. Spindle positioning and the mechanical parameters of the spindle (pole-pole distance, rate of anaphase, poleward flux etc.) and additional factors like cell shape, size etc. are largely intertwined. The authors should provide additional in silico characterizations to that effect. Below I list some relevant articles the authors should take note of.*

I. Li X, Bloomfield M, Bridgeland A, Cimini D, Chen J. A fine balance among key biophysical factors is required for recovery of bipolar mitotic spindle from monopolar and

multipolar abnormalities. Mol Biol Cell. 2023 Aug 1;34(9):ar90. doi: 10.1091/mbc.E22-10-0485. Epub 2023 Jun 21. PMID: 37342878; PMCID: PMC10398891.

II. Mechanics of Multicentrosomal Clustering in Bipolar Mitotic Spindles Biophys J . 2020 Jul 21;119(2):434-447. doi: 10.1016/j.bpj.2020.06.004
III. Model for anaphase B: Role of three mitotic motors in a switch from poleward flux to spindle elongation PNAS, 101 (45) 15938-15943

Response: Our data does not tell us anything about spindle stability so we cannot add any analysis. We disagree that a discussion of this is relevant.

Comment 21: *Did the authors quantify changes in spindle length during metaphase? If variations in spindle length were observed over time during metaphase, how did this affect the model outcomes? Additionally, did spindle length vary on average across different cells? The authors should provide the statistics on this.*

Response: We did not measure spindle length as unfortunately the cell line does not have a marker for the spindle. Thus, we could not see the location of the spindles.

Minor Comments

Comment 1: *In line 18, the authors should include the following reference:*
Mitchison T, Kirschner M. Dynamic instability of microtubule growth. Nature. 1984 Nov 15-21;312(5991):237-42. doi: 10.1038/312237a0. PMID: 6504138

Response: Reference added.

Comment 2: *In line 40, for better clarity, the authors should clearly illustrate what they are referring to as "active maturation of dynamics".*

Response: We now define maturation explicitly in Introduction, lines 34-39 (highlighted copy).

Comment 3: *Similarly, in line 49, the authors should clearly define what they allude to as 'maturation of metaphase plate' for general reader*

Response: Clarified.

Comment 4: *The authors cover a broad range of modeling studies in line 58-71. However, some key references should be discussed and included as suggested in the following:*
I. Holy TE, Leibler S. Dynamic instability of microtubules as an efficient way to search in space. Proc Natl Acad Sci U S A. 1994 Jun 7;91(12):5682-5. doi: 10.1073/pnas.91.12.5682. PMID: 8202548; PMCID: PMC44060.
II. Chatterjee S, Sarkar A, Zhu J, Khodjakov A, Mogilner A, Paul R. Mechanics of Multicentrosomal Clustering in Bipolar Mitotic Spindles. Biophys J. 2020 Jul 21;119(2):434-447. doi: 10.1016/j.bpj.2020.06.004. Epub 2020 Jun 12. PMID: 32610087; PMCID: PMC7376091.
III. Banigan EJ, Chiou KK, Ballister ER, Mayo AM, Lampson MA, Liu AJ. Minimal model for collective kinetochore-microtubule dynamics. Proc Natl Acad Sci U S A. 2015 Oct 13;112(41):12699-704. doi: 10.1073/pnas.1513512112. Epub 2015 Sep 28.
IV. Interrelated effects of chromosome size, mechanics, number, location-orientation and polar ejection force on the spindle accuracy: a 3D computational study Evgenii Kliuchnikov, Kenneth A. Marx, Alexander Mogilner, and Valeri Barsegov Molecular Biology of

the Cell 2023 34:6
V. Li X, Bloomfield M, Bridgeland A, Cimini D, Chen J. A fine balance among key biophysical factors is required for recovery of bipolar mitotic spindle from monopolar and multipolar abnormalities. Mol Biol Cell. 2023 Aug 1;34(9):ar90. doi: 10.1091/mbc.E22-10-0485. Epub 2023 Jun 21. PMID: 37342878; PMCID: PMC10398891.
VI. Loughlin R, Heald R, Nédélec F. A computational model predicts Xenopus meiotic spindle organization. J Cell Biol. 2010 Dec 27;191(7):1239-49. doi: 10.1083/jcb.201006076. Epub 2010 Dec 20. PMID: 21173114; PMCID: PMC3010074.
VII. Nazockdast E, Rahimian A, Needleman D, Shelley M. Cytoplasmic flows as signatures for the mechanics of mitotic positioning. Mol Biol Cell. 2017 Nov 7;28(23):3261-3270. doi: 10.1091/mbc.E16-02-0108. Epub 2017 Mar 22. PMID: 28331070; PMCID: PMC5687028.

Response: Thank you for pointing this out. We have included all except one of these references (Nazockdast et al) in that section, and also added references to metaphase oscillation models.

Comment 5: *Section 2.1 is more suitable in the methods section as it emphasizes more on the pipeline rather than the findings. I would suggest a revision for a crisp and concise results section overall.*

Response: We have revised 2.1 section.

Comment 6: *Figures are poorly compiled overall in the paper. The font sizes are too small and inconsistent at places. Tics labels are also not properly assigned/incomplete in many figures. For example, in Figure 10C-10D, font sizes do not match. In Figure 10C, the ytics are incomplete. The axis tics should clearly show upper and lower limits in all figures wherever applicable. The physical units are also missing in x-label and y-label in many plots.*

Response: Addressed.

Comment 7: *In Figure 12D, why does it show a decreasing trend with average radius? Ylabel physical unit (sec) is missing.*

Response: We have added the YLabel. This shows that peripheral KT pairs enter anaphase earlier. This figure is now in appendix.

Comment 8: *Line 559: Instead of "In addition, observed that", it should be "In addition, we observed that"*

Response: We have re-written almost all the Discussion.

Comment 9: *In summary, the manuscript should be considered for publication after the revisions noted here. The quality of the data and the technical rigor are strong. However, the readability needs to be significantly improved in order to better connect the model predictions with the underlying biological mechanisms. This is the key missing element in the current version. While the approach taken in this study is novel and rigorous, the data-driven presentation of model results in certain sections obscures the clarity of the biological underpinnings, making it a challenging read.*

Response: Thank you for your thorough reading of our manuscript. Based on your comments, as well as those from the other reviewers, we have made significant revisions to improve the clarity and readability of the paper. We believe these changes have

strengthened the presentation and better connect the model predictions with the underlying biological mechanisms. We hope that we have addressed all of your concerns satisfactorily.

Response to Reviewer 3

Major points

Comment 1: *The authors should include or at least discuss flux-driven kinetochore centering (Risteski, Cell Rep. 2022). Their main centering mechanism involves only the opposing polar ejection and pulling forces, but this description may not be complete.*

Response: We have now used centering forces as a composite for all forces that center the chromosomes to the metaphase plate, and discuss the components in the Discussion.

Comment 2: *Bridging microtubules are mentioned in the discussion as a possible cause of spatial variability in k-fiber-related parameters (pulling/pushing forces and noise precision), and the authors state that these properties adapt to the changing geometry at the metaphase plate periphery. A more detailed discussion is needed on how changing geometry propagates to k-fiber parameters.*

Response: This is discussed mainly in Discussion. A wider discussion, and modelling is outside the scope of the present manuscript.

Comment 3: *The authors should state more clearly what exactly is the advantage of using Bayesian inference over extracting such parameters from experiments in a more direct way.*

Response: Many of the variables with biophysical meaning can not be measured directly, but rather than estimated through biophysical reversed engineering models. Estimating the parameters of these constructed models can then be achieved though Bayesian modeling/inference as closed forms of the estimators do not exist. We have added some lines on this in Introduction, lines 61-81 (highlighted copy).

Comment 4: *In section C (Methodology), I do not find an expression (or iterative formula) for the likelihood of the model parameters conditioned on the data, only the likelihood of the data conditioned on the model parameters. Since the former is used to calculate the median of the parameters, explicitly stating its formula is mandatory*

Response: Our inference is based on the posterior distribution of the parameters. The posterior distribution is proportional to prior$\times$ likelihood – both of them given in methodology. We did not write the closed form of the posterior distribution, as it is not possible to have an analytic form but rather than estimate it using the Hamiltonian Monte Carlo algorithm. Therefore, the medians were calculated from the estimated posterior samples output.

Comment 5: *The authors should discuss how merotelic attachments enter their model, since, as they mentioned, there is an increase in merotelic attachments in cells treated with nocodazole washout.*

Response: We are unable to detect meroteli unfortunately, and the proportion of misattachments at metaphase in RPE1 cells, even in nocodazole washout is likely small. Hence we are unable to analyse or model merotelic attached pairs.

Comment 6: *There are no results anywhere in the manuscript for the angle theta between sister kinetochores. Is there a particular reason for omitting it? Also, no data about $p_i$coh and $p_c$oh are shown, neither in the main figures nor in the supplement.*

Response: We have added a supplementary Figure B6A to our main Figure 4 with the cosine angle plots for that particular KT pair. Regarding the transition probabilities between coherent and incoherent states, we report the values in Table 3, while we have plotted the posterior probabilities of being in each state Figure 4B and the LIDS/TIDS percentages in the main text (Paragraph 2.3) and the LIDS/TIDS plot, Figure 4C, as an indirect plots of coherent and incoherent probabilities. Also, we have plotted the coherent and incoherent probabilities in Appendix Figures B9,B24,B25.

Minor Comments

Comment 1: *It is not clear how the authors define the beginning of metaphase.*

Response: We haven't used the term "beginning of the metaphase", but rather than metaphase, mid-metaphase, defined by the time prior to anaphase onset, which can be easily defined (we have used a change point model to do this in our analysis, Appendix E.).

Comment 2: *I suggest moving Figures 1C, 1E, 1F and 1H to the supplement. The authors should describe whether Figure 1D shows data for a single cell or if they are pooled across multiple cells*

Response: Addressed.

Comment 3: *The authors should better explain in the introduction why they would even consider more complex models of kinetochore oscillations and how this fits into existing research by other groups.*

Response: Mitosis is an extremely complex and poorly understood mechanical process. Given the massive advances in microscopy, we are now able to observe it in greater detail. This demands utilising more advanced models and techniques in analysis of that data.

Comment 4: *The labels on some plots are too small (e.g. Figs. 6C and 6D). Figure 5E looks stretched. The labels on the y-axis of the histogram are missing (e.g. Fig. 5E, Figs. 6A-F, 7A,10A,12E). Some plots are missing units (e.g. Figs. 8C-E).*

Response: Thank you. We have increased the labels fonts and added units on all of them.

Comment 5: *In equation (1), sqrt(δt) should be replaced by its inverse.*

Response: Based on the Euler–Maruyama method for the approximate numerical solution of a stochastic differential equation (SDE), this is correct as it is.

Comment 6: *I would suggest moving the section on data quality control to Methods. (lines 190-201)*

Response: We have moved this paragraph in Appendix, under Methodology/Data Manipulation-Quality Control.

Comment 7: *There seems to be an error in the caption of Figure 3. "The sawtooth oscillations arise because coherent states ($++, --$) are of longer duration.". I think it should be stated here ($+-, -+$).*

Response: Corrected

Comment 8: *The authors sometimes refer to the parameter tau as noise, and sometimes as noise precision. I think the term noise precision should be used throughout the text.*

Response: The term noise refers to the biological noise and noise precision refers to the "statistics" noise of the model parameter τ.

Comment 9: *In Table 2, it is unclear what the labels Significant, Strong, Decisive, Significant+. mean.*

Response: We have clarified this in paragraph "Model Selection" and in the caption of Table 2, which is now moved to the appendix as Table A1.

Comment 10: *A short sentence should be added in the main text explaining how kinetochore pairing was determined, or at least the Methods should be cited.*

Response: This sentence existed in our main text, but we have highlighted it, for clarity.

Comment 11: *I think there is an error in the order of the models in this sentence "In RPE1 cells, 24.4% of sister kinetochores have significant asymmetry, with the majority (42.6% of asymmetric pairs) preferring asymmetry in v−v+ while 87.7% have significant asymmetry in v−" (lines 263-265).*

Response: What we meant here is that the majority of the asymmetric kts 87.7% are asymmetric in v_-, by preferring either of $M^{\alpha}_{v_-}$, $M^{\alpha}_{v_-v_+}$, $M^{\alpha}_{v_-v_+\tau}$) models. We have clarified this in the text.

Comment 12: *In Figure 6, the captions for Figures 6A-D are missing*

Response: We have improved all the figures in the text.

Comment 13: *In the second term in equation 3, the δt term is missing*

Response: Thank you for finding the typo. Addressed.

Comment 14: *I believe, if scaling due to the drag coefficient is to be properly investigated, $tau^1/2$ should be used in Figure 7B, as already stated in the main text.*

Response: We have replaced τ with $\tau^{1/2}$ in Figure 7B and similarly in Figure 10B.

Comment 15: *In equation (4) I think tau should be replaced with tau_0.*

Response: Thank you for finding the typo. Addressed.

Comment 16: *The formulas below lines 409 and 411 should be enumerated.*

Response: As we have many equations, we only enumerate the equations that are referenced in the text.

Comment 17: *Figures 8A and 8B are not consistent with Table 5. For example, 28.1% of the sisters in Figure 8A prefer the v-time dependence, while 13% of the sisters in Table 5 prefer this same dependence.*

Response: We have added some lines in the main text and the captions of Figure 8 to better explain. The difference between the table and figure is that one refers to the total numbers while the latter has the network preference with percentages of the parent nodes.

Comment 18: *There is a typo in the caption of Figure 8, "time dependence of p1 relative to the initial value of parameter p1". The initial parameter should be labeled* p_0 .

Response: Addressed.

Comment 19: *I suggest to include in the caption in Figures 8C-E what "significant" and "non-significant" means.*

Response: Addressed.

Comment 20: *The points representing the data in Figure 9 should be of equal size. The caption in Figure 6B states that the gray lines show percentiles, but they are actually dashed lines.*

Response: Addressed.

Comment 21: *I suggest to label the trajectory of the kinetochore pair in Figure 11C when a reversal event occurs.*

Response: We added 2 red arrows indicating the reversal event, but please note that we have moved this figure to appendix and it is now labelled as Appendix Figure D1.

Comment 22: $\sqrt{d}t$ *is missing in equation (5). I would suggest using* δt *and* δX *instead of* dt *and* dx *for consistency with other equations in the main text.*

Response: We have changed the equation from sde to the form of the previous equations to be consistent.

Comment 23: *There is a typo in the matrix P(t) after line 511. qA is written without a time dependence in the fourth column.*

Response: Thank you. Added the t dependence.

Comment 24: *I suspect that the* $v_{anaphase}$ *and* $|v-|$ *histograms were swapped in Fig. 12E, according to the main text, where it is stated that on average* v_a *is less than* $v-$*.*

Response: Thank you for spotting this error. We have swapped the legend. Please note that we have moved the anphase section to Appendix D.

Comment 25: *How are the radial margins of Figs. 12C and 12D defined, given the fact that the kinetochore clusters are moving?*

Response: The radial margins are defined on the time average of the metaphase component of the trajectory where the trajectories have fairly constant projected (onto plate) metaphase plate positioning.This is stated in the figure caption.

Comment 26: *I strongly recommend enumerating all equations in section C (Methodology).*

Response: As we have many equations, we only enumerate the equations that are referenced in the text.

Comment 27: *Regarding the expressions after line 16 in section C: I assume that* $tau^(n-1)$ *and* $2pi^-(n-1)$ *should carry an additional power of* $\frac{1}{2}$ *. I also think that the variances of the individual Gaussian factors should be* $\delta t/tau$ *instead of* $1/tau$*.*

Response: Thank you for correcting our typos.

Comment 28: *I think the expression for the log-likelihood (after Equation 9 in section C) is wrong, because there should be the logarithm of the sum instead of the sum only. I also don't see where these log-likelihoods are used.*

Response: Thank you for finding this typo. We added the logarithm. We are using log-likelihoods in Bayes Factors and when inferring the parameters in our algorithms as they are more numerically stable.

Comment 29: *After line 31 in section C, the expression for* $\xi_{i,t} P(x1 : t-1)$ *has two repeated lines. After line 32, the first line of the expression* $\xi_{i,t}$ *seems incorrect, and this expression should start with the second line, which seems correct.*

Response: We have deleted the equation and now reads better.

Comment 30: *I'm not sure why the proportionality relations are given for the transition probability between hidden states, equation (10) in the methodology section. As I understand it, these transition probabilities need to be calculated in full form, and not only as proportionality relations, in order to calculate the probability of a particular sequence of hidden states.*

Response: The backward probability is defined relative to the expression on the right-hand side. While we cannot derive a closed-form solution for the transition probabilities, we will compute the proportionality/normalizing constant (that is the inverse of the integral of the right-hand sight relation) numerically to obtain the full probability values.

We thank once again the reviewers for their insightful comments. We hope our revisions adequately address all concerns. Please let us know if any further modifications are required.
