# Supplementary material for: Bayesian data driven modelling of kinetochore dynamics: Space-time organisation of the human metaphase plate
Source: PLoS Comput Biol. 2026 Jan 22;22(1):e1013884. doi: 10.1371/journal.pcbi.1013884 (PMC12935310; doi:10.1371/journal.pcbi.1013884)
Supplement: Attachment — Submitted filename: Responses_to_reviewers.pdf [file pcbi.1013884.s004.pdf]

Response to Reviewers

Dear Editor and Reviewers,

Below, we provide detailed response to each the comments from Reviewers 2 and 3 (reviewer 1 had no additional comments). We provide both a clean copy and a copy with marked changes.

Response to Reviewer 2

The authors have carefully revised according to the review provided. I appreciate their efforts to improve the quality of the paper. I recommend the manuscript for publication. Below are a few minor comments:

Comment 1: *The term 'viscose' should be changed to 'viscous' wherever applicable.*

Response: We have changed all the "viscose" to "viscous".

Comment 2: *In the section in line 1076-1099, some sentences are dense and could benefit from restructuring for enhanced clarity.*

Response: We have re-written that paragraph.

Response to Reviewer 3

The authors have significantly improved the readability and flow of the text, as well as the quality of figures. The core ideas of the manuscript are well arranged, and are easier to follow than in the original version. I find the introduction of oscillator quality as a 4th dimension for describing the kinetochore heterogeneity particularly appealing. I appreciate the authors' effort in developing and properly linking the multitude of imaging, statistical and numerical methods to reveal how the kinetochore movement is regulated within the metaphase of an individual cell. I also appreciate the inclusion of the newly introduced figure on the hierarchy of models, which makes their approach easier to grasp. There are still some minor issues I would ask the authors to address:

Comment 1: *I still think Eq. (1) is inconsistent with Eqs. (3) and (4). In Eq. (1), the increment in time appears in the denominator on the left-hand side, and therefore there should be the inverse of the square root of the time increment multiplying the noise term on the right-hand side. What am I missing here?*

Response: Thank you for pointing that out, we have now corrected that formula.

Comment 2: *Line 491 states that pushing and pulling forces between the asymmetric sisters are correlated: a weaker pushing force corresponds to a stronger pulling force and vice versa. This is evident from Fig. 5D. The term correlation is used in subsection 3.4 (line 492), whereas the term anti-correlation is used in subsection 3.8 (line 757) and in the Discussion (line 917). For clarity, I suggest using consistent terminology throughout the text.*

Response: We have used anti-correlation throughout.

Comment 3: *In part C (Methodology), the symbol tau_0 appears alongside tau in the equation below line 73. Shouldn't only tau appear here, since there is no temporal dependence? It appears that there is a discrepancy in the exponents of tau_0 and 2 Pi between the equations below lines 73 and 75, respectively. It is unclear why there is the product over the index k in both of these equations, since they refer only to a single sister.*

Response: Thank you for finding all the typos. τ_0 is replaced with τ and fixed in both equations. Also, the likelihood equation that we have quoted was for both sisters.

Comment 4: *The definition of L(x) above line 88 seems inconsistent with L(x) above line 93 in the Methods section. Also, it seems that above line 88, it should be the logarithm of the sum, and not the sum of logarithms.*

Response: We have corrected the likelihood. Thank you

Comment 5: It seems that in the set of equations below line 92, part C, which calculates the forward probabilities, there should be $P(x_{1:t-1})/P(x_{1:t})$ instead of just $P(x_{1:t-1})$.

Response: Thank you for finding this typo. It is now correct.

Comment 6: I do not understand the meaning of the symbol s_{t+i}, in the second of the set of equations below line 104. This symbol was not introduced before.

Response: Thank you for catching this typo. It now reads σ_{t+1}.

Comment 7: Overall I find the updated version well suited for publication in your journal, and I believe the results presented by the authors will significantly advance the understanding of how already identified mechanisms (microtubule dynamics, chromosome centering, and centromeric stiffness), together with their heterogeneity, give rise to the successful mitosis.

Response: Thank you for the comments! The feedback helped us to improve a lot our manuscript!

We thank once again the reviewers for their insightful comments. Please let us know if any further modifications are required.
