# Supplementary material for: Bayesian data driven modelling of kinetochore dynamics: Space-time organisation of the human metaphase plate
Source: PLoS Comput Biol. 2026 Jan 22;22(1):e1013884. doi: 10.1371/journal.pcbi.1013884 (PMC12935310; doi:10.1371/journal.pcbi.1013884)
Supplement: Attachment — Submitted filename: Response.docx [file pcbi.1013884.s005.docx]

18^th^ Dec 2025

**RE: PCOMPBIOL-D-25-00039R2**

PLOS Computational Biology.

Dear Professors Chen (Academic Editor) and Papin (Editor-in-Chief),

We are resubmitting our manuscript PCOMPBIOL-D-25-00039R2, "*Bayesian data driven modelling of kinetochore dynamics: space-time organisation of the human metaphase plate*". We have made substantial changes along the lines suggested that we feel substantially improve the readability and accessibility of the manuscript. In particular, we have moved some material to the Methods to retain the flow of the narrative in the main text, and we have overhauled the phrasing throughout. The results and content have not changed.

We did not use AI-based tools in this rewrite.

We hope these changes meet your requirements for publication in PLoS Computational Biology.

Yours sincerely,

Nigel Burroughs.

**Professor Nigel Burroughs**

**Zeeman Institute and Mathematics Institute.**
